# Supplementary material for: Neocortex saves energy by reducing coding precision during food scarcity
Source: Neuron. 2022 Jan 19;110(2):280–296.e10. doi: 10.1016/j.neuron.2021.10.024 (PMC8788933; doi:10.1016/j.neuron.2021.10.024)
Supplement: Document S1. Figures S1–S7 and Tables S1 and S2 [file mmc1.pdf]

Neuron, Volume *110*

Supplemental information

Neocortex saves energy by reducing coding precision during food scarcity

Zahid Padamsey, Danai Katsanevaki, Nathalie Dupuy, and Nathalie L. Rochefort

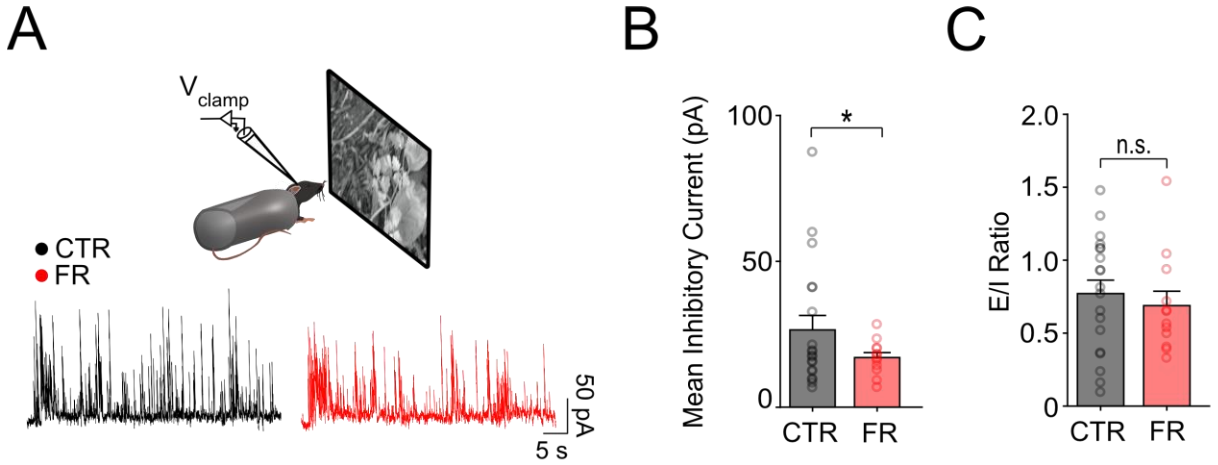

Figure S1. Excitatory/inhibitory balance of postsynaptic currents is preserved with food restriction. Related to Figure 1. (A) Sample inhibitory current traces recorded in voltage clamp during presentation of natural scenes. (**B)** Mean inhibitory current, calculated as the rate of inhibitory charge transfer (t-test; n= 19 CTR and 12 FR cells). (**C)** Mean excitatory/inhibitory (E/I) ratio (t-test; p = 0.56; n = 19 CTR and 12 FR cells). *p<0.05. Error bars are S.E.M.

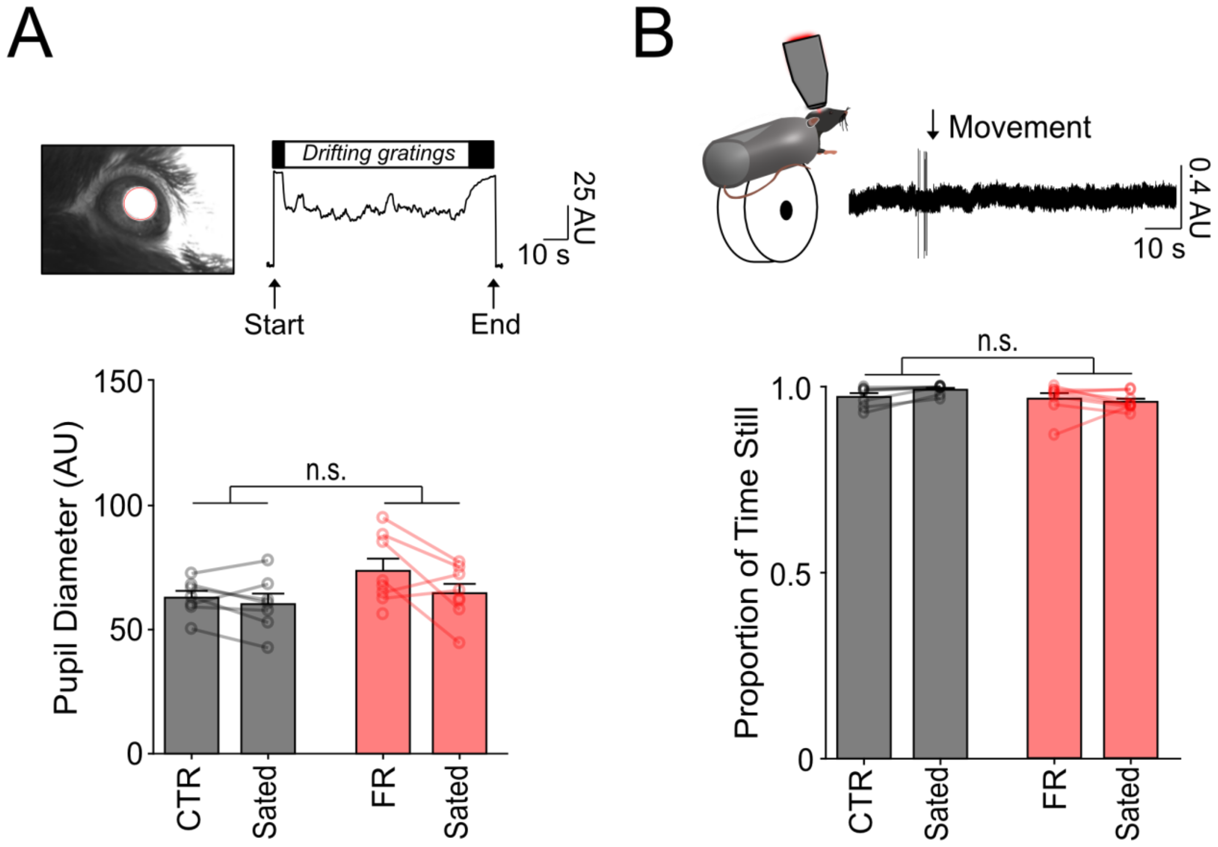

Figure S2. Food restriction does not impact behavioral state as measured by pupil size and fidgets/postural adjustment. Related to all Figures. (A) Top: Sample tracking and detection of pupil diameter (red circle) during presentation of drifting gratings. Bottom: Mean pupil diameter was unchanged across groups, regardless of whether animals were sated or not (Two-way Repeated Measures ANOVA; CTR group vs. FR group: p= 0.07; CTR group vs. FR group: p= 0.15; n = 7 CTR and 8 FR mice). (**B)** During recording sessions, animals were placed in a cardboard tube and balanced on top of Styrofoam wheel, which was wired to an optical encoder. Postural adjustments and fidgets that triggered subtle movements of the wheel could be detected. The proportion of these events were not significantly different across groups, regardless of whether animals were sated or not (Two-way Repeated Measures ANOVA; CTR group vs. FR group: p = 0.53; CTR sated vs. FR sated: p = 0.13; n = 7 and 8 animals). Error bars are S.E.M.

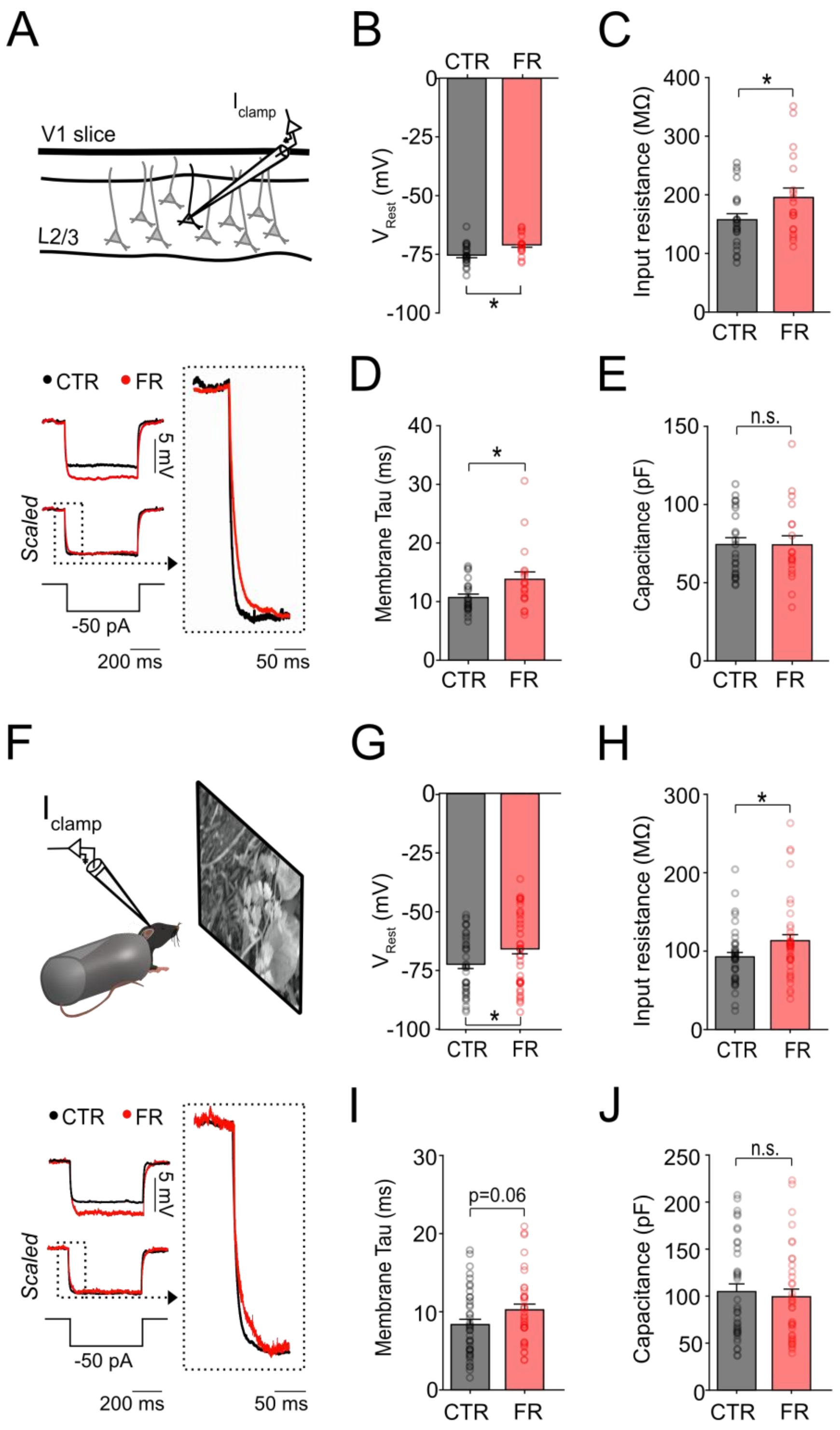

Figure S3: ***Ex vivo*** **recordings also show increased input resistance and resting membrane potential depolarization of V1 layer 2/3 neurons from food-restricted mice. Related to Figure 1 and 3. (A)** Top: Schematic of current clamp recordings *ex vivo*. Bottom: Sample membrane potential traces in response to hyperpolarizing current. The first set of traces highlights group differences in input resistance. The scaled traces have been normalized to peak and overlaid to examine group differences in membrane decay. The region denoted by a broken rectangle is magnified on the right to emphasize group differences in membrane decay. **(B)** Mean resting membrane potential (t-test; n = 23 CTR and 19 FR cells). (**C)** Mean input resistance (t-test; n = 23 CTR and 19 FR cells). (**D)** Membrane tau (t-test; p=0.97; n=23 CTR and 19 FR cells). (**E)** Mean capacitance (t-test; p=0.97; n = 23 CTR and 19 FR cells).

(F) Top: Schema of current clamp recording *in vivo*. Bottom: Sample membrane potential traces in response to hyperpolarizing current. The first set of traces highlights group differences in input resistance. The scaled traces have been normalized to peak and overlaid to examine group differences in membrane decay. The region denoted by a broken rectangle is magnified on the right to emphasize group differences in membrane decay. **(G)** Mean resting membrane potential (t-test; n = 40 CTR and 37 FR cells). Data from Figure 3F. **(H)** Mean input resistance (t-test; n = 40 CTR and 37 FR cells). Data from Figure 3B. **(I)** Membrane tau (t-test; p=0.06; n=40 CTR and 37 FR cells). **(H)** Mean capacitance (t-test; p=0.64; n = 40 CTR and 37 FR cells). *p<0.05. Error bars are S.E.M.

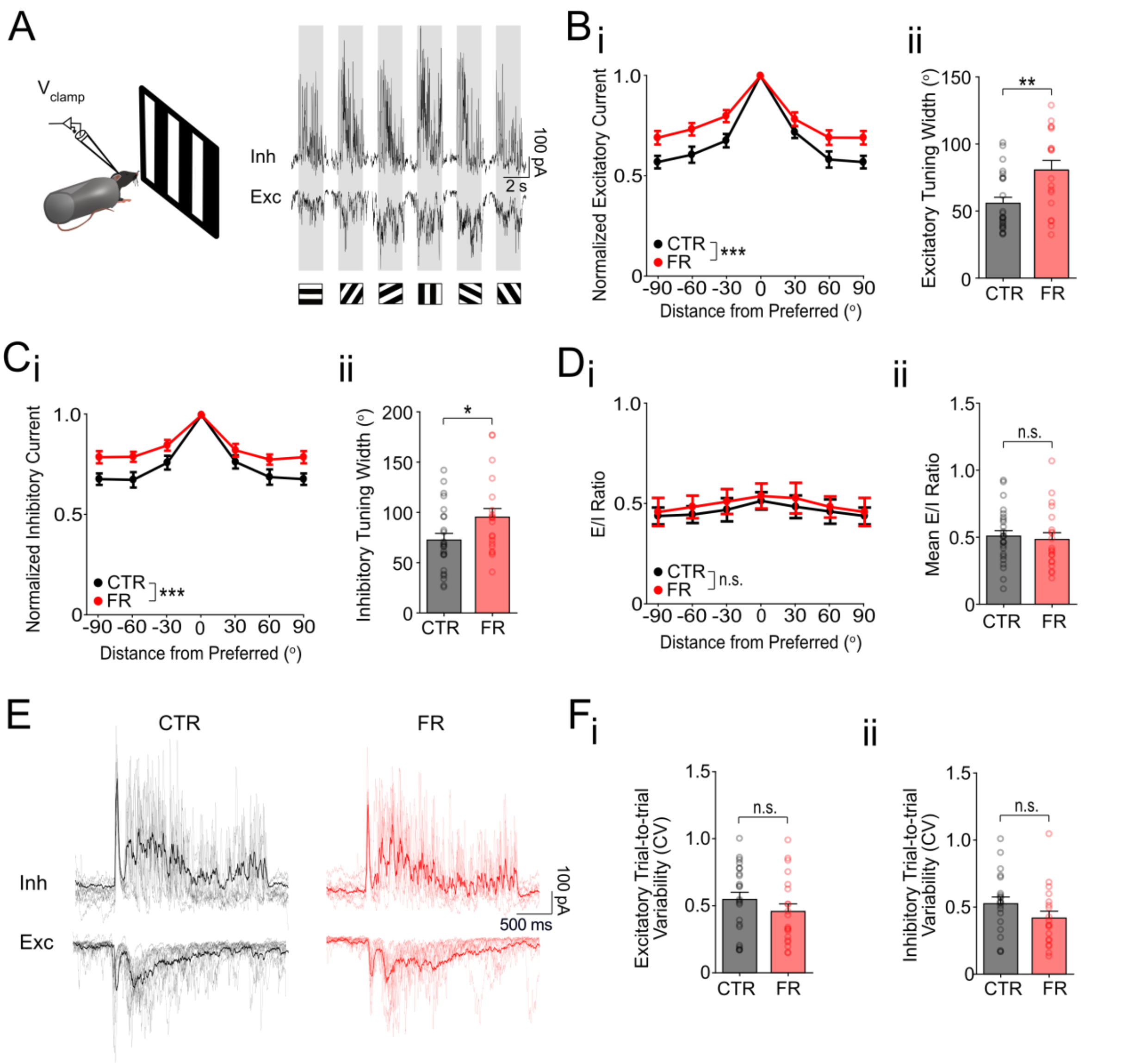

Figure S4: Food restriction broadens the orientation tuning of excitatory and inhibitory presynaptic inputs, but does not impact their variability. Related to Figure 4. (A) Left: Schema of voltage-clamp recordings. Right: Sample excitatory (Exc) and inhibitory (Inh) currents evoked by drifting gratings. **(Bi)** Orientation tuning curve of excitation, normalized to preferred stimulus response. Note that -90° and +90° conditions correspond to the same visual stimulus. (Two-way Repeated Measures ANOVA; CTR group vs. FR group is shown; n = 25 CTR and 20 FR cells). **(Bii)** Mean excitatory tuning width (t-test; n = 25 CTR and 20 FR cells). (**Ci)** Orientation tuning curve of inhibition, normalized to preferred stimulus response. (Two-way Repeated Measures ANOVA; CTR group vs. FR group is shown; n= 25 CTR and 20 FR cells). The subthreshold tuning of inhibitory input is consistent with previous studies. **(Cii)** Mean inhibitory tuning width (t-test; 25 CTR and 20 FR cells). **(Di)** Orientation tuning curve of excitatory/inhibitory (E/I) ratio (Two-way Repeated Measures ANOVA; CTR group vs. FR group: p = 0.34; n= 25 CTR and 20 FR cells). **(Dii)** Mean E/I ratio, averaged across orientations (t-test; p = 0.70; 25 CTR and 20 FR cells). (**E)** Sample traces depicting the trial-to-trial variability (faded traces) and mean (bold traces) of stimulus-evoked excitatory (Exc) and inhibitory (Inh) currents. **(Fi)** Mean co-efficient of trial-to-trial variability (CV) for excitatory currents (t-test; p = 0.63; n = 19 CTR and 17 FR cells). (**Fii)** Mean co-efficient of trial-to-trial variability for inhibitory currents (standard deviation/mean) (t-test; p=0.14; n = 21 CTR and 19 FR cells). *p<0.05; ** p< 0.01; *** p<0.001. Error bars are S.E.M.

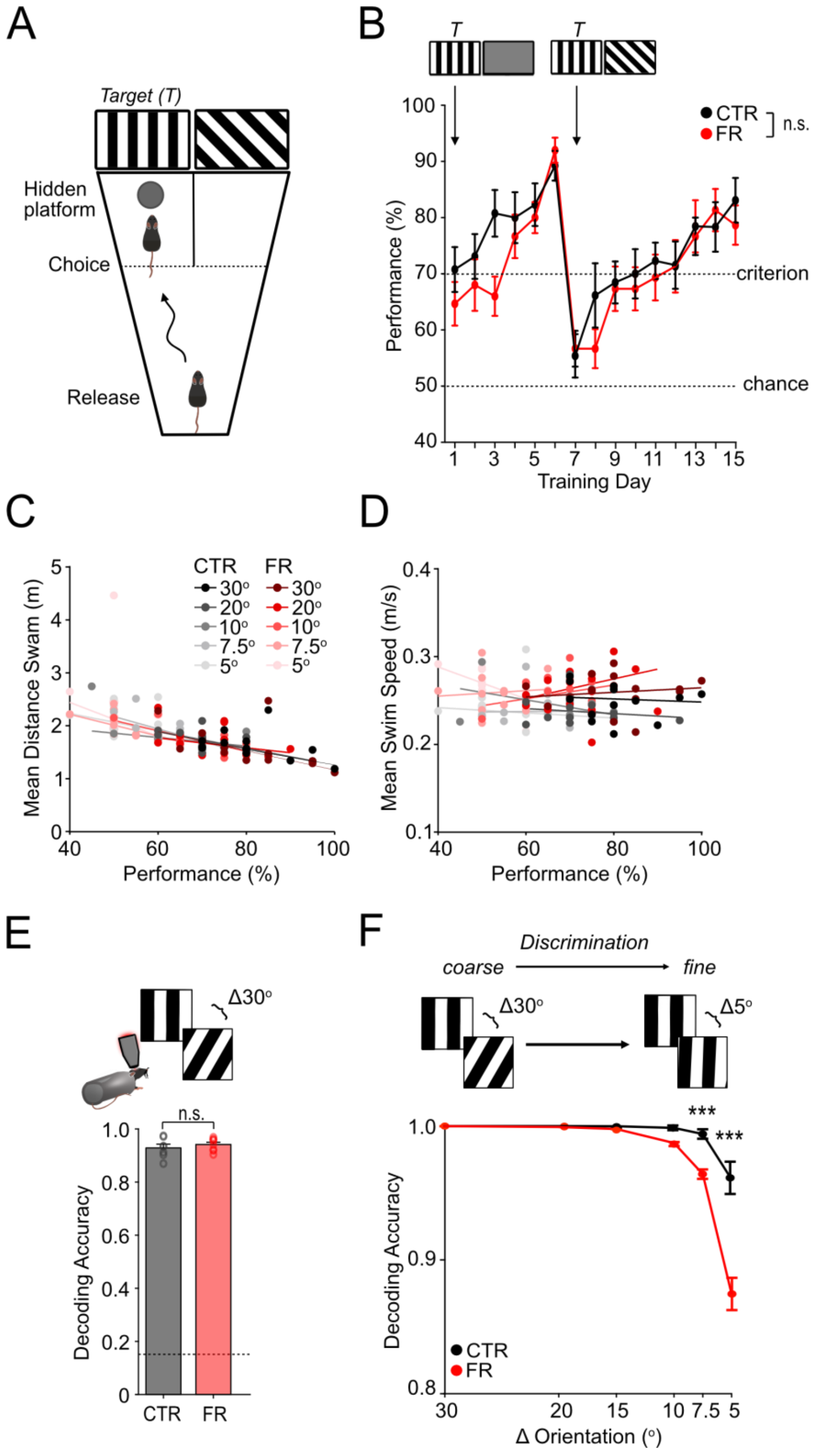

Figure S5: Acquisition of the visual discrimination task and model-based predictions of visual discriminability from V1 calcium imaging data. Related to Figure 6. (A) Schematic for behavioral experiments. Animals were placed in a modified water Y-maze and had to choose the arm associated with a target grating in order to reach a hidden platform and escape the water. (**B)** Task acquisition. A vertical drifting grating was the target throughout the task. On Day 1-6 animals were given a vertical drifting grating (target stimulus) and a grey screen to learn the association of grating-platform. On Day 7-15, they were given the same vertical drifting grating (90°, target stimulus) and a 45° oriented drifting grating (non-target stimulus). Criterion for inclusion in subsequent behavioral tasks was

performance of $\geq$70%. (Mixed-effects Model (REML); CTR group vs. FR group; p = 0.08; n = 13 CTR and 15 FR mice). (**C)** Mean distance swam as a function of behavioral performance. Each point is the mean distance swam for a single animal averaged across trials for a defined discrimination difficulty (angle difference between target and non-target gratings of 30°, 20°, 10°, 7.5° and 5°) plotted against performance at that discrimination difficulty (n = 13 CTR and 15 FR animals per regression; a total of 5 regressions are displayed each for the CTR and FR group, representing 5 discrimination difficulties). There was no group difference in the relationship between swim distance and performance (see Methods for statistics). (**D)** Mean swim speed as a function of behavioral performance. Each point is the mean swim speed for a single animal averaged across trials for a defined discrimination difficulty plotted against performance at that discrimination difficulty (n = 13 CTR and 15 FR animals per regression; a total of 5 regressions are displayed each for the CTR and FR group, representing 5 discrimination difficulties). There was no group difference in the relationship between swim speed and performance (see Methods for statistics). **(E)** Mean accuracy in decoding the correct orientation presented to the mice, amongst 6 equally-spaced orientations (minimum angle difference = 30°) based on layer 2/3 calcium imaging data presented in Figure 6A-C (maximum likelihood decoder; t-test; p=0.43; n=7 CTR and 8 FR mice). Dotted line: chance level. The decoder performed equally well in both groups, suggesting that food restriction did not grossly impact visual discrimination. The same number of neurons was used for decoding across animals. (**F)** Model-based prediction of visual discriminability from calcium imaging data presented in Figure 6A-C. Mean decoding accuracy as a function of discrimination difficulty (*i.e.*, angle difference between orientated gratings, 5–30°). Simulated based on experimental responses to 30° angle differences. Differences in decoding emerged when angle differences were < 10°, consistent with behavioral results on visual discriminability (Figure 6E) (Two-way ANOVA; CTR group vs. FR group: p<0.001; *post hoc* Sidak's tests). *** p<0.001. Error bars are S.E.M.

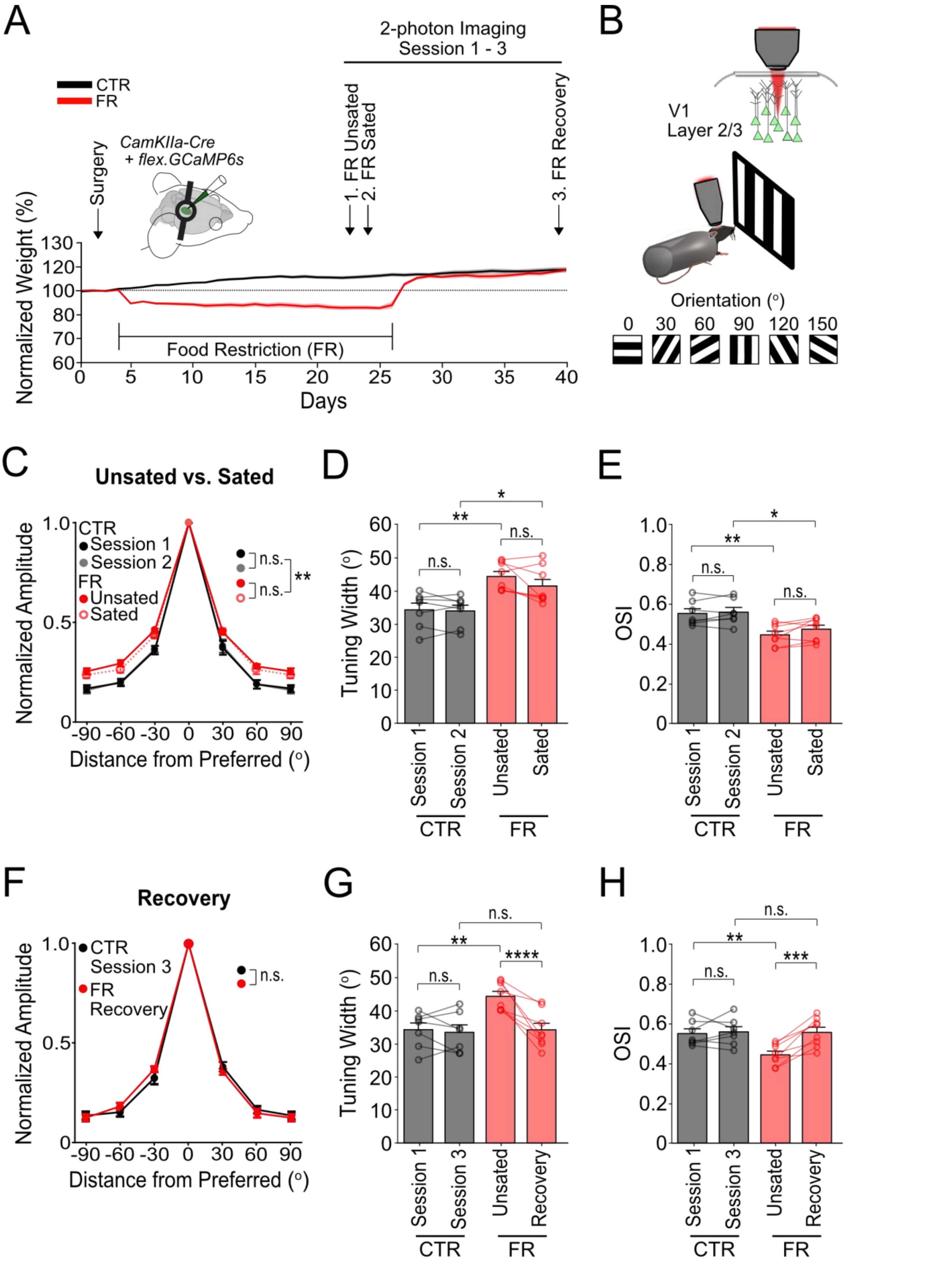

Figure S6: Broadening of orientation tuning under food restriction was not impacted by short-term satiety, and reversed to control values after recovery of bodyweight. Related to Figure 7. (A) Experimental timeline along with animal weight. (**B)** Schema of two-photon imaging during presentation of drifting gratings. (**C)** Mean orientation tuning curves, normalized to the response of

the preferred stimulus. Note that -90° and +90° conditions correspond to the same visual stimulus. (Three-way ANOVA with data from (C) and (F), which includes data from Main Figure 6B; CTR group vs. FR group: p<0.0001; CTR Session 1 vs. CTR Session 2: p>0.99; FR Unsated vs. FR Sated: p=0.32; CTR Session 1 vs. FR Unsated: p<0.001; CTR Session 2 vs. FR Sated: p<0.001; n = 7 CTR and 8 FR mice). (**D)** Mean orientation tuning width for CTR and FR before and after satiety (Two-way Repeated Measures ANOVA run with data in (D) and (G), which includes data from Main Figure 6C, with post hoc Sidak's tests; CTR Session 1 vs. CTR Session 2: p = 0.99; FR Unsated vs. FR Sated: p= 0.17; n = 7 CTR and 8 FR mice). **(E)** Mean orientation selectivity index (OSI, measured as 1-circular variance) for CTR and FR before and after satiety (Two-way Repeated Measures ANOVA run with data in (E) and (H); *post hoc* Sidak's tests; CTR Session 1 vs. CTR Session 2: p = 0.98; FR Unsated vs. FR Sated: p= 0.30; n = 7 CTR and 8 FR mice). (**F)** Mean orientation tuning curves, normalized to the response of the preferred stimulus. (Three-way ANOVA with data from (C) and (F); CTR group vs. FR group: p<0.0001; CTR Session 3 vs. FR Recovery: p>0.99; n = 7 CTR and 8 FR mice). (**G)** Mean orientation tuning widths for CTR and FR after recovery of bodyweight following 10-14 days of *ad libitum* access to food. (Two-way Repeated Measures ANOVA; post hoc Sidak's tests; CTR Session 1 vs. CTR Session 3: p = 0.94; CTR Session 3 vs. FR Recovery: p= 0.98; n = 7 CTR and 8 FR mice). (**H)** Mean orientation selectivity index (OSI, measured as 1-circular variance) for CTR and FR before and after recovery of free feeding bodyweight (Two-way Repeated Measures ANOVA; post hoc Sidak's tests; CTR Session 1 vs. CTR Session 3: p = 0.99; CTR Session 3 vs. FR Recovery: p= 0.99; n = 7 CTR and 8 FR mice). * p< 0.05; ** p<0.01; *** p<0.001; *** p<0.0001. Error bars are S.E.M.

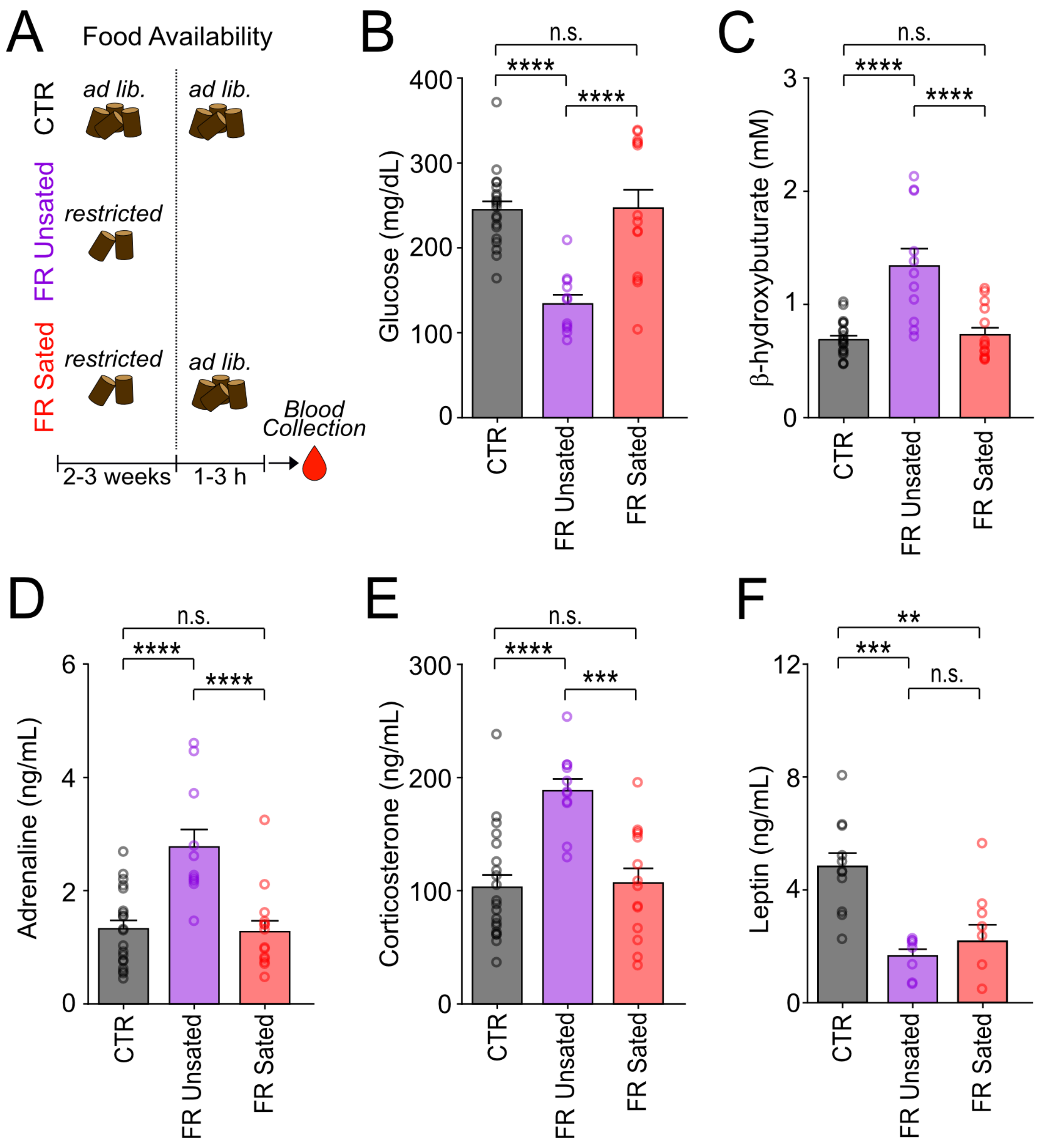

Figure S7. Impact of food restriction and satiety on metabolic state. Related to Figure 7. (A) Experimental schema. Control (CTR) and food-restricted (FR) animals were maintained on *ad libitum* and food-restricted diets (resulting in 15% loss of body weight), respectively, for 2-3 weeks prior to trunk blood collection. Animals in the "FR sated" group were sated with *ad libitum* access to food for 1-3 hours prior to blood collection, "FR unsated" animals were not. **(B)** Mean levels of serum glucose (One-way ANOVA: $p<0.0001$; CTR vs. FR Sated: $p>0.99$; n = 20 CTR, 11 FR Unsated, and 14 FR Sated animals). **(C)** Mean levels of serum β-hydroxybuturate (ketone body) (One-way ANOVA: $p<0.0001$; CTR vs. FR Sated: $p=0.97$; n = 20 CTR, 11 FR Unsated, and 14 FR Sated animals). **(D)** Mean levels of serum adrenaline (One-way ANOVA: $p<0.0001$; CTR vs. FR Sated: $p>0.99$; n = 20 CTR, 11 FR Unsated, and 14 FR Sated animals). **(E)** Mean levels of serum corticosterone (One-way ANOVA: $p<0.0001$; CTR vs. FR Sated: $p>0.99$; n = 20 CTR, 11 FR Unsated, and 14 FR Sated animals). **(F)** Mean levels of serum leptin (One-way ANOVA: $p<0.0001$; CTR vs. FR Sated: $p=0.87$; n = 11 CTR, 8 FR Unsated, and 9 FR Sated animals). Error bars are S.E.M.

Supplemental Table S1 – Model parameters for all groups relative to control. Related to Figure 5.

Group	Depolarization of the V_{Rest} from control	Normalized leak: $g_L^{group}/g_L^{control}$	Normalized synaptic conductance: $g_0^{group}/g_0^{control}$
Control (CTR)	+0 mV	1	1
Increased R_m	+0 mV	0.79	0.79
V_{Rest} depolarized	+5 mV	1	0.81
Food-restricted (FR)	+5 mV	0.79	0.64

Resting membrane potential (V_{Rest}); Resting leak conductance (g_L); Synaptic conductance at rheobase (g_o)

Supplemental Table S2 – Hodgkin-Huxley-type neuron model parameters (control). Related to Figure 5.

Parameters:		
C_m	Membrane capacitance	1 μF/cm^2
g_L	Leak conductance (inverse input resistance)	0.1 mS/cm^2
E_L	Leak reversal potential	-75 mV
E_{rest}	Resting membrane potential	-75 mV
g_{Na}	Na_v maximal conductance	35 mS/cm^2
E_{Na}	Na_v reversal potential	55 mV
g_K	K_v maximal conductance	4 mS/cm^2
E_K	K_v reversal potential	-90 mV
g_X	Subthreshold channel maximal conductance	0.18 mS/cm^2
γ	Single-channel conductance (only for g_X channel)	20 pS
