# Supplementary material for: Neocortex saves energy by reducing coding precision during food scarcity
Source: Neuron. 2022 Jan 19;110(2):280–296.e10. doi: 10.1016/j.neuron.2021.10.024 (PMC8788933; doi:10.1016/j.neuron.2021.10.024)
Supplement: Document S2. Article plus supplemental information [file mmc2.pdf]

Neuron

Article

Neocortex saves energy by reducing coding precision during food scarcity

Graphical abstract

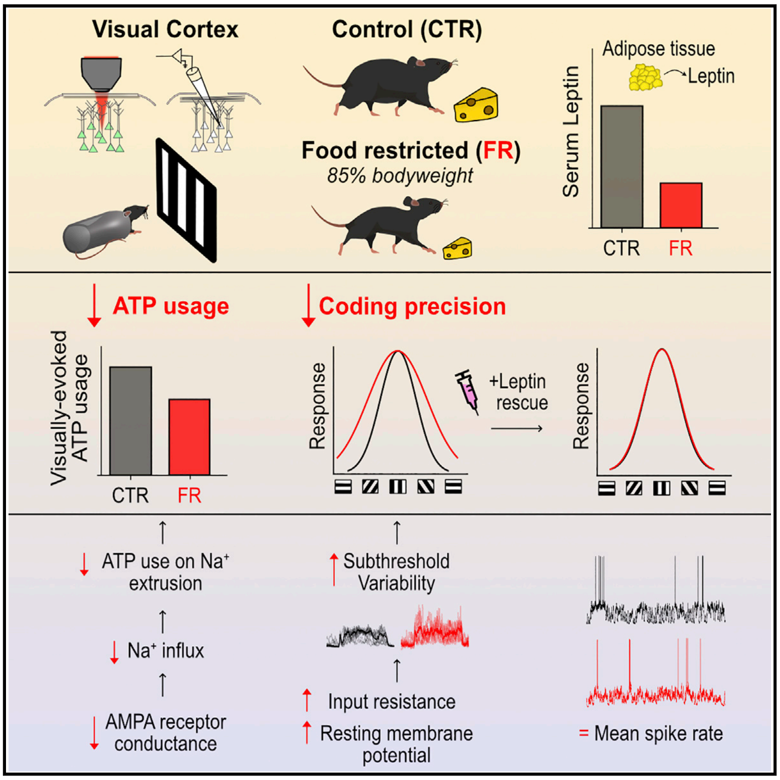

Authors

Zahid Padamsey, Danai Katsanevaki, Nathalie Dupuy, Nathalie L. Rochefort

Correspondence

zahid.padamsey@ed.ac.uk (Z.P.), n.rochefort@ed.ac.uk (N.L.R.)

In brief

How does the metabolically expensive mammalian brain adapt to food scarcity? Padamsey et al. show that under food restriction, mouse visual cortical neurons save ATP by decreasing excitatory currents. Compensatory mechanisms preserve spike rate but decrease coding precision of visual information. Supplementation with the fat mass-regulated hormone leptin restores coding precision.

Highlights

- Food restriction results in ATP savings via reduced AMPAR currents in visual cortex
- Concurrent increases in input resistance and resting potential preserve spike rate
- These changes amplify response variability, leading to broader orientation tuning
- Supplementation with the adipocyte-hormone leptin restores visual coding precision

Padamsey et al., 2022, Neuron *110*, 280–296
January 19, 2022 © 2021 The Authors. Published by Elsevier Inc.
https://doi.org/10.1016/j.neuron.2021.10.024

CellPress
OPEN ACCESS

Neuron

Article

Neocortex saves energy by reducing coding precision during food scarcity

Zahid Padamsey,[1,*] Danai Katsanevaki,[1] Nathalie Dupuy,[1] and Nathalie L. Rochefort[1,2,3,*]
[1]Centre for Discovery Brain Sciences, School of Biomedical Sciences, University of Edinburgh, Edinburgh EH8 9XD, UK
[2]Simons Initiative for the Developing Brain, University of Edinburgh, Edinburgh EH8 9XD, UK
[3]Lead contact
*Correspondence: zahid.padamsey@ed.ac.uk (Z.P.), n.rochefort@ed.ac.uk (N.L.R.)
https://doi.org/10.1016/j.neuron.2021.10.024

SUMMARY

Information processing is energetically expensive. In the mammalian brain, it is unclear how information coding and energy use are regulated during food scarcity. Using whole-cell recordings and two-photon imaging in layer 2/3 mouse visual cortex, we found that food restriction reduced AMPA receptor conductance, reducing synaptic ATP use by 29%. Neuronal excitability was nonetheless preserved by a compensatory increase in input resistance and a depolarized resting potential. Consequently, neurons spiked at similar rates as controls but spent less ATP on underlying excitatory currents. This energy-saving strategy had a cost because it amplified the variability of visually-evoked subthreshold responses, leading to a 32% broadening of orientation tuning and impaired fine visual discrimination. This reduction in coding precision was associated with reduced levels of the fat mass-regulated hormone leptin and was restored by exogenous leptin supplementation. Our findings reveal that metabolic state dynamically regulates the energy spent on coding precision in neocortex.

INTRODUCTION

Despite constituting less than 2% of the body's mass, the human brain consumes approximately 20% of total caloric intake, with 50% of the energy being used by cortex (Herculano-Houzel, 2011). The majority of this energy is spent by neurons to reverse the ion fluxes associated with electrical signaling via Na^+/K^+ ATPase (Attwell and Laughlin, 2001; Harris et al., 2012). Excitatory synaptic currents and action potentials are particularly costly in this regard, accounting for approximately 57% and 23% of the energy budget for electrical signaling in gray matter, respectively (Harris et al., 2012; Sengupta et al., 2010). Given this cost, and the scarcity of resources, the brain is thought to have evolved an energy-efficient coding strategy that maximizes information transmission per unit energy (i.e., ATP) (Barlow, 2012; Levy and Baxter, 1996). This strategy accounts for a number of cellular features, including the low mean firing rate of neurons and the high failure rate of synaptic transmission, as well as higher order features, such as the structure of neuronal receptive fields (Albert et al., 2008; Attwell and Laughlin, 2001; Harris et al., 2015; Levy and Baxter, 1996; Olshausen and Field, 1997; Sterling and Laughlin, 2015). Scarcity of food, therefore, appears to have strongly sculpted information coding in the brain throughout evolution.

Energy intake is not fixed but can vary substantially across individuals, environments, and time (Hladik, 1988; Knott, 1998). Given that the brain is energy limited, one hypothesis is that in times of food scarcity, neuronal networks should save energy by reducing information processing. There is some evidence to suggest that this is the case in invertebrates (Kauffman et al., 2010; Longden et al., 2014; Plaçais et al., 2017; Placais and Preat, 2013). In *Drosophila*, food deprivation inactivates neural pathways required for long-term memory to preserve energy (Plaçais et al., 2017; Placais and Preat, 2013). Experimental reactivation of these pathways restores memory formation but significantly reduces survival rates (Placais and Preat, 2013). Similar memory impairments are seen with reduced food intake in *C. elegans* (Kauffman et al., 2010). Moreover, in blowfly, food deprivation reduces visual interneuron responses during locomotion, consistent with energy savings (Longden et al., 2014). However, it remains unclear whether and how the mammalian brain, and cortical networks in particular, regulate information processing and energy use in times of food scarcity.

Here we used the mouse primary visual cortex (V1) as a model system to examine how food restriction affects information coding and energy consumption in cortical networks. We assessed neuronal activity and ATP consumption using whole-cell patch-clamp recordings and two-photon imaging of V1 layer 2/3 excitatory neurons in awake, male mice. We found that food restriction, resulting in a 15% reduction of body weight, led to a 29% reduction in ATP expenditure associated with excitatory postsynaptic currents, which was mediated by a decrease in single-channel AMPA receptor (AMPAR) conductance. Reductions in AMPAR current were compensated by an increase in input resistance and a depolarization of the resting membrane potential, which preserved neuronal excitability; neurons were therefore able to generate a comparable rate of spiking as controls, while spending less ATP on the underlying excitatory currents. This energy-saving strategy, however, had a cost to coding

 © 2021 The Authors. Published by Elsevier Inc.
This is an open access article under the CC BY license (http://creativecommons.org/licenses/by/4.0/).

CellPress
OPEN ACCESS

precision. Indeed, we found that an increase in input resistance and depolarization of the resting membrane potential also increased the subthreshold variability of visual responses, which increased the probability for small depolarizations to cross spike threshold, leading to a broadening of orientation tuning by 32%. Broadened tuning was associated with reduced coding precision of natural scenes and behavioral impairment in fine visual discrimination. We found that these deficits in visual coding under food restriction correlated with reduced circulating levels of leptin, a hormone secreted by adipocytes in proportion to fat mass (Baile et al., 2000), and were restored by exogenous leptin supplementation. Our findings reveal key metabolic state-dependent mechanisms by which the mammalian cortex regulates coding precision to preserve energy in times of food scarcity.

RESULTS

We characterized the impact of food restriction on ATP use and coding precision in layer 2/3 neurons of the V1 of awake mice. Adult male mice were either given *ad libitum* access to food (control group) or were food-restricted to 85% of their body weight over the course of 2–3 weeks (food-restricted group) (Figure 1A). All animals were given *ad libitum* access to food for 1–3 h prior to recording until sated. This allowed us to study the impact of long-term calorie restriction independent of the short-term metabolic and stress-related changes associated with hunger.

Food restriction results in reduced excitatory synaptic currents and ATP use but preserved spike rate

We first examined ATP use in neocortex, which we hypothesized would be reduced with food restriction to preserve energy. Energy expenditure for neuronal signaling is principally associated with the reversal of Na^+ influx via the Na^+/K^+ ATPase. Therefore, ATP expenditure can be estimated by measuring the Na^+ flux associated with electrical signaling, on the basis that one ATP is required to extrude three Na^+ (Attwell and Laughlin, 2001; Harris et al., 2012; Sengupta et al., 2010). We estimated the Na^+ influx associated with excitatory postsynaptic currents, a major source of ATP consumption (57%), using whole-cell voltage-clamp recordings of layer 2/3 neurons (Harris et al., 2012, 2015). We performed recordings in awake, head-fixed mice viewing a movie of natural scenes (Figures 1B and 1C). Cells were clamped at −70 mV to isolate excitatory postsynaptic currents from inhibitory GABAergic currents. We found that excitatory currents triggered by visual stimulation were reduced by 29% in food-restricted animals (Figure 1D; n = 35 control and n = 29 food-restricted cells), corresponding to a 29% reduction in the calculated ATP consumption required to reverse the associated Na^+ influx (Figure 1E) (Attwell and Laughlin, 2001; Harris et al., 2015). However, excitatory/inhibitory balance was unchanged because of a proportional decrease in inhibitory currents (Figure S1). Group differences in synaptic ATP use were not attributable to any potential differences in behavioral state, as assessed by pupil size or body movements (Figure S2), or to any potential differences in stress, as assessed by serum adrenaline and corticosterone levels (Figures S7D and S7E). We also examined energy expenditure associated with action potentials, the second largest source of energy consumption in the brain (23%) (Harris et al., 2012; Sengupta et al., 2010), while mice viewed a natural movie. We recorded action potentials in current clamp (Figure 1F; n = 40 control and n = 37 food-restricted cells) and found no change in action potential waveform (Figure 1F), spike rate (Figure 1G), or membrane capacitance (Figures S3E and S3J) and consequently no change in the corresponding calculated ATP consumption (Figure 1H) (Attwell and Laughlin, 2001; Harris et al., 2015).

To confirm the overall decrease in ATP use in food-restricted mice, we used the established ATeam1.03^{YEMK} transgenic mouse line to image visual cortical ATP levels in awake mice; this line expresses a FRET-based ATP sensor under the Thy1 promoter and has been shown to detect ATP expenditure both *in vivo* in mouse cortex and in neurons *ex vivo* (Figure 1I) (Baeza-Lehnert et al., 2019; Lerchundi et al., 2020; Trevisiol et al., 2017). Cellular ATP levels reflect a balance between the rate of ATP synthesis and expenditure. To isolate ATP use, we locally applied ATP synthesis inhibitors to V1 and imaged the resulting decay of the FRET signal in layer 2/3 during the presentation of a natural movie (n = 10 control and n = 9 food-restricted mice). We found that during visual stimulation, the rate of FRET decay was significantly reduced for food-restricted animals compared with controls, confirming a reduced rate of ATP consumption (Figures 1J and 1K).

Altogether, these findings demonstrate that food restriction leads to reduced ATP use through a reduction in excitatory synaptic current, without a reduction in spike output.

Food restriction results in a reduction in AMPA receptor conductance

We next sought to understand the mechanistic basis underlying the energy-savings reduction in excitatory synaptic currents. We examined whether the reduction in excitatory currents recorded *in vivo* was caused by a change in AMPA receptor (AMPAR) properties, which we assessed *ex vivo.* We found that AMPAR currents in visual cortical slices, evoked by a stimulating electrode placed in layer 2/3, were reduced in food-restricted animals by 34%, comparable with what we observed *in vivo* (Figure 2A; n = 22 control and n = 20 food-restricted cells). This reduction was likely a result of postsynaptic changes in AMPARs, as there was no evidence of presynaptic changes in transmitter release on the basis of measures of paired pulse ratio and the coefficient of variation (CV) of synaptic responses (Figures 2B and 2C). Postsynaptic changes were confirmed by subsequent recordings of AMPAR-mediated miniature synaptic currents in TTX, which showed that the amplitude but not frequency of these currents was reduced (Figures 2D–2F; n = 20 control and n = 20 food-restricted cells). Moreover, mean variance analysis (Traynelis et al., 1993; Yamashita et al., 2003) showed that single-channel AMPAR conductance, as opposed to the total number of AMPARs engaged during synaptic transmission, was reduced by 37% (Figures 2G–2I), sufficient to account for the reduction of synaptic currents, and thus ATP use, observed with food restriction *in vivo*.

A compensatory increase in input resistance and a depolarization of the resting membrane potential preserve spike rate despite a reduction in AMPAR conductance

We next examined why spike rate was not significantly affected by food restriction despite a decrease in AMPAR-mediated

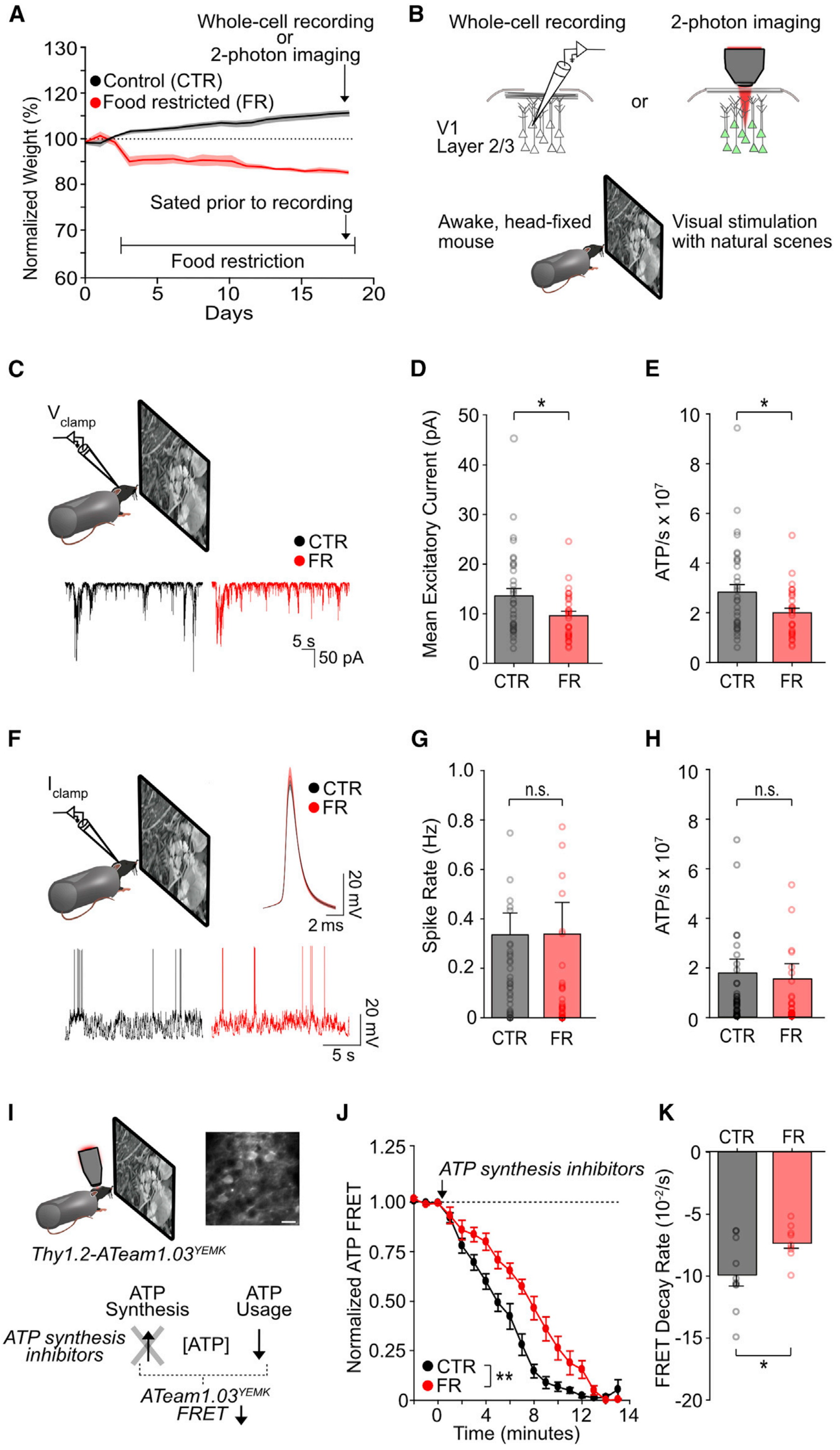

Figure 1. Food restriction results in reduced excitatory synaptic currents and ATP use but preserved spike rate in awake mice

(A) Experimental timeline and animal weight.

(B) Schemata of experimental design and visual stimulation.

(C) Sample excitatory current traces recorded in voltage-clamp of a layer 2/3 neuron during presentation of natural scenes.

(D) Mean excitatory current, calculated as the rate of excitatory charge transfer (t test; n = 35 control and n = 29 food-restricted cells).

(E) Mean ATP consumption rate for excitatory currents (t test; n = 35 control and n = 29 food-restricted cells).

(F) Sample traces recorded in current clamp of a layer 2/3 neuron during presentation of natural scenes. Top right: mean action potential trace from recordings; shaded region denotes the standard error of the mean (n = 40 control and n = 37 food-restricted cells).

(G) Mean spike rate (p = 0.98, t test; n = 40 control and n = 37 food-restricted cells).

(H) Mean ATP consumption rate for spiking (p = 0.78, t test; n = 40 control and n = 37 food-restricted cells).

(I) Top: sample field of view of V1 layer 2/3 neurons in the ATeam1.03^{YEMK} transgenic mouse (scale bar: 10 μm). Bottom: experimental schematic. ATP synthesis inhibitors were used to isolate ATP use, recorded as a decrease in FRET signal.

(J) Normalized ATeam1.03^{YEMK} FRET signal during presentation of natural scenes. ATP consumption was evaluated by imaging the FRET signal after adding ATP synthesis inhibitors (arrow) (two-way repeated-measures ANOVA, control group versus food-restricted group; n = 10 CTR and n = 9 food-restricted animals).

(K) Mean FRET decay rate (t test; n = 10 control and n = 9 food-restricted animals).

*p < 0.05. Error bars are S.E.M. See also Figures S1 and S3.

synaptic currents. Given that excitatory/inhibitory balance was unchanged (Figure S1), two possible compensatory mechanisms could maintain spike output given a lower excitatory input current: (1) an increase in input resistance or (2) a decrease in the amount of depolarization required to reach spike threshold from resting membrane potential (i.e., the distance to spike threshold). We assessed these properties in awake mice using current-clamp recordings and found that food restriction increased neuronal input resistance by 22% (Figures 3A and 3B; n = 40 control and n = 37 food-restricted cells). This did not fully compensate for the 29% reduction in synaptic current, as the mean, visually-evoked subthreshold depolarization was still 17% smaller in food-restricted compared with control animals (Figure 3C). However, we also found that the distance to spike threshold was reduced in food-restricted animals (Figure 3D), driven by a 6–7 mV depolarization of the resting membrane potential; there was no change in the spike threshold (Figures 3F and 3G). As a consequence of both an increased input resistance and a reduced distance

CellPress
OPEN ACCESS

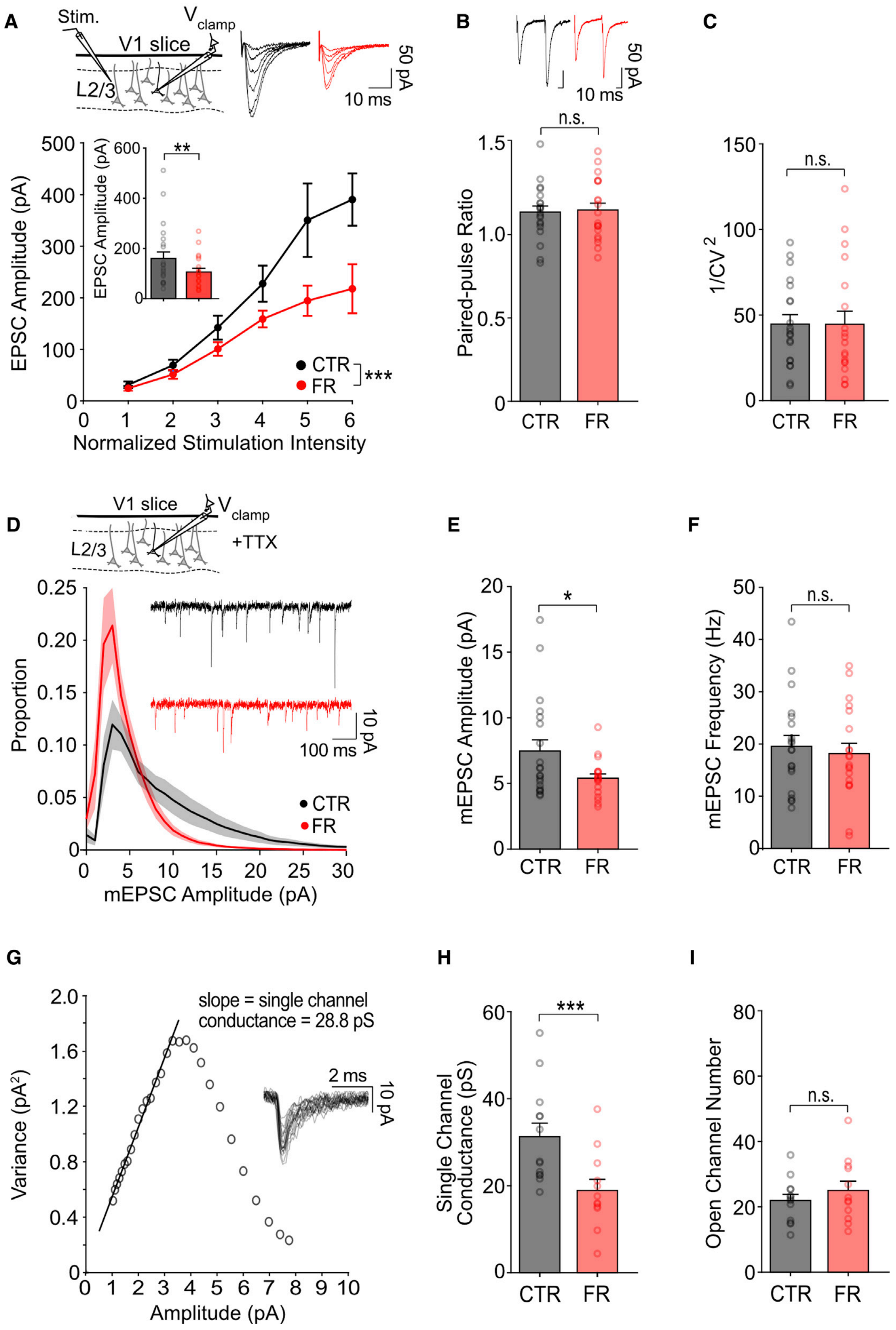

(legend on next page)

to spike threshold, the relative depolarization of visually-evoked activity (i.e., the mean subthreshold depolarization divided by the total distance to spike threshold) in food-restricted animals was the same as in controls (Figure 3E). Both increased input resistance and a depolarized resting membrane potential were also observed in *ex vivo* brain slices from food-restricted animals, in the absence of *in vivo* network activity, consistent with these changes being cell-intrinsic (Figures S3B–S3C).

Using an integrate-and-fire model neuron, we confirmed that increasing the input resistance and depolarizing the resting membrane potential to experimental values enabled synaptic potentials to reach spike threshold despite being driven by 29% less AMPAR current (Figures 3H–3K). Thus, under food-restricted conditions, combining an energy-saving reduction in AMPAR conductance with a compensatory increase in input resistance and a depolarization of the resting membrane potential enabled neurons to maintain comparable spiking rates as controls, but at a reduced ATP cost.

Notably, changes in input resistance and resting membrane potential alone can affect basal ATP use for maintaining the resting membrane potential (Attwell and Laughlin, 2001). However, using previously published calculations (Attwell and Laughlin, 2001), we found that the combined increase in input resistance and depolarization of the resting membrane potential observed with food restriction had no net impact on the estimated ATP consumption at rest (control, $4.91 \pm 0.76 \times 10^8$ ATP/s; food-restricted, $5.17 \pm 0.49 \times 10^8$ ATP/s; $p = 0.78$, t test; n = 40 control and n = 37 food-restricted cells). These compensatory changes therefore did not directly affect energy expenditure but instead acted primarily to preserve excitability under conditions of reduced AMPAR conductance.

Food restriction increases subthreshold variability, resulting in broader orientation tuning of spike output

We have shown that cell-intrinsic changes can save energy without compromising mean spike rate in food-restricted animals. We hypothesized that this energy-saving strategy should affect the encoding of visual information (Attwell and Laughlin, 2001; Harris et al., 2012). To better examine the neuronal coding of visual stimuli, we performed current-clamp recordings of layer 2/3 neurons in the visual cortex of awake mice during the presentation of drifting gratings (Figure 4A; n = 33 control and n = 28 food-restricted cells). We found that the orientation tuning of spike output was broadened by 32% with food restriction, showing that neurons were less selective to stimulus orientation (Figures 4B and 4C). Voltage-clamp recordings revealed that both presynaptic excitatory and inhibitory inputs exhibited broadened orientation tuning in food-restricted mice, such that the excitatory/inhibitory balance was similar to controls (Figures S4A and S4D). As a result, the orientation tuning of the subthreshold depolarization of membrane potential was unchanged in food-restricted mice relative to control mice, indicating that broader orientation tuning in spike output was not directly inherited from presynaptic input (Figures 4E and 4F). The relative levels of subthreshold depolarization, when normalized to the distance to spike threshold, were also comparable in both groups (Figure 4E), despite an overall decrease in the amplitude of visually-evoked depolarization (Figure 4D) in the food-restricted group (consistent with Figures 3C and 3E). Collectively, these findings show that broadened orientation tuning with food restriction cannot be readily explained by changes in presynaptic input. On this basis, we investigated whether intrinsic changes contributed to orientation broadening.

Specifically, we examined whether an alteration in the neuronal input/output function contributed to broadened orientation tuning. To characterize the neuronal input/output function, we examined the relationship between mean membrane depolarization (normalized to the distance to spike threshold) and spike output during visual stimulation. We found that with food restriction, smaller depolarizations were more likely to elicit at least one spike (Figure 4G). The rate of spiking triggered by smaller depolarizations was also increased relative to control animals (Figure 4H). Additionally, we found a reduction in spike rate in response to larger depolarizations, such that overall spike rate was unchanged from control levels (Figures 4H and 4I).

An increase in response variability can account for increased spiking at small depolarizations but not at larger depolarizations (Chance et al., 2002). Consistent with this, we found that food restriction resulted in an increase in the variability of subthreshold responses, measured here as the CV (standard deviation/mean response) of trial-to-trial subthreshold depolarization (Figures 4J and 4K). Surprisingly, no group differences in subthreshold variability were observed with excitatory or inhibitory currents recorded in voltage-clamp, suggesting that the increased

Figure 2. Reduced AMPAR currents are mediated by a reduction in single-channel AMPAR conductance in food-restricted mice

(A) Top left: schema of voltage-clamp recordings of layer 2/3 neurons in V1 slices. A stimulation electrode was placed in layer 2/3. Top right: sample traces of excitatory currents (−70 mV) evoked by stimulation at varying intensities. Bottom: mean excitatory postsynaptic current (EPSC) amplitude evoked by stimulation (two-way repeated-measures ANOVA, control group versus food-restricted group; n = 22 control and n = 20 food-restricted cells). Inset: mean EPSC amplitude evoked by half-maximal stimulation (t test; n = 22 CTR and n = 20 food-restricted cells).

(B) Top: sample EPSC traces in response to paired-pulse stimulation. Bottom: mean paired-pulse ratio ($p = 0.93$, t test; n = 22 control and n = 20 food-restricted cells).

(C) Mean $1/CV^2$ ($p = 0.65$, t test; n = 22 control and n = 20 food-restricted cells).

(D) Top: schema of miniature EPSCs (mEPSCs) recordings in the presence of TTX. Bottom: sample traces recorded at −70 mV and mean distribution of mEPSC amplitudes (n = 20 control and n = 20 food-restricted cells).

(E) Mean mEPSC amplitude (t test; n = 20 control and n = 20 food-restricted cells).

(F) Mean mEPSC frequency ($p = 0.63$, t test; n = 20 control and n = 20 **food-restricted** cells).

(G) Example of mean-variance analysis of mEPSCs (gray traces) recorded in one cell. Single-channel conductance was estimated from the initial slope.

(H) Mean single-channel conductance (t test; n = 13 control and n = 12 food-restricted cells).

(I) Mean open channel number ($p = 0.36$, t test; n = 13 control and n = 12 food-restricted cells).

*$p < 0.05$, **$p < 0.01$, and ***$p < 0.001$. Error bars are S.E.M.

CellPress
OPEN ACCESS

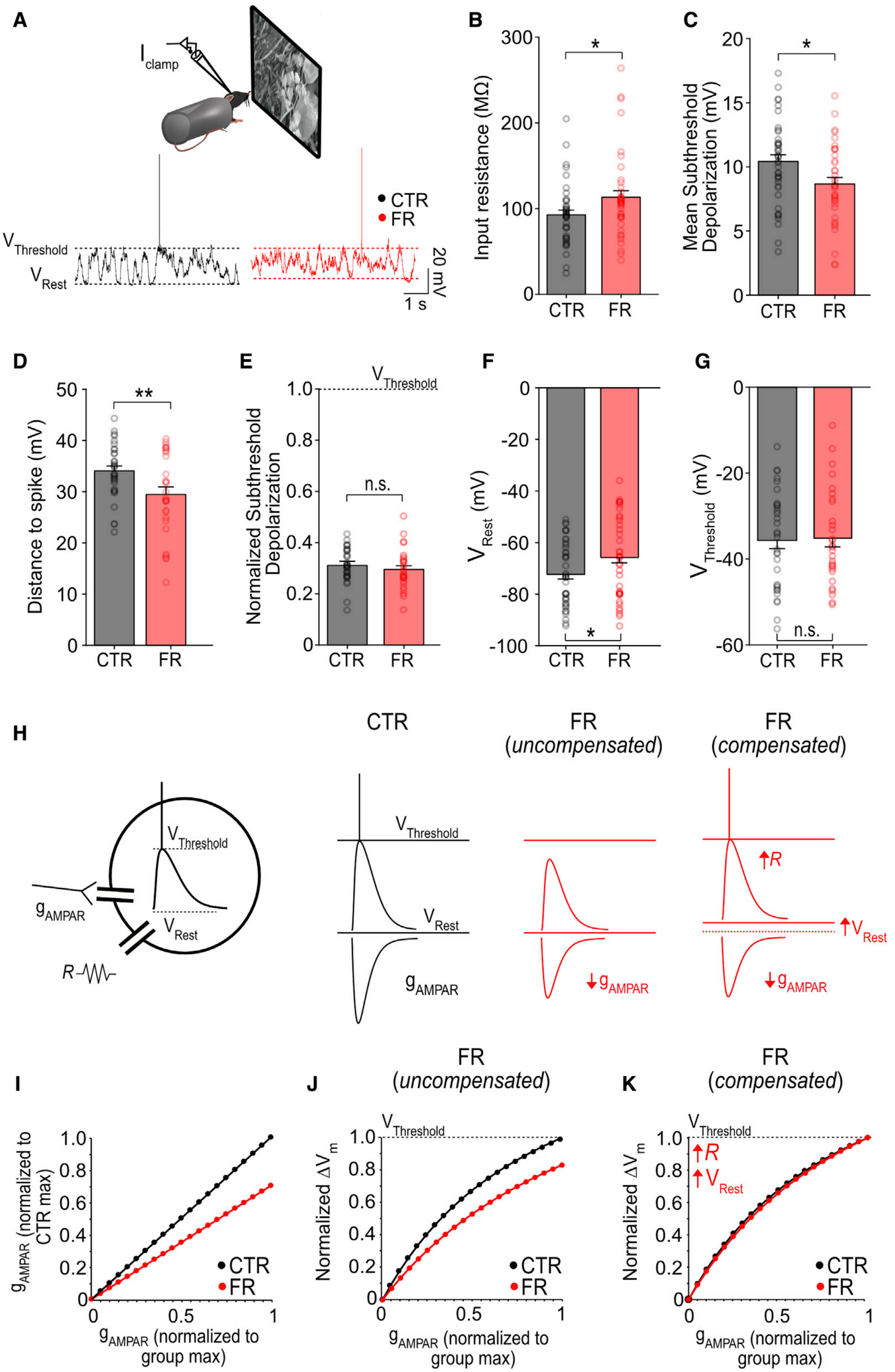

(legend on next page)

subthreshold variability observed with food restriction was not likely to be inherited from presynaptic input (Figures S4E and S4F) but rather emerged from a cell-intrinsic source. Moreover, group differences in subthreshold variability were voltage-dependent. Indeed, modest hyperpolarization of neurons (by 50–100 pA) to prevent spiking abolished group differences in subthreshold variability; of note, the proportion of voltage-dependent subthreshold variability constituted a small fraction of total variability (15% and 28% in control and food-restricted neurons) (Figures 4J and 4K). Collectively, these findings suggest that an intrinsic, voltage-dependent mechanism was necessary for driving increases in subthreshold variability associated with food restriction.

We next examined if the increased subthreshold variability observed with food restriction was sufficient to account for broader orientation tuning. Using the experimentally recorded mean and trial-to-trial variability of drifting grating-evoked subthreshold depolarizations, we calculated the likelihood that a given depolarization would cross spike threshold on the basis of a simple Gaussian noise model in order to infer spike output (Figure 4L). Given that the relative subthreshold responses in both the control and food-restricted models were identical (adopted from Figure 4E), the models only differed in their trial-to-trial subthreshold variability (adopted from Figure 4K). We found that elevated subthreshold variability in the food-restricted model was sufficient to drive a broadening of the orientation tuning of predicted spike output by 36%, comparable with our experimental findings (Figure 4L; compare with Figure 4B). Collectively, our findings show that food restriction increases a cell-intrinsic source of subthreshold variability, which results in broader orientation tuning of spike output by increasing the probability that small depolarizations cross spike threshold.

Increased input resistance and depolarization of the resting membrane potential amplify subthreshold variability and lead to broader orientation tuning

What cell-intrinsic mechanisms underlie the increased subthreshold variability recorded under food restriction? Given the voltage dependence of the increased subthreshold variability, changes in AMPAR conductance with food restriction are unlikely to contribute. Indeed, the subthreshold variability of AMPAR-mediated currents *in vivo* (Figures S4E and S4F) and *ex vivo* (Figure 2C) were unchanged with food restriction. This suggests that non-AMPAR sources, for example, voltage-gated channels, are instead involved. On the basis of Ohm's law, the subthreshold variability associated with these sources should be amplified by the increase in input resistance observed with food restriction. Moreover, the depolarization of the resting membrane potential observed with food restriction, by reducing the distance to spike threshold, should further increase the impact of this variability on spike output. In this way, increased input resistance and depolarization of the resting membrane potential could lead to amplified subthreshold variability with food restriction.

To test this, we used a Hodgkin-Huxley type model neuron (Figure 5A; Tables S1 and S2), which received orientation-tuned synaptic input (gAMPAR) concurrent with variable input from non-AMPARs. This variable input was the only source of trial-to-trial variability. We found that increasing the input resistance or depolarizing the resting membrane potential, while reducing the amplitude of AMPAR conductance in accordance with experimental data, was sufficient to increase the trial-to-trial variability of the subthreshold membrane potential as measured by a 16% and 39% increase in the CV, respectively (Figures 5B and 5C). Combining both an increase in input resistance and depolarization of the resting membrane potential to simulate food-restricted conditions resulted in a 62% increase in subthreshold variability (Figures 5B and 5C). As observed *in vivo*, the elevated subthreshold variability resulted in a leftward shift of the neuronal input/output curve, effectively increasing the probability that smaller depolarizations would trigger spiking (Figure 5D). This led to broader orientation tuning of spike output; the effect was greatest when both input resistance was increased and the resting membrane potential was depolarized (Figure 5F). Collectively, these findings suggest that given some non-AMPAR source of subthreshold variability, increasing input resistance and depolarizing the resting membrane potential to experimentally recorded levels is sufficient to elevate levels of subthreshold variability and broaden orientation tuning.

What might be a biologically plausible candidate for a non-AMPAR source of variability? Given the voltage dependence of

Figure 3. Reduced AMPAR currents are compensated by an increased input resistance and a depolarized resting membrane potential in food-restricted mice

(A) Schema of current-clamp recording of layer 2/3 neurons in awake mice and sample traces ($V_{Threshold}$, spike threshold; V_{Rest}, resting membrane potential).

(B) Mean input resistance (t test; n = 40 control and n = 37 food-restricted cells).

(C) Mean subthreshold depolarization, calculated as the integrated subthreshold membrane potential per second (t test; n = 40 control and n = 37 food-restricted cells).

(D) Mean distance to spike (spike threshold − resting membrane potential) (t test; n = 32 control and n = 26 food-restricted cells that spiked).

(E) Normalized subthreshold depolarization (mean subthreshold depolarization/distance to spike) ($p = 0.8$, t test; n = 32 control and n = 26 food-restricted cells that spiked).

(F) Mean resting membrane potential (t test; n = 40 control and n = 37 food-restricted cells).

(G) Mean spike threshold ($p = 0.41$, t test; n = 32 control and n = 25 food-restricted cells that spiked).

(H) Left: schema of integrate-and-fire model (gAMPAR, synaptic conductance; R, input resistance). Right: sample simulated traces. food-restricted model has 70% of the gAMPAR of control, as found with experimental recording. Spiking activity is restored following compensatory increases in input resistance (R) and depolarization of the resting membrane potential (V_{rest}) (food-restricted uncompensated model versus food-restricted compensated model).

(I) gAMPAR conductance used in the model.

(J) Normalized depolarization triggered by gAMPAR input as shown in (I), for food-restricted uncompensated versus control.

(K) Normalized depolarization triggered by gAMPAR input as shown in (I) for food-restricted compensated versus control.

*$p < 0.05$ and **$p < 0.01$. Error bars are S.E.M. See also Figure S3.

CellPress
OPEN ACCESS

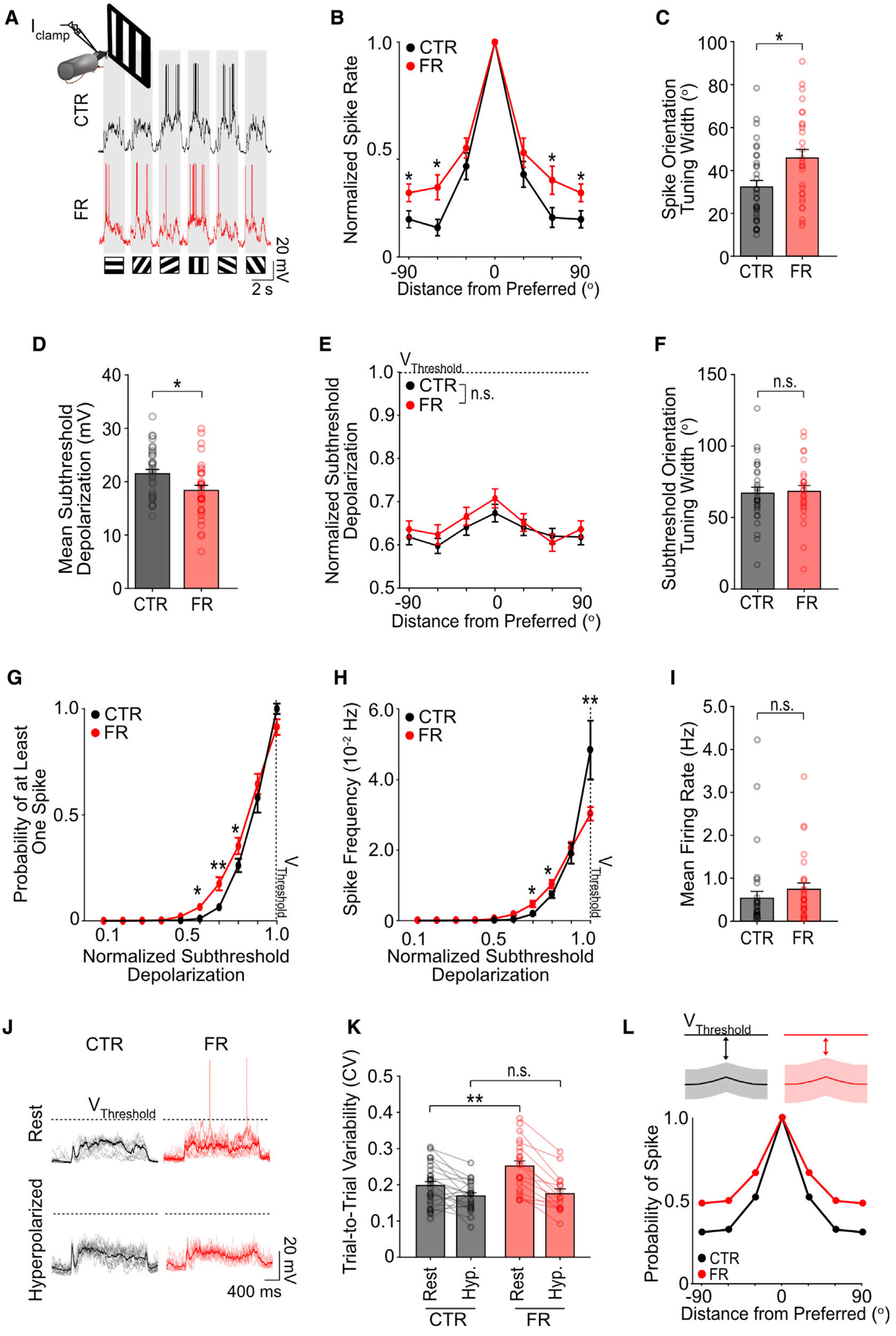

(legend on next page)

the variability, one possibility is the stochastic opening and closing of voltage-dependent channels, which can contribute to biophysical noise (Faisal et al., 2008; Silver, 2010). To examine this possibility, we replaced the variable, non-AMPAR input in our model with a stochastic voltage-gated channel; all other channels remained deterministic (Figure 5G). As before, we found that increasing the input resistance and depolarizing the resting membrane potential increased subthreshold variability, resulting in a leftward shift of the input/output curve and a broadening of orientation tuning (Figures 5H–5L). Thus, increasing the input resistance and depolarizing the resting membrane potential is sufficient to amplify subthreshold variability generated by stochastic voltage-gated channels and contribute to broader orientation tuning of spike output.

Food restriction results in a decrease in cortical coding precision and impaired fine visual discrimination

We next examined to what extent the broadened orientation tuning observed with food restriction affected population coding precision. We used two-photon calcium imaging to monitor the responses of populations of excitatory (CaMKII-positive), GCaMP6s-expressing layer 2/3 neurons in the visual cortex of awake mice. Consistent with electrophysiological recordings, during the presentation of drifting gratings, we found that neurons had a broader orientation tuning in food-restricted mice (Figures 6A–6C; n = 7 control and n = 8 food-restricted animals; Figures S6C–S6E); the proportion of grating-responsive neurons was unchanged across groups (control versus food-restricted animals, 0.71 ± 0.06 versus 0.81 ± 0.02; $p = 0.12$, t test). To examine coding precision of natural stimuli, we imaged layer 2/3 neurons during the presentation of natural scenes and used a maximum likelihood decoder (Montijn et al., 2014) to decode scenes from recorded neuronal activity. Decoding performance reflects how well neuronal population activity in response to distinct visual stimuli can be discriminated. We found that scenes from very different environments (outdoor versus home cage) could be decoded equally well in both control and food-restricted groups; however, decoding of similar scenes taken from the same environment was significantly impaired in the food-restricted group (Figure 6D; n = 6 control and n = 7 FR animals). These findings indicate that under food restriction, population coding of visual information was largely preserved, but coding precision was reduced.

To test whether reduced coding precision affected visual discrimination behaviorally, we designed a water maze task in which animals learned to discriminate a target grating from a non-target grating in order to find a hidden platform and escape the water (Figure 6E; n = 13 control and n = 15 food-restricted animals). Both groups learned the task equally well (Figures S5A–S5D). During testing, the discrimination difficulty was increased by changing the angle of the non-target grating to be progressively more similar to the target grating. With increased difficulty, performance dropped off for both groups; however, compared with control animals, food-restricted animals had a selective deficit with angle differences < 10° (Figure 6E); this finding was consistent with model-based predictions of visual discriminability from calcium imaging data presented in Figure 6 (Figures S5E and S5F). Collectively, these findings indicate that food restriction did not affect gross visual perception but selectively impaired fine visual discrimination.

Reduction in visual coding precision under food restriction is associated with reduced levels of serum leptin hormone and is restored by exogenous leptin supplementation

Finally, we hypothesized that signals modulated by metabolic state underlie the changes in cortical function observed under

Figure 4. Increased subthreshold variability contributes to broadened orientation tuning in food-restricted mice
(A) Sample current-clamp recordings of layer 2/3 neurons in awake mice during presentation of drifting gratings.
(B) Mean orientation tuning curve for spike output normalized to the response to the preferred orientation. Note that -90° and +90° conditions correspond to the same visual stimulus (two-way repeated-measures ANOVA, control group versus food-restricted group: $p < 0.001$; post hoc Sidak’s tests; n = 33 control and n = 28 food-restricted cells).
(C) Average orientation tuning width of spike output (t test).
(D) Mean subthreshold response across orientations (t test).
(E) Mean orientation tuning curve for subthreshold depolarization normalized by the distance to spike ($V_{Threshold}$, spike threshold) (two-way repeated-measures ANOVA, control group versus food-restricted group: $p = 0.33$).
(F) Average orientation tuning width for subthreshold depolarization ($p = 0.82$, t test).
(G) Probability of detecting at least one spike as a function of the subthreshold depolarization, normalized to the distance to spike threshold (two-way repeated-measures ANOVA, control group versus food-restricted group: $p < 0.05$; post hoc Sidak’s tests).
(H) Average spike frequency as a function of subthreshold depolarization, normalized to the distance to spike threshold (two-way repeated-measures ANOVA, control group versus food-restricted group: $p < 0.05$; post hoc Sidak’s tests).
(I) Mean firing rate, averaged across orientations ($p = 0.15$, t test).
(J) Sample traces depicting trial-to-trial variability (faded traces) and mean (bold traces) of stimulus-evoked depolarizations. Traces were recorded with no holding current (Rest) or hyperpolarized by 50–100 pA to prevent spiking (Hyp.).
(K) Mean coefficient of subthreshold trial-to-trial variability (CV; SD/mean) (two-way ANOVA, Rest versus Hyp.: $p < 0.03$; post hoc Sidak’s tests; control Rest versus food-restricted Rest, $p < 0.01$; n = 28 control and n = 24 food-restricted cells; control Hyp. versus food-restricted Hyp., $p = 0.69$; n = 22 control and n = 16 food-restricted cells; lines connect measures from the same cell).
(L) Top: schematic of model used to calculate the probability that membrane potential would cross spike threshold for a given orientation depending on (1) the distance to spike threshold and (2) the trial-to-trial variability (shaded region). For comparison, the schema depicts relative membrane potential and trial-to-trial variability, both normalized to the distance to spike threshold for the control and food-restrcited model. Bottom: probability of spiking calculated for each orientation, normalized to the value at the preferred stimulus. The model recapitulates the experimentally obtained orientation tuning curve of spike rate shown in (B).
*$p < 0.05$ and **$p < 0.01$. n = 33 control and n = 28 food-restricted cells unless otherwise specified. Error bars are S.E.M. See also Figure S4.

CellPress
OPEN ACCESS

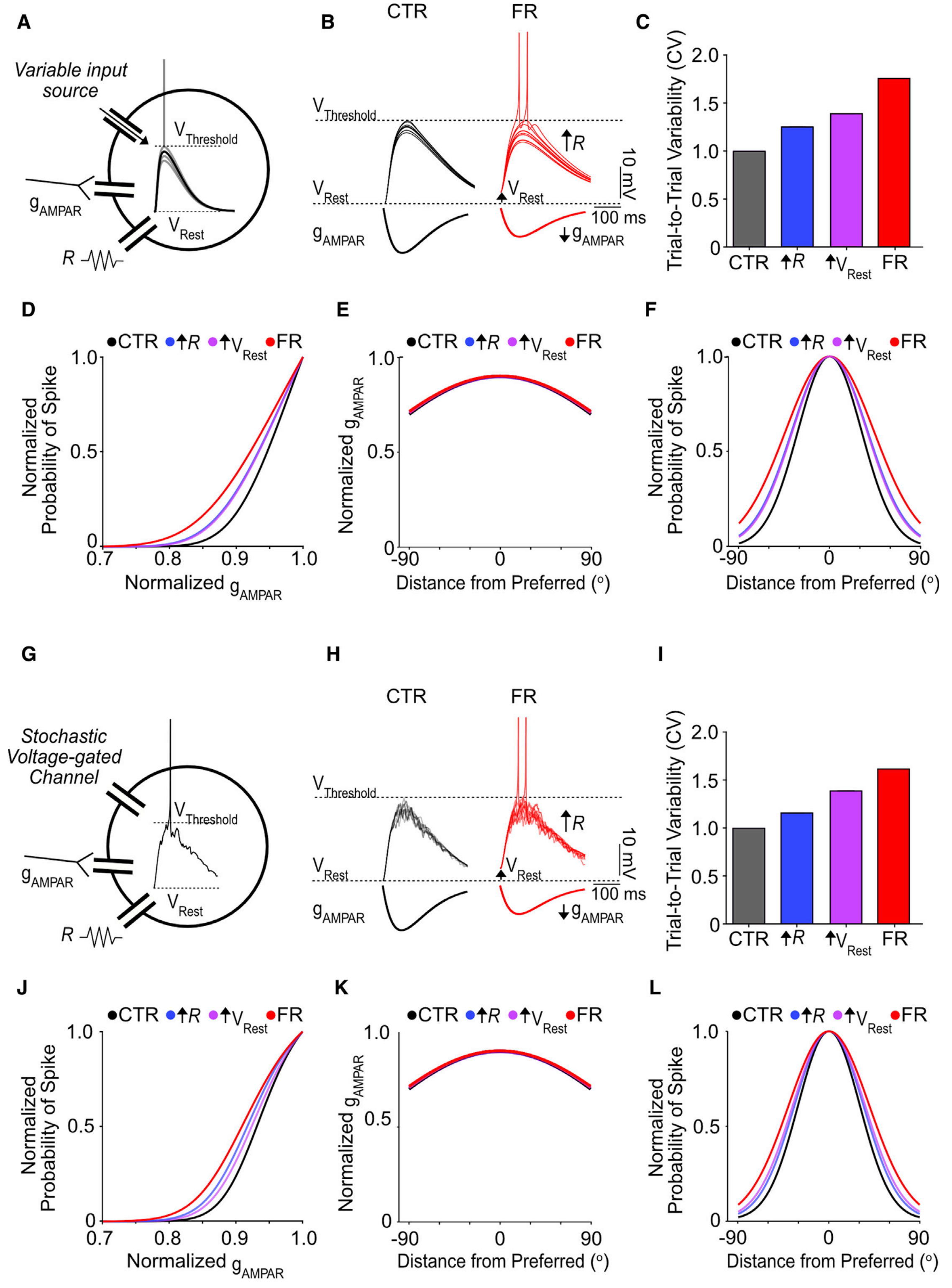

Figure 5. Increased input resistance and depolarization of the resting membrane potential amplifies subthreshold variability, leading to broadened orientation tuning

(A) Hodgkin-Huxley type model neuron with resistance R, resting membrane potential V_{Rest}, and spike threshold $V_{Threshold}$. The model received orientation-tuned synaptic input (gAMPAR), concurrent with input from a variable conductance source (mediated by non-AMPARs), which was the only source of trial-to-trial variability.

(legend continued on next page)

food restriction. We first considered metabolic markers that are commonly modulated by short-term satiety and hunger (glucose, ketone bodies, adrenaline, and corticosterone; Figures S7A–S7E). We examined their serum levels in food-restricted animals before (unsated) and shortly after feeding (<3 h; sated) and in control animals that had *ad libitum* access to food. We found that the levels of these metabolic markers differed between unsated and sated conditions in food-restricted animals but that levels found in sated, food-restricted animals did not significantly differ from controls (Figures S7A–S7E). By contrast, orientation tuning was not affected by short-term satiety and remained broader than controls in both unsated and sated food-restricted animals (Figures S6C–S6E). Thus, metabolic signals associated with short-term satiety and hunger did not underlie the observed changes in coding precision under food restriction.

We found that orientation tuning in food-restricted mice was restored after *ad libitum* feeding was resumed for several days, and animals had recovered their body weight to control levels (Figures S6A and S6F–S6H). We therefore examined metabolic signals linked to long-term satiety and energy balance, focusing on leptin, a hormone secreted by adipocytes in proportion to fat mass (Figure 7A) (Baile et al., 2000). We found that serum leptin, similar to coding precision, was significantly reduced in food-restricted animals (Figure 7B), irrespective of recent feeding (unsated versus sated; Figure S7F). Moreover, restoring leptin levels in food-restricted animals to control levels by exogenous supplementation for 10 days (Figures 7A and 7B) restored orientation tuning and natural scene decoding performance to control values (Figures 7C and 7D). These findings show that a reduction in leptin levels under food restriction is necessary for decreased coding precision in visual cortex.

DISCUSSION

We investigated the impact of food restriction on information coding and energy use in mouse visual cortex (V1). Food restriction induced an energy-saving reduction of excitatory postsynaptic currents, mediated by a decrease in single-channel AMPAR conductance, along with a compensatory increase in input resistance and a depolarization of the resting membrane potential, which preserved neuronal excitability. Collectively, these cell-intrinsic changes enabled neurons to spike at similar rates as controls, while spending less ATP on underlying excitatory currents. However, an increase in input resistance and a depolarization of the resting membrane potential also increased subthreshold variability, leading to broadened orientation tuning, decreased coding precision, and impaired fine visual discrimination. These observed deficits in visual coding correlated with a decrease in serum leptin levels, and were restored to control values via exogenous leptin supplementation.

A key finding of our study is that in times of food scarcity, coding precision in the mammalian cortex is reduced, resulting in energy savings. This is consistent with studies in invertebrates and suggests that the reduction of costly neuronal functions under food shortage is an evolutionary conserved feature to favor survival (Kauffman et al., 2010; Longden et al., 2014; Plaçais et al., 2017; Placais and Preat, 2013). It is important to note that the impact of dietary restriction on brain function may depend on several factors, including age, sex, the severity of calorie restriction, as well as the duration and composition of the diet (Dagon et al., 2005; Fu et al., 2017; Ingram and de Cabo, 2017; Mitchell et al., 2016; Yanai et al., 2004). For example, one study reporting enhanced visual cortical plasticity in rats following food restriction used a diet that did not generate weight loss but rather reduced weight gain relative to controls (Spolidoro et al., 2011). Thus, it is possible that more modest forms of food restriction may have a beneficial impact on information processing (Fu et al., 2017; Ingram and de Cabo, 2017; Mitchell et al., 2016). This is consistent with the theory of hormesis, which posits that mild stressors optimize physiological function (Mattson, 2019). Preserving energy may become more important for survival as food becomes increasingly scarce and body weight substantially decreases.

In our study, food restriction leading to 15% body weight loss resulted in a decrease in cortical coding precision. As animals were sated prior to recordings, our experimental findings were not associated with short-term effects of hunger. Previous studies have found that hunger can have a selective impact on the cortical representation of visual stimuli related to food (Burgess et al., 2016, 2018; Livneh et al., 2017). We did not examine coding precision of food-related stimuli in this study. Thus, although our data indicate that cortical information coding is generally downregulated by food restriction, it may be specifically enhanced for food-related stimuli in times of hunger. Given that food restriction is widely used to motivate animals in

(B) Sample simulated membrane potential traces in response to constant gAMPAR input; action potentials are clipped for clarity. The magnitude of gAMPAR was set so that all models achieved the same normalized level of mean depolarization.
(C) Relative trial-to-trial subthreshold variability, measured as the coefficient of variation (CV), normalized to control.
(D) Probability of detecting at least one spike as a function of gAMPAR input; gAMPAR is expressed as a fraction of the maximum control value. R and V_{Rest} models are overlapping.
(E) gAMPAR input across orientations; gAMPAR is expressed as a fraction of the maximum control value. All models are overlapping.
(F) Orientation tuning curve for spike probability, normalized to the value at the preferred orientation. R and V_{Rest} models are overlapping.
(G) Hodgkin-Huxley type model neuron. As in (A), but the variable conductance source is replaced with a stochastic voltage-gated channel to generate subthreshold variability.
(H) Sample simulated membrane potential traces showing trial-to-trial variability in response to constant gAMPAR input; action potentials are clipped for clarity. The magnitude of gAMPAR was set so that all models achieved the same normalized level of mean depolarization.
(I) Relative trial-to-trial subthreshold variability, measured as the coefficient of variation (CV), normalized to control.
(J) Probability of detecting at least one spike as a function of gAMPAR input; gAMPAR input is expressed as a fraction of the maximum control value.
(K) gAMPAR input across orientations; gAMPAR is expressed as a fraction of the maximum control value. All models are overlapping.
(L) Orientation tuning curve for spike probability (normalized to preferred orientation). See also Supplemental Table S1 and S2.

CellPress
OPEN ACCESS

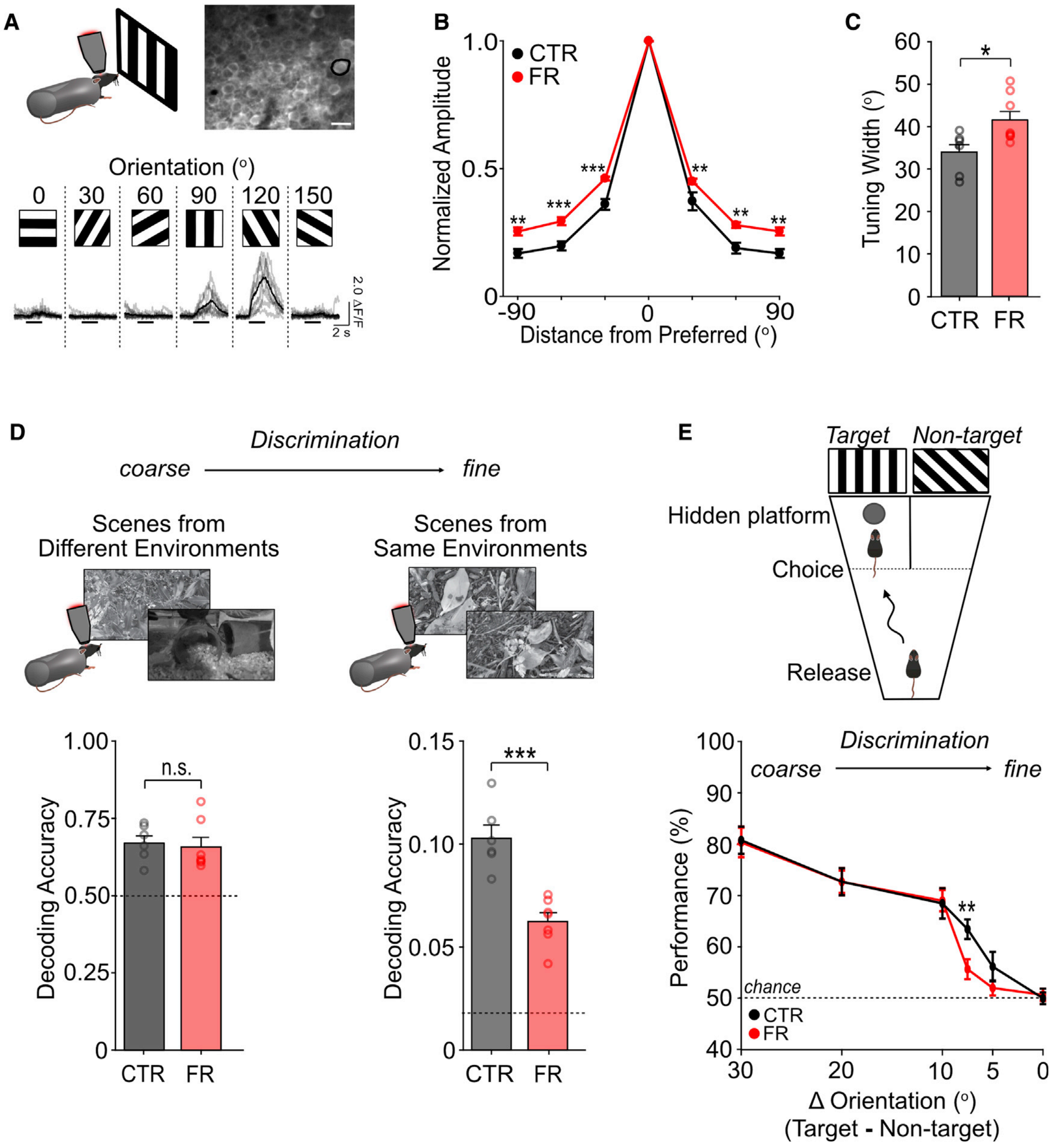

Figure 6. Food restriction results in a decrease in cortical coding precision and impaired fine visual discrimination

(A) Top: example field of view of GCaMP6s-labeled layer 2/3 neurons (scale bar: 10 μm). Bottom: sample fluorescent traces of a neuron (indicated by the broken circle in the above panel) during presentation of six drifting gratings. Individual trials in gray; average trace in black.

(B) Mean orientation tuning normalized to the response to the preferred orientation. Note that -90° and +90° conditions correspond to the same visual stimulus (three-way ANOVA run for the whole dataset shown in Figures S6C and S6F; post hoc Sidak's tests; n = 7 control and n = 8 food-restricted mice, with 478 control and 866 grating-responsive neurons, respectively).

(C) Mean orientation tuning width for control and food-restricted (two-way ANOVA run for the whole dataset shown in Figures S6D and S6G; post hoc Sidak's test; n = 7 control and n = 8 food-restricted mice).

(D) Left: mean accuracy in decoding natural images taken from different environments (coarse discrimination; dissimilarity score between scenes = 41.8; see STAR Methods for calculation of dissimilarity score) (maximum likelihood decoder; p = 0.76, t test; n = 6 control and n = 7 food-restricted mice). Dotted line: chance level (1/2 scenes from different environment). Right: mean accuracy in decoding natural images taken from the same environment (fine discrimination; dissimilarity score between scenes = 22.9) (t test; n = 6 control and n = 7 food-restricted animals). Dotted line: chance level (1/58 scenes from the same environment). The same number of neurons was used for decoding across animals.

(E) Top: schematic for behavioral experiments. Animals were placed in a modified water Y-maze and had to choose the arm associated with a target grating in order to reach a hidden platform and escape the water. Bottom: behavioral performance as a function of discrimination difficulty, which was altered by changing the angle difference between target and non-target grating. Dotted line: chance (*a priori* Sidak's test: control versus food-restricted at 10°, p = 0.85; at 7.5°, p < 0.01; at 5°, p = 0.14; n = 13 control and n = 15 food-restricted mice).

*p < 0.05, **p < 0.01, and ***p < 0.001. Error bars are S,E.M. See also Figure S5.

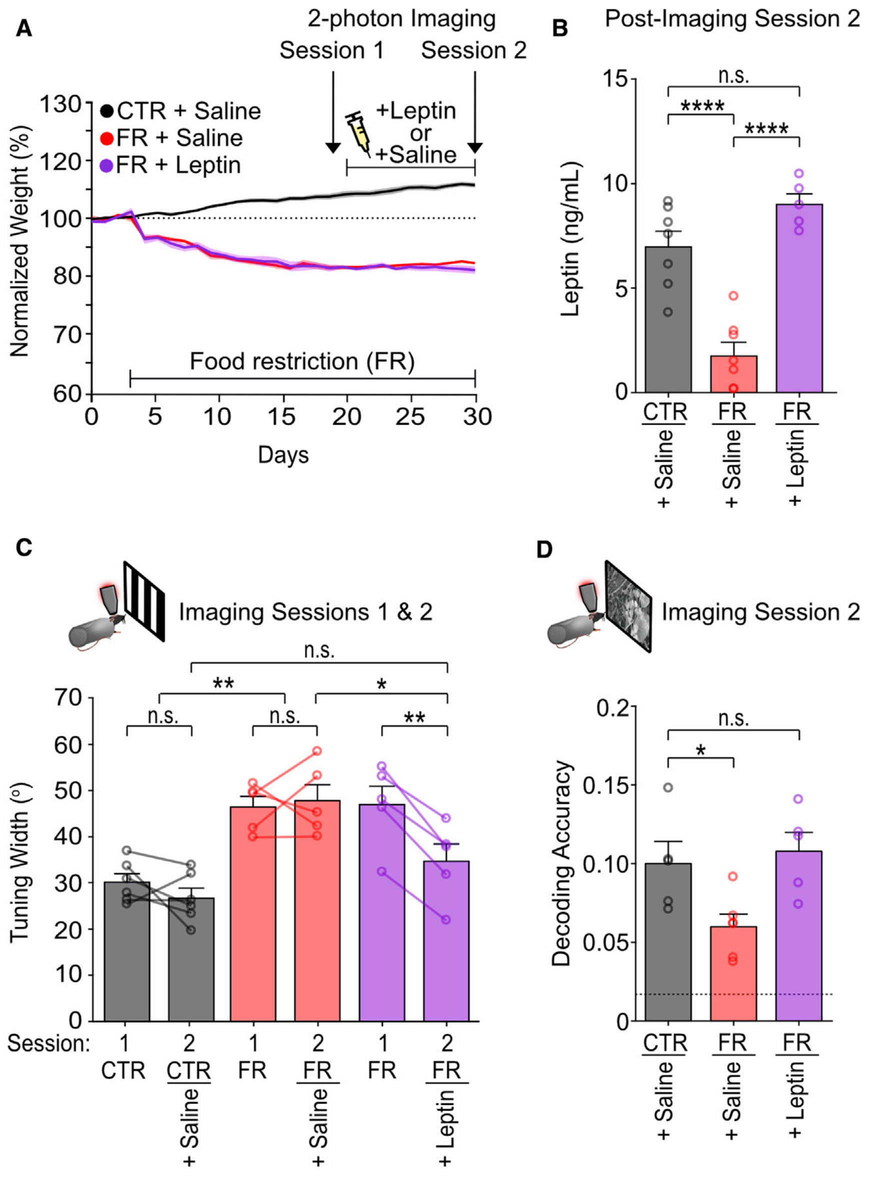

Figure 7. Food restriction affects cortical coding precision via leptin signaling

(A) Experimental timeline and animal weight.

(B) Mean leptin levels (one-way ANOVA, $p < 0.0001$; post hoc Sidak's test, control + saline versus food-restricted + leptin: $p = 0.15$; $n = 7$ CTR + saline, $n = 7$ food-restricted + saline, and $n = 5$ food-restricted + leptin animals).

(C) Mean orientation tuning width (two-way repeated-measures ANOVA; group effect: $p < 0.0005$; post hoc Sidak's test, control versus control + saline: $p = 0.54$; food-restricted versus food-restricted + saline: $p = 0.96$; control versus food-restricted: $p = 0.015$; control saline versus food-restricted saline: $p < 0.0001$; CTR + saline versus food-restricted + leptin: $p = 0.17$; $n = 6$ control + saline, $n = 5$ food-restricted + saline, and $n = 5$ FR + leptin animals).

(D) Mean accuracy in decoding natural images taken from the same environment (fine discrimination) (one-way ANOVA, $p = 0.018$; control + saline versus food-restricted + leptin: $p = 0.87$; $n = 5$ control + saline, $n = 6$ food-restricted + saline, and $n = 5$ food-restricted + leptin animals). Dotted line: chance level (1/59 scenes from the same environment).

*$p < 0.05$, **$p < 0.01$, and ****$p < 0.0001$. Error bars are S,E.M. See also Figures S6 and S7.

behavioral experiments examining cortical function, our findings indicate that the general impact of food restriction on neuronal activity should be taken into consideration in such studies (Goltstein et al., 2018; Toth and Gardiner, 2000). Our results are also relevant for studies using water restriction, since water restiction can lead to decreased food intake and body weight associated with decreased fat mass and leptin levels (Bekkevold et al., 2013; Takei et al., 2012; The International Brain Laboratory, 2021).

We found that the reduction in visual coding precision under food restriction was associated with reduced levels of serum leptin, rather than metabolic signals associated with short-term satiety or hunger, and was restored by exogenous leptin supplementation. These findings highlight the critical role of the adipocyte-secreted hormone leptin in metabolic state-dependent changes in cortical function. How might changes in circulating leptin levels underlie the cortical changes we report with food restriction? One possibility is via direct receptor action, as leptin receptors are expressed throughout the cortex and leptin can cross the blood-brain barrier (Harvey, 2007; Morrison, 2009; Shioda et al., 1998). Moreover, leptin has been shown to alter intrinsic electrophysiological and synaptic properties of neurons *ex vivo* (Harvey, 2007; Morrison, 2009; Shanley et al., 2002). A second possibility is that leptin may exert its impact via other brain areas, most notably the hypothalamus, which contains a high concentration of leptin receptors (Shioda et al., 1998), and can affect cortical activity via direct or indirect neuronal projections or via alterations in neuromodulatory tone (Saper, 2000). A final possibility is via leptin-mediated modulation of other metabolic signals, such as thyroid hormones, which are also known to modulate neuronal electrophysiology (Ahima et al., 1996; Hoffmann and Dietzel, 2004).

Our results show that reduced coding precision was accompanied by a 29% reduction in energy consumption associated with excitatory currents in excitatory layer 2/3 neurons in visual cortex. Our analysis focused on energy use related to excitatory postsynaptic currents and action potentials, which collectively account for approximately 80% of the energy budget for electrical signaling in gray matter (Harris et al., 2012; Sengupta et al., 2010). However, the brain's full energy budget includes other signaling activities, such as presynaptic vesicle release, as well as non-signaling activities, such as cellular housekeeping and repair (Engl and Attwell, 2015; Harris et al., 2012; Rangaraju

et al., 2014). Further work is therefore required to understand the full impact of food restriction on total brain energy expenditure and information processing, including the study of other cell types (e.g., inhibitory interneurons, astrocytes, and oligodendrocytes) and other brain regions.

Mechanistically, we found that energy savings were mediated by a decrease in single-channel AMPAR conductance. The resulting reduction in current flux through these receptors would be associated with less ATP spent by the Na^+/K^+ ATPase in re-establishing ion gradients. A reduction in single-channel conductance could be caused by a change in subunit composition or altered phosphorylation state of receptors or their auxiliary proteins (Greger et al., 2017). Although there are other means of reducing AMPAR currents, such as reducing AMPAR channel number or open channel probability, we did not find evidence of these in our study. The binomial theorem predicts that reducing such parameters would negatively affect the signal-to-noise ratio of synaptic transmission, whereas this would not be the case with reducing single-channel conductance (Harris et al., 2012).

With food restriction, decreased AMPAR conductance was associated with compensatory increases in input resistance and a depolarization of resting membrane potential. These compensatory changes effectively normalized synaptic depolarization to control levels, enabling neurons to maintain comparable firing rates as controls, but with reduced energy expenditure. These compensatory changes, however, had a cost to coding precision, as they amplified the impact of subthreshold variability on action potential output. As a consequence, we found that smaller depolarizations had an increased chance of crossing spike threshold, ultimately leading to broadening of orientation tuning. Although the spike rate triggered by small depolarization was increased, the overall spike rate was not significantly changed, because of a decrease in the spike rate triggered by larger depolarizations. Why was spiking rate reduced instead of elevated at larger depolarizations? For larger depolarizations, subthreshold variability has less positive impact on spiking and may instead reduce spike rate by reducing the duration with which large depolarizations stay above threshold (Destexhe et al., 2001; Hô and Destexhe, 2000). Alternatively, it is possible that other cell-intrinsic changes induced by food restriction curtail spiking during large depolarizations, for instance, via the action of ATP-gated K^+ channels (Lutas and Yellen, 2013; Ma et al., 2007). Regardless the mechanism, our findings indicate that there is a strong drive to maintain spike rate homeostasis *in vivo* (Barnes et al., 2015; Hengen et al., 2013; Keck et al., 2013, 2017; Torrado Pacheco et al., 2021; Wu et al., 2020), even when energy intake is reduced.

Our study focused on how broadened orientation tuning and increased subthreshold variability with food restriction were driven by cell-intrinsic mechanisms. Both changes, in principle, could have instead been driven by changes in presynaptic input. However, we did not find evidence in support of this. First, we found a concurrent broadening of excitatory and inhibitory inputs into layer 2/3 neurons of food-restricted mice, such that the resulting tuning curve of subthreshold depolarization was not different between control and food-restricted groups. This suggests that broadened orientation tuning of spike output was not directly inherited by presynaptic input. We would hypothesize that the broadened tuning of presynaptic excitatory input could be driven by the same cell-intrinsic changes described in this study; a proportional broadening of inhibitory input may then emerge consequentially, given that inhibitory interneurons receive inputs from local excitatory neurons (Hofer et al., 2011). Second, we found no evidence that presynaptic input drove the increase in subthreshold variability with food restriction. Although presynaptic input is responsible for driving a large source of subthreshold variability *in vivo* (Arieli et al., 1996; Destexhe et al., 2001), we found that a small fraction (15%) of subthreshold variability was not readily attributable to presynaptic input but was instead cell-intrinsic and voltage-dependent. One potential candidate for this source is the stochastic opening and closing of voltage-gated channels, which is known to contribute to biophysical noise (Faisal et al., 2008; Silver, 2010). Regardless the source, it was the voltage-dependent component of variability that we found to be amplified with food restriction. Future work is required to explore the network-level consequences of the broadened orientation tuning and cell-intrinsic changes described in this study.

In conclusion, our results show that information coding and energy use can be regulated via cell-intrinsic mechanisms that determine the degree to which energy is spent minimizing the impact of subthreshold variability on information coding. In this way, the brain is able to dynamically adapt its coding precision and energy expenditure in a context-dependent manner to benefit survival.

STAR★METHODS

Detailed methods are provided in the online version of this paper and include the following:

- KEY RESOURCES TABLE
- RESOURCE AVAILABILITY
  - Lead contact
  - Materials availability
  - Data and code availability
- EXPERIMENTAL MODEL AND SUBJECT DETAILS
- METHOD DETAILS
  - AAV injection and cranial window
  - Habituation and head-fixation
  - Pupil and movements tracking
  - *In vivo* two-photon imaging
  - Visual stimuli
  - *In vivo* ATP imaging
  - *In vivo* electrophysiology
  - *Ex vivo* electrophysiology
  - Forced-choice visual discrimination task for assessing visual discrimination
  - Leptin supplementation
  - Serum collection and analysis
  - Hodgkin-Huxley type model neuron
  - Model parameters
  - Synaptic input
  - External and intrinsic noise
  - Simulations and analysis
  - Integrate-and-fire model neuron
- QUANTIFICATION AND STATISTICAL ANALYSIS
  - Pupil analysis
  - Ca^{2+} Imaging Analysis

- ○ Analysis of neuronal responses to drifting gratings
- ○ Grating-responsive neurons
- ○ Natural stimuli-responsive neurons
- ○ Decoding
- ○ Natural stimuli
- ○ ATP measurements
- ○ ATP usage estimation from electrophysiological recordings
- ○ Analysis of patch-clamp electrophysiological recordings
- ○ *In vivo* input-output curves
- ○ *Ex vivo* EPSC measures
- ○ *Ex vivo* mEPSC analysis
- ○ *Ex vivo* mean variance analysis
- ○ Behavior
- ○ Gaussian noise model of orientation tuning
- ○ Statistics

SUPPLEMENTAL INFORMATION

Supplemental information can be found online at https://doi.org/10.1016/j.neuron.2021.10.024.

ACKNOWLEDGMENTS

We thank the GENIE Program and the Janelia Research Campus, specifically V. Jayaraman, R. Kerr, D. Kim, L. Looger, and K. Svoboda, for making GCaMP6 available. We thank Johannes Hirrlinger for providing the ATeam1.03^{YEMK} mouse line. We thank Ian Duguid and his group members for critical advice on *in vivo* patch-clamp recordings and for feedback on the manuscript, Peter Kind for giving us access to equipment for acute slice recordings, and Emily Osterweil for giving us access to a plate reader. We thank Mark Van Rossum for advice on the computational models, Emma Wood for advice on the behavioral task, and Raphael Gould for his help with writing MATLAB scripts to analyze *ex vivo* electrophysiology data. This work was funded by the Biotechnology and Biological Sciences Research Council (BBSRC) (Responsive Mode Research Grant [BB/T007907/1] to N.L.R. and Z.P.), the Royal Commission of 1851 (research fellowship to Z.P.), the Wellcome Trust and the Royal Society (Sir Henry Dale fellowship to N.L.R.), the Shirley Foundation, the Patrick Wild Centre, the RS MacDonald Charitable Trust Seedcorn Grant (to N.L.R.), and the Simons Initiative for the Developing Brain (to N.L.R.). This project has received funding from the European Research Council (ERC) under the European Union's Horizon 2020 research and innovation program (grant agreement 866386).

AUTHOR CONTRIBUTIONS

Z.P. initiated the project. Z.P. and N.L.R. designed the experiments. Z.P. performed and analyzed calcium imaging and electrophysiological recordings. D.K. performed ATP imaging and analyzed the data. D.K. designed and performed behavioral experiments and analyzed the data. N.D. designed and implemented the computational models. N.L.R. supervised the project. Z.P. and N.L.R. wrote the manuscript with input from all authors.

DECLARATION OF INTERESTS

The authors declare no competing interests

INCLUSION AND DIVERSITY

One or more of the authors of this paper self-identifies as an underrepresented ethnic minority in science. One or more of the authors of this paper self-identifies as a member of the LGBTQ+ community. While citing references scientifically relevant for this work, we also actively worked to promote gender balance in our reference list.

Received: June 3, 2021
Revised: September 7, 2021
Accepted: October 15, 2021
Published: November 5, 2021

REFERENCES

Adesnik, H. (2017). Synaptic mechanisms of feature coding in the visual cortex of awake mice. Neuron *95*, 1147–1159.e4.

Ahima, R.S., Prabakaran, D., Mantzoros, C., Qu, D., Lowell, B., Maratos-Flier, E., and Flier, J.S. (1996). Role of leptin in the neuroendocrine response to fasting. Nature *382*, 250–252.

Albert, M.V., Schnabel, A., and Field, D.J. (2008). Innate visual learning through spontaneous activity patterns. PLoS Comput. Biol. *4*, e1000137.

Arieli, A., Sterkin, A., Grinvald, A., and Aertsen, A. (1996). Dynamics of ongoing activity: explanation of the large variability in evoked cortical responses. Science *273*, 1868–1871.

Attwell, D., and Laughlin, S.B. (2001). An energy budget for signaling in the grey matter of the brain. J. Cereb. Blood Flow Metab. *21*, 1133–1145.

Azouz, R., and Gray, C.M. (1999). Cellular mechanisms contributing to response variability of cortical neurons in vivo. J. Neurosci. *19*, 2209–2223.

Baeza-Lehnert, F., Saab, A.S., Gutiérrez, R., Larenas, V., Díaz, E., Horn, M., Vargas, M., Hösli, L., Stobart, J., Hirrlinger, J., et al. (2019). Non-canonical control of neuronal energy status by the Na^+ pump. Cell Metab. *29*, 668–680.e4.

Baile, C.A., Della-Fera, M.A., and Martin, R.J. (2000). Regulation of metabolism and body fat mass by leptin. Annu. Rev. Nutr. *20*, 105–127.

Barlow, H.B. (2012). Possible principles underlying the transformations of sensory messages. In Sensory Communication (Cambridge, MA: MIT Press), pp. 216–234.

Barnes, S.J., Sammons, R.P., Jacobsen, R.I., Mackie, J., Keller, G.B., and Keck, T. (2015). Subnetwork-specific homeostatic plasticity in mouse visual cortex in vivo. Neuron *86*, 1290–1303.

Bekkevold, Christine, Robertson, Kimberly, L, Reinhard, Mary, K, Battles, August, H, and Rowland, Neil, E (2013). Dehydration parameters and standards for laboratory mice. J Am Assoc Lab Anim Sci . *52*, 233–239.

Brown, A.P.Y., Cossell, L., and Margrie, T.W. (2019). Visual experience regulates the intrinsic excitability of visual cortical neurons to maintain sensory function. Cell Rep. *27*, 685–689.e4.

Burgess, C.R., Ramesh, R.N., Sugden, A.U., Levandowski, K.M., Minnig, M.A., Fenselau, H., Lowell, B.B., and Andermann, M.L. (2016). Hunger-dependent enhancement of food cue responses in mouse postrhinal cortex and lateral amygdala. Neuron *91*, 1154–1169.

Burgess, C.R., Livneh, Y., Ramesh, R.N., and Andermann, M.L. (2018). Gating of visual processing by physiological need. Curr. Opin. Neurobiol. *49*, 16–23.

Carter, B.C., Giessel, A.J., Sabatini, B.L., and Bean, B.P. (2012). Transient sodium current at subthreshold voltages: activation by EPSP waveforms. Neuron *75*, 1081–1093.

Chance, F.S., Abbott, L.F., and Reyes, A.D. (2002). Gain modulation from background synaptic input. Neuron *35*, 773–782.

Dagon, Y., Avraham, Y., Magen, I., Gertler, A., Ben-Hur, T., and Berry, E.M. (2005). Nutritional status, cognition, and survival: a new role for leptin and AMP kinase. J. Biol. Chem. *280*, 42142–42148.

Deneux, T., Kaszas, A., Szalay, G., Katona, G., Lakner, T., Grinvald, A., Rózsa, B., and Vanzetta, I. (2016). Accurate spike estimation from noisy calcium signals for ultrafast three-dimensional imaging of large neuronal populations in vivo. Nat. Commun. *7*, 12190.

Destexhe, A., Rudolph, M., Fellous, J.-M., and Sejnowski, T.J. (2001). Fluctuating synaptic conductances recreate in vivo-like activity in neocortical neurons. Neuroscience *107*, 13–24.

Engl, E., and Attwell, D. (2015). Non-signalling energy use in the brain. J. Physiol. *593*, 3417–3429.

Faisal, A.A., Selen, L.P.J., and Wolpert, D.M. (2008). Noise in the nervous system. Nat. Rev. Neurosci. *9*, 292–303.

CellPress
OPEN ACCESS

Fu, Y., Chen, Y., Li, L., Wang, Y., Kong, X., and Wang, J. (2017). Food restriction affects Y-maze spatial recognition memory in developing mice. Int. J. Dev. Neurosci. *60*, 8–15.

Gentet, L.J., Stuart, G.J., and Clements, J.D. (2000). Direct measurement of specific membrane capacitance in neurons. Biophys. J. *79*, 314–320.

Golomb, D., Yue, C., and Yaari, Y. (2006). Contribution of persistent Na+ current and M-type K+ current to somatic bursting in CA1 pyramidal cells: combined experimental and modeling study. J. Neurophysiol. *96*, 1912–1926.

Goltstein, P.M., Reinert, S., Glas, A., Bonhoeffer, T., and Hübener, M. (2018). Food and water restriction lead to differential learning behaviors in a head-fixed two-choice visual discrimination task for mice. PLoS ONE *13*, e0204066.

Greger, I.H., Watson, J.F., and Cull-Candy, S.G. (2017). Structural and functional architecture of AMPA-type glutamate receptors and their auxiliary proteins. Neuron *94*, 713–730.

Halaas, J.L., Gajiwala, K.S., Maffei, M., Cohen, S.L., Chait, B.T., Rabinowitz, D., Lallone, R.L., Burley, S.K., and Friedman, J.M. (1995). Weight-reducing effects of the plasma protein encoded by the obese gene. Science *269*, 543–546.

Hansel, D., and Sompolinsky, H. (1996). Chaos and synchrony in a model of a hypercolumn in visual cortex. J. Comput. Neurosci. *3*, 7–34.

Harris, J.J., Jolivet, R., and Attwell, D. (2012). Synaptic energy use and supply. Neuron *75*, 762–777.

Harris, J.J., Jolivet, R., Engl, E., and Attwell, D. (2015). Energy-efficient information transfer by visual pathway synapses. Curr. Biol. *25*, 3151–3160.

Harvey, J. (2007). Leptin: a diverse regulator of neuronal function. J. Neurochem. *100*, 307–313.

Hengen, K.B., Lambo, M.E., Van Hooser, S.D., Katz, D.B., and Turrigiano, G.G. (2013). Firing rate homeostasis in visual cortex of freely behaving rodents. Neuron *80*, 335–342.

Henschke, J.U., Dylda, E., Katsanevaki, D., Dupuy, N., Currie, S.P., Amvrosiadis, T., Pakan, J.M.P., and Rochefort, N.L. (2020). Reward association enhances stimulus-specific representations in primary visual cortex. Curr. Biol. *30*, 1866–1880.e5.

Herculano-Houzel, S. (2011). Scaling of brain metabolism with a fixed energy budget per neuron: implications for neuronal activity, plasticity and evolution. PLoS ONE *6*, e17514.

Hines, M.L., and Carnevale, N.T. (1997). The NEURON simulation environment. Neural Comput. *9*, 1179–1209.

Hines, M.L., Davison, A.P., and Muller, E. (2009). NEURON and Python. Front. Neuroinform. *3*, 1.

Hladik, C.M. (1988). Seasonal variations in food supply for wild primates. In Coping with Uncertainty in Food Supply, I. de Garine and G.A. Harrison, eds. (Oxford, UK: Clarendon), pp. 1–25.

Hô, N., and Destexhe, A. (2000). Synaptic background activity enhances the responsiveness of neocortical pyramidal neurons. J. Neurophysiol. *84*, 1488–1496.

Hodgkin, A.L., and Huxley, A.F. (1952). A quantitative description of membrane current and its application to conduction and excitation in nerve. J. Physiol. *117*, 500–544.

Hofer, S.B., Ko, H., Pichler, B., Vogelstein, J., Ros, H., Zeng, H., Lein, E., Lesica, N.A., and Mrsic-Flogel, T.D. (2011). Differential connectivity and response dynamics of excitatory and inhibitory neurons in visual cortex. Nat. Neurosci. *14*, 1045–1052.

Hoffmann, G., and Dietzel, I.D. (2004). Thyroid hormone regulates excitability in central neurons from postnatal rats. Neuroscience *125*, 369–379.

Ingram, D.K., and de Cabo, R. (2017). Calorie restriction in rodents: caveats to consider. Ageing Res. Rev. *39*, 15–28.

Kaifosh, P., Zaremba, J.D., Danielson, N.B., and Losonczy, A. (2014). SIMA: Python software for analysis of dynamic fluorescence imaging data. Front. Neuroinform. *8*, 80.

Kass, R.E., and Raftery, A.E. (1995). Bayes factors. J. Am. Stat. Assoc. *90*, 773–795.

Kauffman, A.L., Ashraf, J.M., Corces-Zimmerman, M.R., Landis, J.N., and Murphy, C.T. (2010). Insulin signaling and dietary restriction differentially influence the decline of learning and memory with age. PLoS Biol. *8*, e1000372.

Keck, T., Keller, G.B., Jacobsen, R.I., Eysel, U.T., Bonhoeffer, T., and Hübener, M. (2013). Synaptic scaling and homeostatic plasticity in the mouse visual cortex in vivo. Neuron *80*, 327–334.

Keck, T., Toyoizumi, T., Chen, L., Doiron, B., Feldman, D.E., Fox, K., Gerstner, W., Haydon, P.G., Hübener, M., Lee, H.K., et al. (2017). Integrating Hebbian and homeostatic plasticity: the current state of the field and future research directions. Philos. Trans. R. Soc. Lond. B Biol. Sci. *372*, 20160158.

Keemink, S.W., Lowe, S.C., Pakan, J.M.P., Dylda, E., van Rossum, M.C.W., and Rochefort, N.L. (2018). FISSA: a neuropil decontamination toolbox for calcium imaging signals. Sci. Rep. *8*, 3493.

Knott, C.D. (1998). Changes in orangutan caloric intake, energy balance, and ketones in response to fluctuating fruit availability. Int. J. Primatol. *19*, 1061–1079.

Lerchundi, R., Huang, N., and Rose, C.R. (2020). Quantitative imaging of changes in astrocytic and neuronal adenosine triphosphate using two different variants of ATeam. Front. Cell. Neurosci. *14*, 80.

Levy, W.B., and Baxter, R.A. (1996). Energy efficient neural codes. Neural Comput. *8*, 531–543.

Liu, B.H., Li, Y.T., Ma, W.P., Pan, C.J., Zhang, L.I., and Tao, H.W. (2011). Broad inhibition sharpens orientation selectivity by expanding input dynamic range in mouse simple cells. Neuron *71*, 542–554.

Livneh, Y., Ramesh, R.N., Burgess, C.R., Levandowski, K.M., Madara, J.C., Fenselau, H., Goldey, G.J., Diaz, V.E., Jikomes, N., Resch, J.M., et al. (2017). Homeostatic circuits selectively gate food cue responses in insular cortex. Nature *546*, 611–616.

Longden, K.D., Muzzu, T., Cook, D.J., Schultz, S.R., and Krapp, H.G. (2014). Nutritional state modulates the neural processing of visual motion. Curr. Biol. *24*, 890–895.

Lutas, A., and Yellen, G. (2013). The ketogenic diet: metabolic influences on brain excitability and epilepsy. Trends Neurosci. *36*, 32–40.

Ma, W., Berg, J., and Yellen, G. (2007). Ketogenic diet metabolites reduce firing in central neurons by opening K(ATP) channels. J. Neurosci. *27*, 3618–3625.

Mattson, M.P. (2019). An evolutionary perspective on why food overconsumption impairs cognition. Trends Cogn. Sci. *23*, 200–212.

Mazurek, M., Kager, M., and Van Hooser, S.D. (2014). Robust quantification of orientation selectivity and direction selectivity. Front. Neural Circuits *8*, 92.

Mitchell, S.J., Madrigal-Matute, J., Scheibye-Knudsen, M., Fang, E., Aon, M., González-Reyes, J.A., Cortassa, S., Kaushik, S., Gonzalez-Freire, M., Patel, B., et al. (2016). Effects of sex, strain, and energy intake on hallmarks of aging in mice. Cell Metab. *23*, 1093–1112.

Montijn, J.S., Vinck, M., and Pennartz, C.M.A. (2014). Population coding in mouse visual cortex: response reliability and dissociability of stimulus tuning and noise correlation. Front. Comput. Neurosci. *8*, 58.

Morrison, C.D. (2009). Leptin signaling in brain: a link between nutrition and cognition? Biochim. Biophys. Acta *1792*, 401–408.

Moujahid, A., D'Anjou, A., and Graña, M. (2014). Energy demands of diverse spiking cells from the neocortex, hippocampus, and thalamus. Front. Comput. Neurosci. *8*, 41.

O'Donnell, C., and van Rossum, M.C.W. (2014). Systematic analysis of the contributions of stochastic voltage gated channels to neuronal noise. Front. Comput. Neurosci. *8*, 105.

Olshausen, B.A., and Field, D.J. (1997). Sparse coding with an overcomplete basis set: a strategy employed by V1? Vision Res. *37*, 3311–3325.

Pakan, J.M., Lowe, S.C., Dylda, E., Keemink, S.W., Currie, S.P., Coutts, C.A., and Rochefort, N.L. (2016). Behavioral-state modulation of inhibition is context-dependent and cell type specific in mouse visual cortex. eLife *5*, 1–18.

Pakan, J.M.P., Currie, S.P., Fischer, L., and Rochefort, N.L. (2018). The Impact of visual cues, reward, and motor feedback on the representation of behaviorally relevant spatial locations in primary visual cortex. Cell Rep. *24*, 2521–2528.

Placais, P.-Y., and Preat, T. (2013). To favor survival under food shortage, the brain disables costly memory. Science *339*, 440–442.

Plaçais, P.-Y., de Tredern, É., Scheunemann, L., Trannoy, S., Goguel, V., Han, K.-A., Isabel, G., and Preat, T. (2017). Upregulated energy metabolism in the Drosophila mushroom body is the trigger for long-term memory. Nat. Commun. *8*, 15510.

Rangaraju, V., Calloway, N., and Ryan, T.A. (2014). Activity-driven local ATP synthesis is required for synaptic function. Cell *156*, 825–835.

Ruxton, G.D., and Beauchamp, G. (2008). Time for some a priori thinking about post hoc testing. Behav. Ecol. *19*, 690–693.

Saper, C.B. (2000). Hypothalamic connections with the cerebral cortex. Prog. Brain Res. *126*, 39–48.

Sengupta, B., Stemmler, M., Laughlin, S.B., and Niven, J.E. (2010). Action potential energy efficiency varies among neuron types in vertebrates and invertebrates. PLoS Comput. Biol. *6*, e1000840.

Shanley, L.J., Irving, A.J., Rae, M.G., Ashford, M.L.J., and Harvey, J. (2002). Leptin inhibits rat hippocampal neurons via activation of large conductance calcium-activated K+ channels. Nat. Neurosci. *5*, 299–300.

Shioda, S., Funahashi, H., Nakajo, S., Yada, T., Maruta, O., and Nakai, Y. (1998). Immunohistochemical localization of leptin receptor in the rat brain. Neurosci. Lett. *243*, 41–44.

Silver, R.A. (2010). Neuronal arithmetic. Nat. Rev. Neurosci. *11*, 474–489.

Spolidoro, M., Baroncelli, L., Putignano, E., Maya-Vetencourt, J.F., Viegi, A., and Maffei, L. (2011). Food restriction enhances visual cortex plasticity in adulthood. Nat. Commun. *2*, 320.

Steinmetz, P.N., Manwani, A., Koch, C., London, M., and Segev, I. (2000). Subthreshold voltage noise due to channel fluctuations in active neuronal membranes. J. Comput. Neurosci. *9*, 133–148.

Sterling, P., and Laughlin, S. (2015). Principles of Neural Design (Cambridge, MA: MIT Press).

Takei, Yoshio, Bartolo, Ray, C, Fujihara, Hiroaki, Ueta, Yoichi, Ueta, Yoichi, and Donald, John, A (2012). Water deprivation induces appetite and alters metabolic strategy in Notomys alexis: unique mechanisms for water production in the desert. Proc Biol Sci . *279*, 2599–2608. https://doi.org/10.1098/rspb.2011.2627.

The International Brain Laboratory. (2021). Standardized and reproducible measurement of decision-making in mice. eLife *10:e63711*. https://doi.org/10.7554/eLife.63711.

Torrado Pacheco, A., Bottorff, J., Gao, Y., and Turrigiano, G.G. (2021). Sleep promotes downward firing rate homeostasis. Neuron *109*, 530–544.e6.

Toth, L.A., and Gardiner, T.W. (2000). Food and water restriction protocols: physiological and behavioral considerations. Contemp. Top. Lab. Anim. Sci. *39*, 9–17.

Traynelis, S.F., Silver, R.A., and Cull-Candy, S.G. (1993). Estimated conductance of glutamate receptor channels activated during EPSCs at the cerebellar mossy fiber-granule cell synapse. Neuron *11*, 279–289.

Trevisiol, A., Saab, A.S., Winkler, U., Marx, G., Imamura, H., Möbius, W., Kusch, K., Nave, K.A., and Hirrlinger, J. (2017). Monitoring ATP dynamics in electrically active white matter tracts. eLife *6*, e24241.

Wang, X.J., and Buzsáki, G. (1996). Gamma oscillation by synaptic inhibition in a hippocampal interneuronal network model. J. Neurosci. *16*, 6402–6413.

Wong, A.A., and Brown, R.E. (2006). Visual detection, pattern discrimination and visual acuity in 14 strains of mice. Genes Brain Behav. *5*, 389–403.

Wu, Y.K., Hengen, K.B., Turrigiano, G.G., and Gjorgjieva, J. (2020). Homeostatic mechanisms regulate distinct aspects of cortical circuit dynamics. Proc. Natl. Acad. Sci. U S A *117*, 24514–24525.

Yamashita, T., Ishikawa, T., and Takahashi, T. (2003). Developmental increase in vesicular glutamate content does not cause saturation of AMPA receptors at the calyx of Held synapse. J. Neurosci. *23*, 3633–3638.

Yanai, S., Okaichi, Y., and Okaichi, H. (2004). Long-term dietary restriction causes negative effects on cognitive functions in rats. Neurobiol. Aging *25*, 325–332.

CellPress
OPEN ACCESS

STAR★METHODS

KEY RESOURCES TABLE

REAGENT or RESOURCE	SOURCE	IDENTIFIER
**Bacterial and virus strains**		
AAV1.Syn.Flex.GCaMP6s.WPRE.SV40	Addgene	RRID:Addgene_100845-AAV1
AAV1.CamKII 0.4.Cre.SV40 AAV1	Addgene	RRID:Addgene_105558-AAV1
**Chemicals, peptides, and recombinant proteins**		
Recombinant Murine Leptin	Peprotech, UK	Cat#AF-450-31
**Critical commercial assays**		
Mouse Leptin Quantikine Elisa Kit	R&D Systems, USA	Cat#MOB00B
**Deposited data**		
Custom MATLAB scripts for data analysis and computational models	This paper	https://github.com/rochefort-lab/Padamsey-et-al-Neuron-2021; https://doi.org/10.5281/zenodo.5561795
**Experimental models: Organisms/strains**		
Mouse: C57BL/6J	The Jackson Laboratory	RRID: IMSR_JAX:000664
Mouse: B6-Tg(Thy1.2-ATeam1.03^{YEMK})^{AJhi}	Prof. Dr. Johannes Hirrlinger, Carl-Ludwig-Institut for Physiology	RRID: MGI:5882597
**Software and algorithms**		
MATLAB 2013/2017a	Mathworks	RRID:SCR_001622
Psychophysics Toolbox package for MATLAB	http://psychtoolbox.org	RRID:SCR_002881
SIMA 1.3.2 (sequential image analysis)	(Kaifosh et al., 2014)	https://pypi.org/project/sima/
FISSA	(Keemink et al., 2018)	https://github.com/rochefort-lab/fissa
ImageJ (Fiji)	NIH – public domain	https://fiji.sc; RRID:SCR_002285
Custom MATLAB scripts for data analysis and computational models	This paper	https://github.com/rochefort-lab/Padamsey-et-al-Neuron-2021 https://doi.org/10.5281/zenodo.5561795
WinWCP	Strathclyde Electrophysiology Software	RRID:SCR_014713
pCLAMP10	Molecular Devices	RRID:SCR_011323
ANY-maze	Stoelting, Europe	RRID:SCR_014289

RESOURCE AVAILABILITY

Lead contact

Further information and requests for resources and materials should be directed to and will be fulfilled by the lead contact Nathalie L. Rochefort (n.rochefort@ed.ac.uk).

Materials availability

This study did not generate new unique reagents.

Data and code availability

- Processed data will be accessible through https://github.com/rochefort-lab/Padamsey-et-al-Neuron-2021. Requests for raw data should be made to and will be fulfilled by the lead contact (n.rochefort@ed.ac.uk).
- Original MATLAB code used for data analysis is available at https://github.com/rochefort-lab/Padamsey-et-al-Neuron-2021.
- Any additional information required to reanalyze the data reported in this paper is available from the lead contact upon request.

EXPERIMENTAL MODEL AND SUBJECT DETAILS

All animal experiments were approved by the Animal Welfare and Ethical Review Board (AWERB) of the University of Edinburgh and were performed under a project license granted by the UK Home Office and conformed with the UK Animals (Scientific Procedures) Act 1986 and the European Directive 86/609/EEC and 2010/63/EU on the protection of animals used for experimental purposes.

This study used male C57BL/6J mice (RRID:IMSR_JAX:000664; Jackson Laboratory) and male B6-Tg (Thy1.2-ATeam1.03^{YEMK})^{AJhi} transgenic mice on a C57BL/6J background (RRID:MGI:5882597; https://scicrunch.org/resources). Animals were group housed (2-5/cage) in a reverse 12h/12h light/dark cycle room that was kept at 21 ± 2°C and 55 ± 10% humidity. Mice 7-9 weeks of age either had *ad libitum* access to food (RM1 expanded pellets for maintenance; DBM Scotland UK) or were food-restricted to 85% of their baseline bodyweight (calculated from a three-day average) for a minimum of 2 weeks prior to experimentation and maintained at this bodyweight for the duration of the experiment (experimental duration: *in vivo* electrophysiology: 1 day; imaging; 1-3 weeks; behavior: 4-8 weeks). Food-restricted animals were given one ration of food 4-8 hours prior to the end of their dark cycle. At the start of food restriction, the food rations started at 4-5g/animal, which was in excess of daily *ad libitum* consumption, and was systematically reduced by at most 20%/day until animals achieved 85% of their baseline bodyweight. Both control and food-restricted animals were handled and weighed daily. Unless otherwise specified, animals were given *ad libitum* access to food for 1-3 hours prior to experimentation in order to state them Food-restricted animals typically required 45-90 minutes to satiate, as evidenced by no observable feeding for 10 minutes, and never consumed more than 80% of their allocated daily food weight during this time. The consumed weight of food was deducted from the day's allocated ration of food, which was given at the regular feeding time. For behavioral experiments, animals were tested after they consumed their daily ration of food.

METHOD DETAILS

AAV injection and cranial window

Mice were anesthetized during surgery using isoflurane and maintained at 37°C using a servo-driven heater. They were given buprenorphine (Vetergesic; 0.1 mg/kg), carprofen (Carprieve, 5 mg/kg), and dexamethasone (Rapidexon, 2 mg/kg) subcutaneously preoperatively, and 25mL/kg of warm Ringer's solution subcutaneously at the end of the surgery. Non-transparent eye cream was applied to protect the eyes (Bepanthen, Bayer, Germany) during surgery. A square craniotomy (2 × 2 mm) was made over the left primary visual cortex with its center at 2.5 mm mediolateral and 0.5 mm anterior to lambda. A flexed variant of GCaMP6S (AAV1.Syn.Flex.GCaMP6s.WPRE.SV40; 1:10 dilution in saline; Addgene; RRID:Addgene_105558-AAV1) along with Cre-recombinase under the CaMKII promoter (AAV1.CamKII 0.4.Cre.SV40; 1:100 dilution in saline; Addgene; RRID:Addgene_105558-AAV1) was injected at the center of the craniotomy. A total of 50 nL of virus solution was injected at each of 3 depths (150, 250 and 350 μm), via a sharp glass pipette, at a rate of 2.5 nL / 30 s using a Nanoject II (Drummond Scientific). Injections started at the deepest site. At least 5 minutes elapsed after the complete injection of 50 nL before moving to another depth. The craniotomy was then covered with a square glass window, which was superglued in place. The window consisted of 2 glass coverslips (Menzel-Glaser # 0) glued together with optically-clear, UV-cured glue (Norland Optical Adhesive no. 60); the inner window was 2.0 mm x 2.0 mm, the outer window was 2.5 mm x 2.5 mm. A custom metal headplate was superglued on to the skull and fixed with dental cement (Paladur, Heraeus Kuzler). Imaging sessions were performed 2-4 weeks after viral injection.

Habituation and head-fixation

Mice were extensively handled (daily for 2-3 weeks) and habituated to the cardboard tube used for the recordings. In addition, mice were exposed to cardboard tubes in their home cage that were similar to the one present on the experimental rig. During imaging or electrophysiological recordings, mice were placed in a similar cardboard tube and head-fixed. The cardboard tube was positioned on top of a Styrofoam wheel (20 cm diameter). For calcium imaging, mice were habituated to head-fixation prior to experimentation in a once daily, 10-15 minute session for 2 days. For ATP imaging and electrophysiological recordings, mice were habituated to the imaging setup without head fixation (10-15 min session/day for 1-2 days). We found that mice quickly became accustomed to the setup, evident by the absence of resistive movements and the presence of occasional grooming behaviors, and remained stationary within the tube (Figure S2B).

Pupil and movements tracking

We used an optical encoder (E7P, 250cpr, Pewatron, Switzerland), to monitor movements of the Styrofoam wheel throughout imaging and recording sessions. Movements associated with the mouse visibly adjusting its posture were readily detected in this way. Pupil dilation was also monitored in some experiments using a camera (30 frames/s; USB 2.0 monochrome camera; ImagingSource).

In vivo two-photon imaging

ATP and calcium imaging were performed using a custom-built resonant scanning two-photon microscope, as described previously (Henschke et al., 2020; Pakan et al., 2016, 2018). The setup was equipped with a Ti:Sapphire laser (Charmeleon Vision-S, Coherent, CA) and GaAsP photomultiplier tubes (Scientifica). Images were acquired using a 25x water-immersion objective (Nikon; CF175 Apo 25XC W; 1.1 NA) at a rate of 40 Hz using a custom-programmed LabView based software (v8.2; National Instruments, UK).

Time-series images of one focal plane per mouse were acquired, imaged at depths between 160 and 280 μm below the pia. For GCaMP6s imaging, excitation was tuned to 920nm.

Visual stimuli

Visual stimuli were generated using MATLAB (Mathworks; RRID:SCR_001622; Psychophysics Toolbox; RRID:SCR_002881) and displayed on an LCD monitor (51 × 29 cm; Dell) at a distance of 20 cm from the eye, contralateral to the hemisphere with the cranial window. For a given trial, 12 drifting gratings with angles ranging from 0 – 330° (30° increments) were presented in random order. Each grating was presented with a temporal frequency of 1 Hz for 2 s. Gratings within a trial had the same spatial frequency. For calcium imaging, gratings across trials were randomly assigned a spatial frequency (0.02, 0.04, 0.16, or 0.32 cpd). For electrophysiological recordings, a spatial frequency of 0.04 cpd was used for all gratings. Gratings were interspersed with the presentation of a gray screen (4 s for imaging and 1 s for electrophysiology experiments). All trials started and ended with the presentation of a black screen (4 s for imaging and 1 s for electrophysiology experiments). For natural stimuli, we either used a movie of the outdoors (filmed at a nearby park) or a movie taken inside the home cage of a group of mice (5 animals) from within the animal house. Movie presentations lasted 60 s for imaging experiments and 35 s for electrophysiology experiments.

In vivo ATP imaging

We used a transgenic mouse line B6-Tg(Thy1.2-ATeam1.03^{YEMK})^{AJhi} that expressed the FRET-based ATP sensor ATeam1.03^{YEMK} under the Thy1.2 promoter (Trevisiol et al., 2017) (RRID:MGI:5882597; https://scicrunch.org/resources). On the day of experimentation, a given animal was anesthetized with isoflurane gas and administered carprofen (Carprieve, 5 mg/kg), along with 25 mL/kg of warm Ringer's solution subcutaneously. A custom metal headplate was first superglued on to the skull and fixed with dental cement (Paladur, Heraeus Kuzler). A small cranial window (~0.5 x ~0.5 mm) was then made above the left visual cortex and covered with a 4% agarose solution, followed by application of silicone to ensure a good seal. The dura was kept intact. The animal was allowed to recover (30-60 minutes) prior to being head-fixed and imaged, during which time the silicone and agarose were removed and replaced with HEPES-buffered ACSF (in mM: 124 NaCl, 20 Glucose, 10 HEPES, 2.5 KCl, 1.2 NaH_2PO_4, 2 $CaCl_2$, and 1 $CaCl_2$; pH 7.2-7.4). For two-photon imaging, the laser was tuned to 850 nm for excitation. CFP and YFP fluorescence were recorded simultaneously using a 515nm long-pass dichroic mirror with 485/70nm (CFP) and 535/45nm (YFP) emission filters (mirror and filter set: T515lpxr C156624; Scientifica). Imaging was performed during the presentation of an outdoor movie (60 s/trial). Three trials were taken at baseline, after which ATP synthesis inhibitors (1 mM oligomycin and 20 mM sodium iodoacetate) were added to the ACSF to isolate ATP usage. Thirty trials were successively performed immediately after drug application. We confirmed that there were no differences between control and food restricted groups in FRET decay with drug application alone, in the absence of visual stimulation (FRET decay in CTR group versus FR group; −0.084 ± 0.003/s versus −0.080 ± 0.005/s; t test: p = 0.56; n = 8 CTR and 6 FR animals)

In vivo electrophysiology

On the day of experimentation, a given animal was anesthetized with isoflurane gas and administered carprofen (Carprieve, 5 mg/kg), along with 25 mL/kg of warm Ringer's solution subcutaneously. A custom metal headplate was first superglued on to the skull and fixed with dental cement (Paladur, Heraeus Kuzler). Following, a small cranial window (no more than 0.5 × 0.5 mm) was made above the left visual cortex and covered with a 4% agarose solution, followed by application of silicone to ensure a good seal. The dura was kept intact. The animal was allowed to recover (30-60 minutes) prior to being head-fixed and imaged, during which time the silicone and agarose were removed and replaced with HEPES-buffered ACSF.

Patch recordings were obtained using a Multiclamp 700B (Molecular Devices) amplifier and WinWCP software (University of Strathclyde Glasgow). Data were acquired at 20 kHz and filtered at 3 kHz using a digitizer (Digidata 1440, Axon Instruments). Borosilicate patch electrodes (4-6 MΩ; 1.5 mm outer diameter; 0.86 mm inner diameter; Harvard apparatus) were pulled to have a long taper using a two-step pull on a Narashige vertical puller (PC-100) and were filled either with a potassium-based internal solution for current clamp recordings (in mM: 130 Kgluconate, 10 KCl, 10 HEPES, 2 Na_2ATP, 0.4 Na_3GTP, 2 $MgCl_2$, and 0.3 EGTA; pH 7.2-7.4) or a cesium-based internal solution for voltage-clamp recordings (in mM: 140 $CsMeSO_4$, 10 HEPES, 2 Na_2ATP, 0.4 Na_3GTP, 2 $MgCl_2$, 0.3 EGTA, 5 QX314-Cl and 5 TEA-Cl; pH 7.2-7.4).

In vivo blind patch recordings were made as previously described (Adesnik, 2017; Brown et al., 2019). Electrodes were placed in the bath and advanced with a positive pressure (300 mBar) at a 45° angle using a microdrive (Scientifica) while electrode resistance was monitored. Electrode depth was zeroed upon a sudden increase in resistance, which indicated contact with the dura. The electrode was rapidly advanced by ~100 μm to break the dura; electrodes were discarded if resistance did not rapidly return to within 5% of its original value. After successful penetration, the electrode was advanced to 150 μm below the pia surface and positive pressure was reduced to 20-30 mBar. The pipette was then slowly advanced in 2 μm steps. A bounce-like increase in resistance in response to each of 3 successive steps indicated contact with a putative cell, at which point positive pressure was released and a gigaohm seal was attempted by applying slight negative pressure if necessary. Electrodes were discarded on failed attempts, or if no cell was encountered within 350 μm of the pia surface. Following formation of a gigaohm seal, negative pressure was used to break-in. Whole-cell recording (20-40 MΩ) was subsequently optimized by applying slow negative or positive pressure. Pipette capacitance was fully compensated. The quality of recording was constantly monitored using a negative

current or voltage step. Only neurons with stable series resistance were used (< 20% change throughout the recording). The liquid junction potential was not corrected. Excitatory and inhibitory currents were recorded at −70 mV and +10 mV. In experiments assessing voltage-dependent subthreshold variability, the resting membrane potential was hyperpolarized by injecting hyperpolarizing current (−50pA to −100pA) of sufficient magnitude to prevent spiking during grating presentations. We obtained current clamp recordings from a total of 33 control and 26 food-restricted animals, and voltage-clamp recordings from a total of 13 control and 10 food-restricted animals.

Ex vivo electrophysiology

Acute slices were prepared from the visual cortex. The brain was extracted and coronally sectioned (400 μm thick) in ice-cold dissection media (in mM: 87 NaCl, 75 sucrose, 2.5 KCl, 25 NaHCO3, 1.25 NaH2PO4, 25 glucose, 0.5 CaCl2, 7 MgCl2; 95% O_2, 5% CO_2; pH 7.2-7.4) using a vibratome (VT1200s, Leica, Germany). Slices containing V1 were isolated and allowed to recover for 30–60 minutes in heated (35°C) ACSF (in mM: 125 NaCl, 2.5 KCl, 25 NaHCO3, 1.25 NaH2PO4, 25 glucose, 2 CaCl2, 1 MgCl2; 95% O_2, 5% CO_2; pH 7.2-7.4) before being stored at room temperature.

During recordings, slices were perfused with heated (33°C) ACSF (6-8 mL/minute). Cells were visualized with IR-DIC illumination (BX-51; Olympus), first with a 4x objective lens (0.1 N.A.; Olympus), and then with a 20x water-immersion lens (1.0 N.A.; Olympus). Layer 2/3 pyramidal cells were patched with borosilicate glass electrodes (4–7 MΩ) pulled on a horizontal electrode puller (P-97 Sutter Instruments). Electrodes were filled either with a potassium-based internal solution for current clamp recordings or a cesium-based internal solution for voltage-clamp recordings. Cellular electrophysiology was recorded using a Multiclamp 700B amplifier and associated pCLAMP 10.0 software (Molecular Devices). Data were acquired at 20 kHz and filtered at 3 kHz using a digitizer (Digidata 1440, Axon Instruments). Pipette capacitance was compensated. Series resistance was < 20MΩ. Only neurons with stable series resistance were used (< 20% change throughout the recording). The liquid junction potential was not corrected. Excitatory currents were recorded at −70 mV.

Evoked currents were triggered using a glass stimulating electrode coupled to a constant current stimulator (Digitimer). The electrode was placed in layer 2/3, 50-100 μm from the patched cell body, perpendicular to the long axis of the apical dendrite. A minimum of 5 trials were conducted at each stimulation intensity, with a 1 s baseline recording before stimulation and 15 s between each stimulation trial. Stimulation intensity was normalized to the mean threshold intensity required to evoke an EPSC in neurons of slices from control animals. Stimulus intensity was re-normalized each time the stimulation electrode was changed. The same stimulation electrode was used for slices from at least one control and one food-restricted animal, in randomized order. Paired-pulse stimulation consisted of two stimulation pulses delivered 70 ms apart. A minimum of 5 paired-pulse trials were conducted, 30 s apart. Miniature EPSC recordings were recorded at −70 mV for a minimum of 10 minutes/cell in the presence of 1 μM TTX.

Forced-choice visual discrimination task for assessing visual discrimination

Mice were trained in a two-alternative forced-choice visual discrimination task (Wong and Brown, 2006). Mice were placed in a trapezoid-shaped pool (140 cm long x 80 cm wide x 40 cm high; ~22°C), filled with opaque water (liquid latex; Palace chemicals, Liverpool, UK). A 56 cm divider was placed perpendicular to the wider end of the pool to form 2 arms. Extra-maze cues were obscured by a white curtain. Mice had to locate a platform (10 cm diameter) that was kept invisible (~2 cm submerged) in front of a specific visual cue (grating of specific orientation and direction present in one of the two arms). Visual stimuli were displayed on two identical computer monitors (1920 × 1080 pixels; 22-inch TK410V, LG) placed at water level at the larger side of the trapezoid pool, one associated with each arm, behind a transparent plexiglass wall (55 cm high). Visual cues of square-wave gratings (1 Hz, contrast 80%, 0.03 cpd, as seen from the edge of the platform) or an isoluminant gray screen were generated through the psychophysics toolbox (MathWorks). A black curtain was added behind the monitors to provide additional directionality, and the visible cue was removed from the escape platform. Mice were tested in three phases. First, mice were trained for 3 days with a visible platform (a visible cue was placed on top of the hidden platform) (pretraining; 4 trials/day, 15 min intertrial interval (ITI)). All mice decreased their latency to find the visible platform over three days; there was no difference between food groups (Two-way Repeated-measures *ANOVA*; CTR group versus FR group; $p = 0.58$; $n = 13$ CTR and 15 FR animals). In the second phase (visual detection; 10 trials/day, 6 days, 15 min ITI), mice were trained to swim to a hidden platform placed in the arm associated with a vertically oriented drifting grating (90°; target stimulus); the other computer screen displayed a uniform gray stimulus (non-target stimulus). The arm with the vertical grating and platform was randomized in each trial. Each trial lasted a maximum of 90 s; mice failing to find the platform were guided to the platform. If the mouse crossed the imaginary line running perpendicular to the end of the 56 cm divider (decision line) on the wrong (non-target) stimulus side, then the trial was recorded as an error, and after finding the platform, the mouse was returned immediately to the release location to perform another trial until the mouse made a correct choice or for a maximum of 3 consecutive trials. An error trial also occurred if the mouse failed to reach any platform position within 90 s. Only mice that showed over 70% performance (averaged across 2 days) were used for the next phase. In the third phase (pattern discrimination; 10 trials/day, 9 days, 15 min ITI), mice were trained to swim to the hidden platform placed in front of the computer screen with a vertically oriented drifting grating (90°, target stimulus) versus a 45° oriented drifting grating (non-target stimulus). All other aspects of pattern discrimination training followed the procedure of visual detection training. In the final phase of the experiment, mice were tested for their visual discrimination threshold by reducing the angle difference between the vertically oriented drifting grating (target stimulus) and the non-target stimulus. The following angle differences were tested: 30° (90° versus 60°; 10 trials/day, 2 days), 20° (90° versus

70°; 10 trials/day, 2 days), 10° (90° versus 80°; 10 trials/day, 2 days), 7.5° (90° versus 82.5°; 10 trials/day, 2 days), 5° (90° versus 85°; 10 trials/day, 1 day). At the end of visual perception testing, true chance was assessed by testing 4-10 trials of 0° angle difference (90° versus 90°). Finally, mice were also re-assessed in discriminating 90° versus 45° (10 trials/day, 1 day) to confirm their ability to discriminate patterns at the end of the testing experiment; both groups retained > 70% criterion performance (CTR performance: 82.31 ± 3.42%; n = 13 animals; FR performance: 78.00 ± 2.62%; t test; p = 0.32; n = 15 CTR and FR animals). All mice remained on the platform for 15 s before being removed from the pool. Platform locations were pseudorandomized across trials and counterbalanced across mouse groups. Release location was the same throughout all phases. Between trials, mice were dried with a towel and were returned to a holding cage (similar to their home cage), which was placed on a heating pad with monitored temperature. A video camera mounted above the pool recorded the sessions for offline analysis.

Leptin supplementation

Leptin levels were supplemented by twice daily intraperitoneal injections of mouse recombinant leptin (Peprotech, UK; 12.5 μg/g dissolved in saline delivered at 09:00 and 18:00; adapted from (Halaas et al., 1995)) for 10 days; controls received saline.

Serum collection and analysis

Serum glucose, β-hydroxybutyrate, adrenaline, corticosterone and leptin levels were assayed using colorimetric detection kits as per manufacturer's instructions (Glucose Colorimetric Detection Kit, ThermoFisher Scientific, UK; Ketone Body Colorimetric Assay Kit, Cayman Chemical, USA; Adrenaline ELISA Kit, BioVision, USA; Corticosterone Parameter Assay, R&D Systems, US; Mouse Leptin Quantikine Elisa Kit, R&D Systems, USA). Trunk blood was collected between 16:00-19:00 from animals that were briefly anesthetized with isoflurane prior to being decapitated. Blood was allowed to clot for 1-2 hours then centrifuged for 20 minutes at 2000 x g; the serum was drawn off and stored at −80°C until used. For sample collection from sated, food restricted animals, animals were first sated with *ad libitum* access to food for 1-3 hours prior to blood collection; food restricted animals were otherwise unfed (unsated). Animals being supplemented with leptin or saline had their injections at least 9 hours prior to sample collection.

Hodgkin-Huxley type model neuron

Numerical simulations were performed using the NEURON simulation environment (Hines and Carnevale, 1997) interfaced with Python (Hines et al., 2009). Custom channels were defined using NEURON's Channel Builder. Analysis was done in Python, channel calibration in MATLAB. Code is available on the github repository (custom channels, script simulation, and analysis).

The behavior of the membrane was represented by a simple electrical circuit, following the work of (Hodgkin and Huxley, 1952). For simplicity we used the standard single compartment model, which includes a capacitive current, a leakage current with passive conductance independent of membrane voltage, and the two ionic currents sodium (Na_v) and potassium (K_v), which account for action potential dynamics, with voltage-dependent conductances (Tables S1 and S2). For the Na_v channel kinetics, we used the Markov model with an allosteric relationship between activation and inactivation described in (Carter et al., 2012). For the delayed rectifier K_v current, we used the channel described by (Hodgkin and Huxley, 1952) but with faster kinetics, as in(Hansel and Sompolinsky, 1996) and (Wang and Buzsáki, 1996). The kinetics, maximal conductances, and voltage dependency of channels were adjusted to obtain action potentials with spike threshold and shape similar to those observed in cortical neurons. Conductances were close to values previously published in single compartments models (Golomb et al., 2006; Wang and Buzsáki, 1996); a low K_v conductance was necessary for a brief afterhyperpolarization.

Despite the fact that Na_v and K_v channels in our model contributed to subthreshold membrane depolarization, their fast gating kinetics lead to small fluctuations, which initiated spontaneous spiking over a very limited range of input in our system (see (O'Donnell and van Rossum, 2014) for a detailed analysis of the contributions of stochastic Na_v and K_v channels). Therefore, we added a slower-activating channel so as to introduce larger subthreshold fluctuations. For simplicity, we used the original Hodgkin-Huxley K_v channel, which has been well characterized and was shown to be the dominant source of membrane noise in the Hodgkin-Huxley model (O'Donnell and van Rossum, 2014; Steinmetz et al., 2000). The conductance was adjusted to get an appropriate level of noise without significantly affecting AP dynamics.

Finally, input resistance $R = 1/g_L$ and membrane capacitance C_m were set so that the membrane time constant was $\tau = C_m / g_L =$ 10 ms. Leak reversal potential was set to maintain the desired membrane potential at rest. All parameter values are summarized in Supplemental Tables S1 and S2.

Model parameters

We simulated the two main features observed in food restriction by 1) dropping the leak conductance g_L (inverse input resistance) to 79% of the control value, and 2) increasing the leak reversal potential E_L by +5 mV, thus depolarizing the resting membrane potential. We used the same channel models in both conditions, and thus the voltage dependency of the ionic currents was the same across groups. We also ran the simulations for two intermediate conditions, namely increased input conductance alone and depolarized membrane resting potential alone. See Table S1 for a summary of the four groups.

Synaptic input

Synaptic input conductance was simulated as a single large event, similar to (Liu et al., 2011), and was defined as $g(t) = g_{peak} \times f \times (e^{-t/\tau_{decay}} - e^{-t/\tau_{rise}})$, where τ_{rise} and τ_{decay} are the synaptic rise and decay time constants, set to 70 ms and 75 ms respectively. The factor f normalizes the peak of the exponential term to 1 and is automatically computed within the class *Exp2Syn* in the NEURON simulator. We only included an excitatory synapse (AMPAR) with reversal potential equal to 0 mV. In the remaining text, when we mention synaptic conductance we refer to the peak conductance g_{peak}. For each group, we determined the minimal synaptic conductance that triggered exactly one action potential, which we refer to as rheobase conductance, noted g_0^{group}. Table S1 gives the rounded values of the synaptic compensation $g_0^{group}/g_0^{control}$.

External and intrinsic noise

Two noise models were used to induce trial-to-trial variability (Figure 5), which differed depending on whether noise was generated by an external source or intrinsically. We modeled the external source of noise by injecting a different conductance at each trial. For a given average synaptic input $\overline{g}$, we sampled a conductance from a normal distribution with mean g and standard deviation σ that scaled proportionally with $\overline{g}$. If we note σ_0 the standard deviation for the control group at rheobase conductance $g_0^{control}$, variability was then scaled for each group (Table S1) by a factor ρ such that $\sigma = \rho \times \sigma_0$, where $\rho = \overline{g}/g_0^{group}$. The standard deviation σ_0 was set to approximately match the level of fluctuations observed with stochastic channels. For the intrinsic noise model, intrinsic variability was simulated with stochastic ion channels, thus noise was voltage-dependent. Only the subthreshold channel was stochastic, while Na_v and K_v were kept deterministic. The surface area was 200 μm^2.

Simulations and analysis

For each group and noise model, we varied the input conductance proportionally to rheobase and recorded the probability of generating at least one spike. The data was fitted with a generalized logistic model. For the input-output curves shown in Figure 5, we took an interval up to rheobase conductance and scaled the response to its maximum value. We then generated a set of synaptic conductances from a Gaussian tuning curve (parameters: amplitude = 0.9; width = 170°) and using the fitted input-output curve we inferred the spike output probability. We fitted a Gaussian curve to the normalized spike probability. To quantify the level of trial-to-trial variability in the system, we measured the maximum depolarization at each trial (97.5th quantile) after clipping the AP, and computed the CV as the ratio between the standard deviation of this depolarization divided by the average response, defined as V_m- V_{Rest}.

Integrate-and-fire model neuron

The model (Figure 3H) is an integrate-and-fire model with passive elements, an input resistance $R = 1/g_L$, and membrane capacitance C_m set to the same values as the Hodgkin-Huxley-type model (Tables S1 and S2). An action potential was counted when the membrane voltage reached −40 mV. For the integrate-and-fire model (Figure 3H) the rheobase synaptic conductance was 30% less for food-restricted versus control group, as experimentally observed. For both groups, control and food-restricted, we injected a synaptic conductance proportional to the respective rheobase conductance, from 0 to 100%, and recorded the membrane depolarization from V_{Rest}. We then normalized the resulting depolarization to the distance to spike threshold, defined as the distance from V_{Rest} to $V_{Threshold}$ (Figure 3H).

QUANTIFICATION AND STATISTICAL ANALYSIS

Pupil analysis

Pupil diameter was quantified using custom MATLAB script. Briefly, the script utilized built-in MATLAB functions (including the Image Processing Toolbox) to accomplish the following steps:

1) Resize the pupil video - *imresize* (to increase processing speed)
2) Remove noise and adjust contrast – *medfilt2, imadjust*
3) Segment the image to select the pupil – *imbinarize, imclearborder, bwpropfilt*
4) Fit an ellipsoid to the segmented area – *regionprops*

The pupil diameter (d) was calculated off the basis of the ellipsoid as

$$d = 2\sqrt{semi - major\ axis * semi - minor\ axis}$$

The pupil diameter was calculated for each image frame and averaged across frames in the trial.

Ca^{2+} Imaging Analysis

Image analysis for two-photon calcium imaging was performed as previously described (Henschke et al., 2020; Pakan et al., 2016, 2018). Briefly, a discrete Fourier 2D-based image alignment was used for motion correction of image frames (SIMA 1.3.2) (Kaifosh et al., 2014). Regions of interest (ROI) were manually drawn around neuronal cell bodies on average intensity projections using

ImageJ software (NIH public domain; RRID:SCR_002285). Pixel fluorescence within each ROI was averaged to generate a time series. Baseline fluorescence (F_0) was computed for each ROI by taking the 5th percentile of the smoothed time series (1 Hz lowpass, zero-phase, 60th-order FIR filter); ΔF/F was calculated as (F-F_0/F_0). Neuropil decontamination of ROI signals was done using a custom toolbox (FISSA), which uses nonnegative matrix factorization (NMF) to perform blind source separation (Keemink et al., 2018). Subsequent analyses were performed using custom scripts in MATLAB (MathWorks).

Analysis of neuronal responses to drifting gratings

For calcium imaging, owing to the slow kinetics of GCaMP6s, the visual response period was defined as a 4 s period (2 s grating plus the following 2 s of gray screen presentation). The visual response was defined as the highest mean ΔF/F within a 2 s window during the visual response period, subtracted by baseline ΔF/F, defined as the mean value within a 1 s window prior to the visual response.

For current clamp electrophysiology, visually-evoked spiking responses were defined as the spike rate averaged across the 2 s grating presentation minus the mean spike rate in response to gray screens. To calculate visually-evoked subthreshold responses, action potentials were removed by replacing voltage values above threshold with NaN values (adapted from (Azouz and Gray, 1999)). Average subthreshold responses were then characterized by taking the median membrane potential value during the 2 s grating presentation minus the median membrane potential value in the preceding 1 s of gray screen. To calculate trial-to-trial variability, we used the 95th percentile response instead of the median to capture the large deviations of the membrane potential that happened within a trial. For voltage-clamp responses, gratings were characterized by taking the median membrane current during the 2 grating presentations and subtracting the median membrane potential value in the preceding 1 s gray screen.

Responses to gratings of different drift directions but the same orientation were averaged together. If multiple spatial frequencies were used, the spatial frequency with the largest response, meaned across all orientations, was selected as the preferred spatial frequency. A neuron's preferred orientation was defined as that associated with the largest mean response at its preferred spatial frequency.

Response tuning was characterized by fitting responses with Gaussian curves using a procedure adapted from (Mazurek et al., 2014). Tuning width was defined as 1 standard deviation (σ) of the fitted curve.

Orientation responses were fit with a single Gaussian curve:

$$R(\theta) = C + R_p e^{\frac{-ang_{ori}\left(\theta-\theta_{pref}\right)^2}{2\sigma^2}}$$

Where R (θ) is the response at a given orientation angle θ, C is a constant offset, R_p is the response to the preferred orientation after subtracting the offset, θ_{pref} is the angle of the preferred orientation,

$ang_{ori}(x) = \min(x, x-180, x+180)$, which constrains angular differences to 0-90°, and σ is the standard deviation of the curve.

Direction responses were fit with a double Gaussian curve:

$$R(\theta) = C + R_p e^{\frac{-ang_{dir}\left(\theta-\theta_{pref}\right)^2}{2\sigma^2}} + R_n e^{\frac{-ang_{dir}\left(\theta-\theta_{pref}\right)^2}{2\sigma^2}}$$

Where R (θ) is the response at a given direction angle θ, C is an offset, R_p is the response to the preferred direction after subtracting the offset, θ_{pref} is the angle of the preferred direction, R_n is the response to the null direction after subtracting the offset, and $ang_{dir}(x) = \min(x, x-360, x+360)$, which constrains angular differences to 0-180°, and σ is the standard deviation of the curve.

As in (Mazurek et al., 2014), Gaussian fits were constrained to optimize fitting. Optimal fits were found when R_p was constrained to lie between (mean response to preferred stimulus/2, mean response to preferred stimulus). For ΔF/F responses, optimal fits were obtained when C was constrained to 0. Fitting was initialized at several values of σ; the best fit had the lowest least square error.

Orientation selective index (OSI) was calculated as 1 – circular variance from the mean response to each presented orientation; values less than zero were set to zero.

$$OSI = abs\left(\frac{\sum_k R(\theta_k)e^{2i\theta_k}}{\sum_k R(\theta_k)}\right)$$

Grating-responsive neurons

For calcium imaging experiments, grating responsive neurons were defined as those for which grating responses were better fit with a double Gaussian curve (direction responses) than with a flat line at zero (null model). We used the Bayesian Information Criterion (BIC) to assess model selection:

$$BIC = n\ln\left(\overline{\sigma^2}\right) + k\ln(n)$$

Where n is the number of responses, $\overline{\sigma^2}$ is the mean residual sum of squares of the model, and k is the number of free parameters used by the model, which was zero in the case of the null model. A neuron was considered significantly grating-responsive if the

BIC_{null} - $BIC_{Gaussian} \geq 10$, which provides strong evidence against the null model (Kass and Raftery, 1995). Only grating-responsive neurons were included in the analysis of Figures 6B and 6C, Figure 7C, and Figure S6.

Natural stimuli-responsive neurons

For calcium imaging experiments using natural movies, we first extracted spikes from ΔF/F traces (MLSpike (Deneux et al., 2016)). We segmented natural movies into 58 one-second bins. A neuron was considered to be responsive to the movie if it responded to any one bin with a mean response value that was $\geq$ 5 times the standard deviation of its response, across all trials in that bin. There was no significant difference in the proportion of responsive neurons between the control and food-restricted group (CTR group versus FR group: 0.38 ± 0.02 versus 0.36 ± 0.01 t test: p = 0.45; n = 6 CTR and 7 FR animals).

Decoding

Drifting gratings

A maximum likelihood estimator was used to decode orientations based on population responses recorded with calcium imaging, as previously described (Montijn et al., 2014). Briefly, we used a leave-one-out procedure to first calculate the mean (μ) and standard deviations (σ) of ΔF/F responses associated with each presented orientation for a given neuron. We then calculated the log likelihood for the left-out response given the μ and σ of each orientation, assuming a standard Gaussian distribution. Log likelihoods pertaining to the same orientation were summed across neurons. The orientation associated with the maximum log likelihood was selected as the decoded orientation. Decoding accuracy was calculated as the proportion of correctly decoded orientation. For each animal, decoding was assessed using the same number of neurons (50 neurons) that were randomly sampled from the pool of grating-responsive neurons 100 times without replacement; results were averaged across samples. Decoding accuracy was assessed for each presentation of an orientation (16-20 trials/stimulus). Decoding accuracy for an animal was averaged across all stimuli.

Maximum likelihood estimators were also tested on simulated data. Here, the orientation tuning curve for each grating-responsive, neuron was fit with a Gaussian (Montijn et al., 2014) to give μ response values for any orientation. Response variability was calculated at each experimentally recorded orientation as the co-efficient of variation ($CV = \sigma/\mu$); the CV was meaned across orientations. For any orientation, σ was calculated as μ x CV. Sets of orientations with angles spanning from 0-180° and with a given spacing interval between orientations (5°, 7.5°, 10°, 15°, 20° or 30°) were then defined. For each defined orientation, responses were generated for each neuron given the μ and σ at that orientation. For each orientation set, the decoding accuracy of a maximum likelihood estimator was tested on the simulated responses as described for experimental responses. The entire simulation was repeated 1000 times and the results were averaged.

Natural stimuli

Maximum likelihood estimators were also used to decode natural scenes from a presented movie based on population responses recorded with calcium imaging. We first extracted spikes from ΔF/F traces (MLSpike (Deneux et al., 2016)) and calculated the μ and σ of the spike rate in 1 s bins, which defined a scene, using the same leave-one-out procedure; decoding performance was then calculated using a similar procedure as with gratings. The same number of natural stimuli-responsive neurons was used across animals, and were randomly sampled from the total pool of responsive neurons 100 times without replacement; results were averaged across samples. For decoding scenes from within an environment, the decoder had to choose the correct scene from 58 scenes, all drawn from the outdoor movie. We used a scene dissimilarity score to capture the visual differences between presented scenes. We calculated the absolute difference between the intensities (on a 0-255 scale) of corresponding pixels between two scenes, meaned across all pixels and scene pairs. For decoding scenes from different environments, 29 scenes were drawn from the outdoor movie, and 29 from the home cage movie. A correct trial required the decoder to choose any scene belonging to the same movie of the tested scene. Here, the dissimilarity score was calculated as the absolute difference between the intensities of corresponding pixels between two scenes, meaned across all pixels and scene pairs; scene pairs consisted of scenes from different movies. Analysis was based on 8-10 trials/scene.

ATP measurements

ATeam Imaging

For ATeam imaging, YFP and CFP fluorescence was calculated as the background subtracted fluorescence averaged across the entire imaging field. FRET was calculated as a ratio of YFP/CFP, which decreased as a function of time as ATP was consumed (Baeza-Lehnert et al., 2019; Lerchundi et al., 2020; Trevisiol et al., 2017). The FRET signal was bound between 0 and 1, by subtracting the mean FRET signal during the last 3 imaging trials, during which the FRET signal plateaued, and then dividing by the mean FRET signal at baseline. FRET decay rate was defined as the slope of a linear fit of the decay curve, without the first 3 (baseline) and last 3 data points, during which the rate had plateaued.

ATP usage estimation from electrophysiological recordings

ATP usage at the soma was estimated from electrophysiological recordings, as previously described (Attwell and Laughlin, 2001; Harris et al., 2015). To calculate the ATP usage rate associated with excitatory synaptic signaling, excitatory currents were first recorded in voltage-clamp (−70 mV) in response to an outdoor natural movie (35 s clip; 1-5 repeats). The current trace was integrated

and divided by the duration of recording to get a mean rate of charge transfer. This was subtracted from the charge transfer rate recorded at baseline (gray screen). The value was then multiplied by 1.42 to estimate the Na^+ influx rate associated with charge transfer, which took into account that current influx is comprised of 1.42 times more Na^+ than necessary, owing to concurrent K^+ efflux (Harris et al., 2015). This value was then divided by 3 to derive ATP usage rate, since it takes approximately 1 ATP to extrude 3 Na^+ ions via the Na^+/K^+ pump.

ATP usage at the soma associated with spiking activity was calculated as (Attwell and Laughlin, 2001; Harris et al., 2015):

$$\frac{ATP}{s} = \frac{1.35x\ 10^{11}\ ATP}{spike\ x\ cm^2}\ x\ \frac{capacitance\ (\mu F)}{specific\ capacitance\ \left(\frac{\mu F}{cm^2}\right)}\ x\ spike\ rate\ \left(\frac{spikes}{s}\right)$$

Where spike rate and capacitance were obtained from current clamp recordings during presentation of an outdoor natural movie (35 s clip) after subtracting baseline firing rate (gray screen), specific capacitance was taken to be 1 $\mu F/cm^2$, the standard biological membrane capacitance (Gentet et al., 2000), and 1.35×10^{11} $ATP/spike/cm^2$ was taken from the estimated ATP consumption rate for a regular spiking, rodent sensory cortical neuron (Moujahid et al., 2014).

Resting ATP expenditure was calculated as (Attwell and Laughlin, 2001):

$$\frac{ATP}{s} = (V_{Na} - V_K)(V_{Rest} - V_K)A/(FR(V_{Rest} - 2V_{Na} - 3V_K))$$

Where V_{Na} and V_K are the reversal potentials for sodium (50 mV) and potassium (−100 mV) respectively, R is input resistance, V_{Rest} is the resting membrane potential, A is Avogadro's constant, and F is the Faraday constant.

Analysis of patch-clamp electrophysiological recordings

Intrinsic parameters

During current clamp recordings, 500 ms current pulses were delivered in 50 pA steps from – 200 to + 200 pA to assess the input resistance (R_m), membrane time constant (τ_m), and membrane capacitance (C_m) using pCLAMP 10.0 software and custom MATLAB scripts. Pulses were delivered in the absence of evoked synaptic stimulation (*ex vivo*) or during the presentation of gray screen stimuli (*in vivo*). The decay phase of the voltage at the end of each current step was fit to a double exponential:

$$V(t) = I_m + R_m\left(1 - e^{-\frac{t}{\tau_m}}\right) + R_a\left(1 - e^{-\frac{t}{\tau_a}}\right)$$

Where V is the voltage, t is time, I_m is the amount of current injected, R_a is the access resistance, τ_a is the electrode time constant. Values for R_m and τ_m were averaged across all current steps in the recording. C_m was then solved for using $C_m = \tau_m / R_m$.

Resting membrane potential was calculated using the 5th percentile of the recorded membrane potential recorded either in the absence of evoked synaptic stimulation (*ex vivo*) or during the presentation of gray stimuli (*in vivo*). For a given cell, resting membrane potential was averaged across trials.

Spikes were automatically detected using the *findpeaks* function (MATLAB). Spike threshold was the voltage potential at the time point that maximized the second derivative of the membrane potential in a 5 ms time window preceding the peak of a spike. For a given cell, spike threshold was calculated for all recorded spikes and then averaged.

In vivo input-output curves

Current clamp recordings during visual stimulation (drifting gratings) were parsed into 100 ms time bins. Spikes were counted and removed by replacing voltage values above spike threshold with NaN values (adapted from (Azouz and Gray, 1999)). The median subthreshold potential was then quantified and normalized by subtracting out the resting membrane potential and dividing by the calculated spike threshold. The resulting value reflected the fraction (0-1) of the total spiking distance the cell was depolarized in the time bin. Bins with similar depolarizations were grouped (e.g., group 1: 0-0.1, group 2: 0.1-0.2, etc). Within a group, the spiking activity across bins was averaged. Probability of spiking was also calculated as the number of bins with at least one spike divided by the total number of bins in the group. Spike rate and spike probability were then plotted against normalized depolarization to obtain input-output curves.

Ex vivo EPSC measures

Excitatory postsynaptic responses (EPSCs), evoked by stimulation, were characterized by their maximum amplitude. Input-output curves were generated by plotting mean EPSC amplitude against stimulation intensity. Paired-pulse ratio, a measure of presynaptic efficacy was quantified as the average peak EPSC in response to the second of two stimulation pulses, divided by the average peak EPSC in response to the first pulse; a minimum of 5 trials were averaged. $1/CV^2$, a measure of presynaptic efficacy, was quantified from the CV of EPSC responses, taken as the σ/μ calculated from a minimum of 20 trials.

Ex vivo mEPSC analysis

Miniature EPSCs (mEPSCs), recorded in the presence of TTX, were detected using the MATLAB *findpeaks* function. mEPSC amplitude was calculated as the peak amplitude. A minimum of 10 minutes of activity was recorded for each cell.

Ex vivo mean variance analysis

Mean variance analysis was conducted on mEPSCs, as previously described (Traynelis et al., 1993; Yamashita et al., 2003). Briefly, for each neuron, a minimum of 50 mEPSCs with well-defined amplitudes (> 5 pA) were averaged to form a template. Each mEPSC was then subtracted from the peak-scaled template at its decay phase. The resulting difference trace was divided into 30 equal bins on the basis of equal fractional reductions in amplitude, with values averaged within a bin. The process was repeated for each mEPSC. The ensemble mean and variance of values at each bin were calculated and plotted as variance against mean. Assuming binomial statistics of channel opening, the initial slope of the plot is an estimate of open channel conductance. Dividing the mean mEPSC amplitude by this value yielded the average open channel number.

Behavior

Performance was calculated as the percentage of correct choices on each day (10 trials). For the testing phase (grating discrimination), performance was calculated as the percentage of correct choices across 20 trials over 2 days (10 trials/day). Swim distance and swim speed were calculated using ANY-maze (Stoelting, Europe); calculations were based on the full path trajectory taken by the animal from the start point to the platform across all trials. There were no group differences in either the relationship between swim distance and performance (CTR versus FR; R^2: 0.76 ± 0.06 versus 0.65 ± 0.08; t test: $p = 0.32$; slope: −0.02 ± 0.002 versus −0.02 ± 0.004; Mann-Whitney U test: $p = 0.89$; n = regressions of swim distance and performance for 13 CTR and 15 FR animals) or the relationship between swim speed and performance (CTR versus FR; R^2: 0.40 ± 0.09 versus 0.25 ± 0.07; t test: $p = 0.18$; slope: −0.0002 ± 0.0007 versus −0.0002 ± 0.0004; Mann-Whitney U test: $p = 0.24$; n = regressions of swim distance and performance for 13 CTR and 15 FR animals).

Gaussian noise model of orientation tuning

The model determined the probability a subthreshold depolarization (evoked by a given stimulus grating) would cross spike threshold given: 1) its average amplitude (μ), and 2) its associated variability (σ). At each experimentally-presented grating, we used the experimentally-calculated average amplitude (μ) and CV (σ/μ) of the subthreshold response, meaned across all cells within a group (control or food-restricted) after mapping orientations to a −90 to 90 space, depending on their distance to the cell's preferred orientation. The subthreshold responses were assumed to be symmetric, so responses associated with orientations at the same absolute distance from the preferred orientation were averaged. The CV was assumed to be constant across orientations and meaned to get a single value, which was then multiplied by the calculated amplitude of depolarization for each orientation to obtain a σ for each orientation. The spike threshold was also meaned across cells within a group (control or food-restricted). We then calculated the likelihood of the spike threshold value given the μ and σ for each angle assuming a Gaussian distribution. This was then normalized to the likelihood of spiking for the preferred stimulus to give an orientation tuning curve of spike probability.

Statistics

All statistical tests and associated details, including n values and what n represents, are stated in the figure legends; significance was defined as $p < 0.05$. For imaging experiments, statistics were performed with animals as the statistically independent unit. For electrophysiological recordings, statistics were performed on cells as a statistically independent unit; each group had a minimum of 4 animals. Sample size was determined *a priori* based on calculations to achieve 80% power. Power calculations assumed a 20% group difference, with group means and variances taken from pilot data or previously published data. For multiple comparisons, ANOVA tests were used, with repeated-measures where applicable, followed by post hoc Sidak's tests. For behavioral training, we used a Mixed-effects model (REML). For behavioral testing, we used *a priori* tests with Sidak's correction to test planned comparisons (Ruxton and Beauchamp, 2008), which we based on group differences (food-restricted versus control animals) in orientation decoding of neuronal activity (Figure S5F). Single comparisons were assessed using two-tailed t tests and, in cases where assumptions of normalcy were violated, two-tailed Mann-Whitney U tests. Spike rates were log transformed prior to testing to achieve normalcy. Statistical tests were carried out in Prism 6 (GraphPad Prism; RRID:SCR_002798). Averages denoted in figures represent means, with error bars representing the standard error of the means.

Neuron, Volume *110*

Supplemental information

Neocortex saves energy by reducing coding precision during food scarcity

Zahid Padamsey, Danai Katsanevaki, Nathalie Dupuy, and Nathalie L. Rochefort

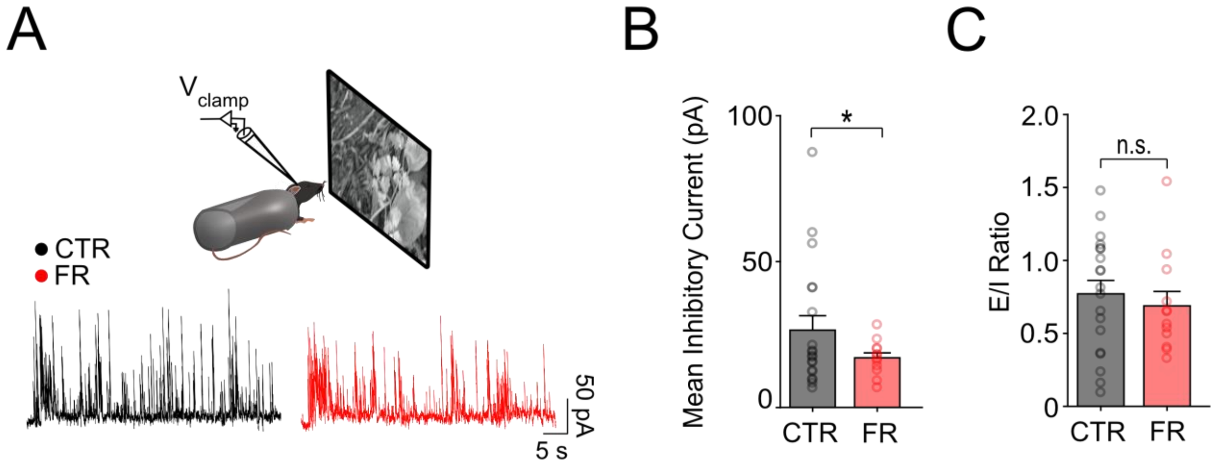

Figure S1. Excitatory/inhibitory balance of postsynaptic currents is preserved with food restriction. Related to Figure 1. (A) Sample inhibitory current traces recorded in voltage clamp during presentation of natural scenes. (**B)** Mean inhibitory current, calculated as the rate of inhibitory charge transfer (t-test; n= 19 CTR and 12 FR cells). (**C)** Mean excitatory/inhibitory (E/I) ratio (t-test; p = 0.56; n = 19 CTR and 12 FR cells). *p<0.05. Error bars are S.E.M.

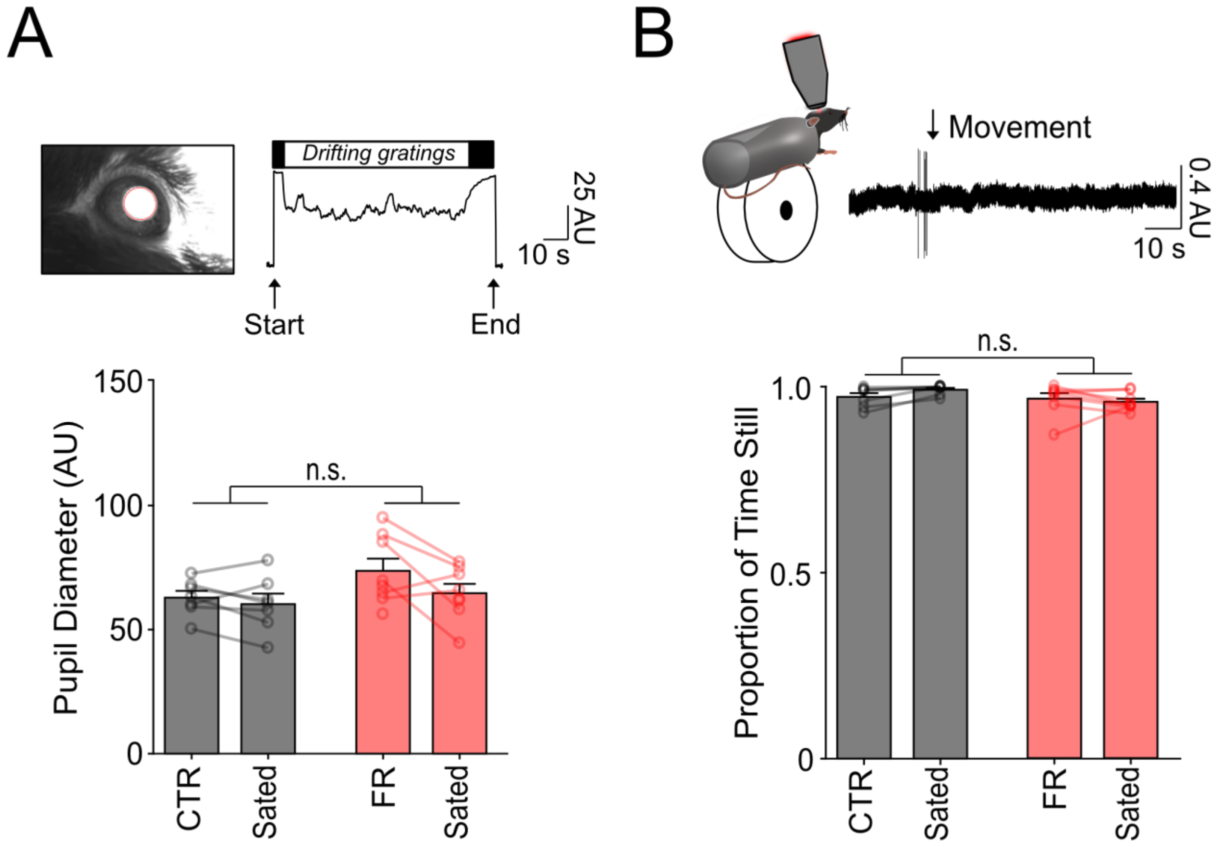

Figure S2. Food restriction does not impact behavioral state as measured by pupil size and fidgets/postural adjustment. Related to all Figures. (A) Top: Sample tracking and detection of pupil diameter (red circle) during presentation of drifting gratings. Bottom: Mean pupil diameter was unchanged across groups, regardless of whether animals were sated or not (Two-way Repeated Measures ANOVA; CTR group vs. FR group: p= 0.07; CTR group vs. FR group: p= 0.15; n = 7 CTR and 8 FR mice). (**B)** During recording sessions, animals were placed in a cardboard tube and balanced on top of Styrofoam wheel, which was wired to an optical encoder. Postural adjustments and fidgets that triggered subtle movements of the wheel could be detected. The proportion of these events were not significantly different across groups, regardless of whether animals were sated or not (Two-way Repeated Measures ANOVA; CTR group vs. FR group: p = 0.53; CTR sated vs. FR sated: p = 0.13; n = 7 and 8 animals). Error bars are S.E.M.

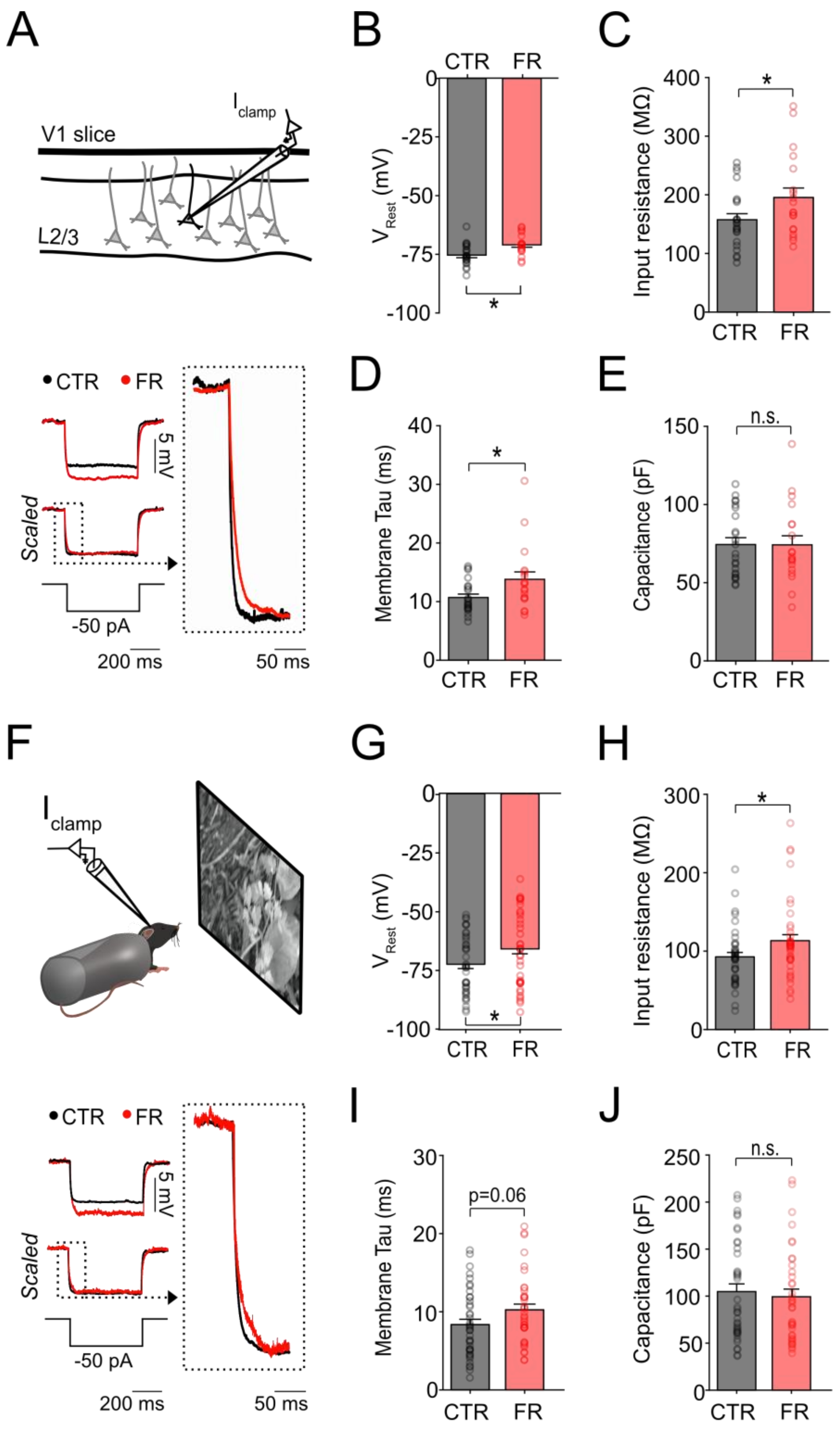

Figure S3: ***Ex vivo*** **recordings also show increased input resistance and resting membrane potential depolarization of V1 layer 2/3 neurons from food-restricted mice. Related to Figure 1 and 3. (A)** Top: Schematic of current clamp recordings *ex vivo*. Bottom: Sample membrane potential traces in response to hyperpolarizing current. The first set of traces highlights group differences in input resistance. The scaled traces have been normalized to peak and overlaid to examine group differences in membrane decay. The region denoted by a broken rectangle is magnified on the right to emphasize group differences in membrane decay. **(B)** Mean resting membrane potential (t-test; n = 23 CTR and 19 FR cells). (**C**) Mean input resistance (t-test; n = 23 CTR and 19 FR cells). (**D**) Membrane tau (t-test; p=0.97; n=23 CTR and 19 FR cells). (**E**) Mean capacitance (t-test; p=0.97; n = 23 CTR and 19 FR cells).

(**F)** Top: Schema of current clamp recording *in vivo*. Bottom: Sample membrane potential traces in response to hyperpolarizing current. The first set of traces highlights group differences in input resistance. The scaled traces have been normalized to peak and overlaid to examine group differences in membrane decay. The region denoted by a broken rectangle is magnified on the right to emphasize group differences in membrane decay. **(G)** Mean resting membrane potential (t-test; n = 40 CTR and 37 FR cells). Data from Figure 3F. **(H)** Mean input resistance (t-test; n = 40 CTR and 37 FR cells). Data from Figure 3B. **(I)** Membrane tau (t-test; p=0.06; n=40 CTR and 37 FR cells). **(H)** Mean capacitance (t-test; p=0.64; n = 40 CTR and 37 FR cells). *p<0.05. Error bars are S.E.M.

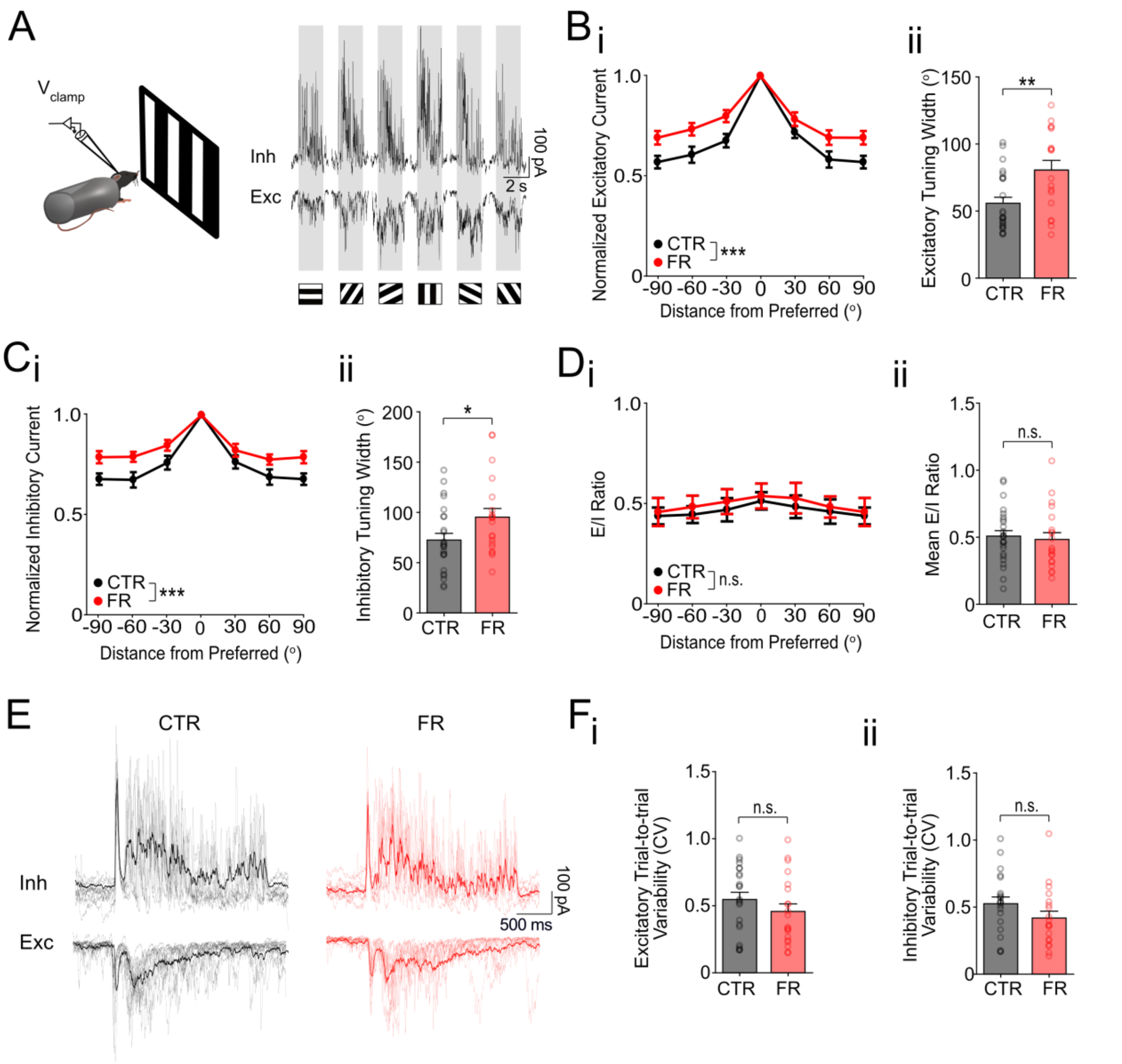

Figure S4: Food restriction broadens the orientation tuning of excitatory and inhibitory presynaptic inputs, but does not impact their variability. Related to Figure 4. (A) Left: Schema of voltage-clamp recordings. Right: Sample excitatory (Exc) and inhibitory (Inh) currents evoked by drifting gratings. **(Bi)** Orientation tuning curve of excitation, normalized to preferred stimulus response. Note that -90° and +90° conditions correspond to the same visual stimulus. (Two-way Repeated Measures ANOVA; CTR group vs. FR group is shown; n = 25 CTR and 20 FR cells). **(Bii)** Mean excitatory tuning width (t-test; n = 25 CTR and 20 FR cells). (**Ci)** Orientation tuning curve of inhibition, normalized to preferred stimulus response. (Two-way Repeated Measures ANOVA; CTR group vs. FR group is shown; n= 25 CTR and 20 FR cells). The subthreshold tuning of inhibitory input is consistent with previous studies. **(Cii)** Mean inhibitory tuning width (t-test; 25 CTR and 20 FR cells). **(Di)** Orientation tuning curve of excitatory/inhibitory (E/I) ratio (Two-way Repeated Measures ANOVA; CTR group vs. FR group: p = 0.34; n= 25 CTR and 20 FR cells). **(Dii)** Mean E/I ratio, averaged across orientations (t-test; p = 0.70; 25 CTR and 20 FR cells). (**E)** Sample traces depicting the trial-to-trial variability (faded traces) and mean (bold traces) of stimulus-evoked excitatory (Exc) and inhibitory (Inh) currents. **(Fi)** Mean co-efficient of trial-to-trial variability (CV) for excitatory currents (t-test; p = 0.63; n = 19 CTR and 17 FR cells). (**Fii)** Mean co-efficient of trial-to-trial variability for inhibitory currents (standard deviation/mean) (t-test; p=0.14; n = 21 CTR and 19 FR cells). *p<0.05; ** p< 0.01; *** p<0.001. Error bars are S.E.M.

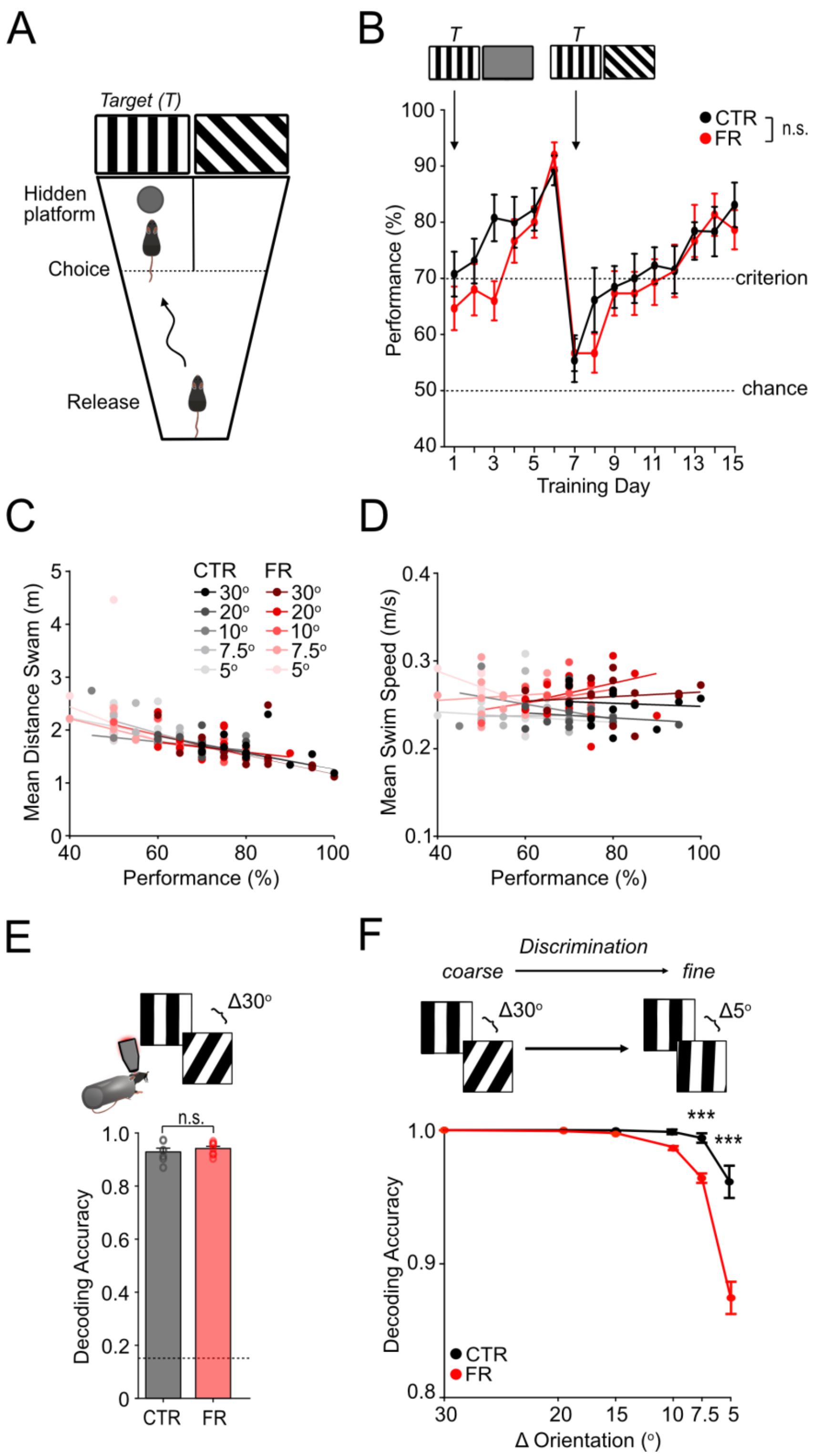

Figure S5: Acquisition of the visual discrimination task and model-based predictions of visual discriminability from V1 calcium imaging data. Related to Figure 6. (A) Schematic for behavioral experiments. Animals were placed in a modified water Y-maze and had to choose the arm associated with a target grating in order to reach a hidden platform and escape the water. (**B)** Task acquisition. A vertical drifting grating was the target throughout the task. On Day 1-6 animals were given a vertical drifting grating (target stimulus) and a grey screen to learn the association of grating-platform. On Day 7-15, they were given the same vertical drifting grating (90°, target stimulus) and a 45° oriented drifting grating (non-target stimulus). Criterion for inclusion in subsequent behavioral tasks was

performance of $\geq$70%. (Mixed-effects Model (REML); CTR group vs. FR group; $p = 0.08$; $n = 13$ CTR and 15 FR mice). (**C)** Mean distance swam as a function of behavioral performance. Each point is the mean distance swam for a single animal averaged across trials for a defined discrimination difficulty (angle difference between target and non-target gratings of 30°, 20°, 10°, 7.5° and 5°) plotted against performance at that discrimination difficulty ($n = 13$ CTR and 15 FR animals per regression; a total of 5 regressions are displayed each for the CTR and FR group, representing 5 discrimination difficulties). There was no group difference in the relationship between swim distance and performance (see Methods for statistics). (**D)** Mean swim speed as a function of behavioral performance. Each point is the mean swim speed for a single animal averaged across trials for a defined discrimination difficulty plotted against performance at that discrimination difficulty ($n = 13$ CTR and 15 FR animals per regression; a total of 5 regressions are displayed each for the CTR and FR group, representing 5 discrimination difficulties). There was no group difference in the relationship between swim speed and performance (see Methods for statistics). **(E)** Mean accuracy in decoding the correct orientation presented to the mice, amongst 6 equally-spaced orientations (minimum angle difference = 30°) based on layer 2/3 calcium imaging data presented in Figure 6A-C (maximum likelihood decoder; t-test; $p=0.43$; $n=7$ CTR and 8 FR mice). Dotted line: chance level. The decoder performed equally well in both groups, suggesting that food restriction did not grossly impact visual discrimination. The same number of neurons was used for decoding across animals. (**F)** Model-based prediction of visual discriminability from calcium imaging data presented in Figure 6A-C. Mean decoding accuracy as a function of discrimination difficulty (*i.e.*, angle difference between orientated gratings, 5–30°). Simulated based on experimental responses to 30° angle differences. Differences in decoding emerged when angle differences were < 10°, consistent with behavioral results on visual discriminability (Figure 6E) (Two-way ANOVA; CTR group vs. FR group: $p<0.001$; *post hoc* Sidak's tests). *** $p<0.001$. Error bars are S.E.M.

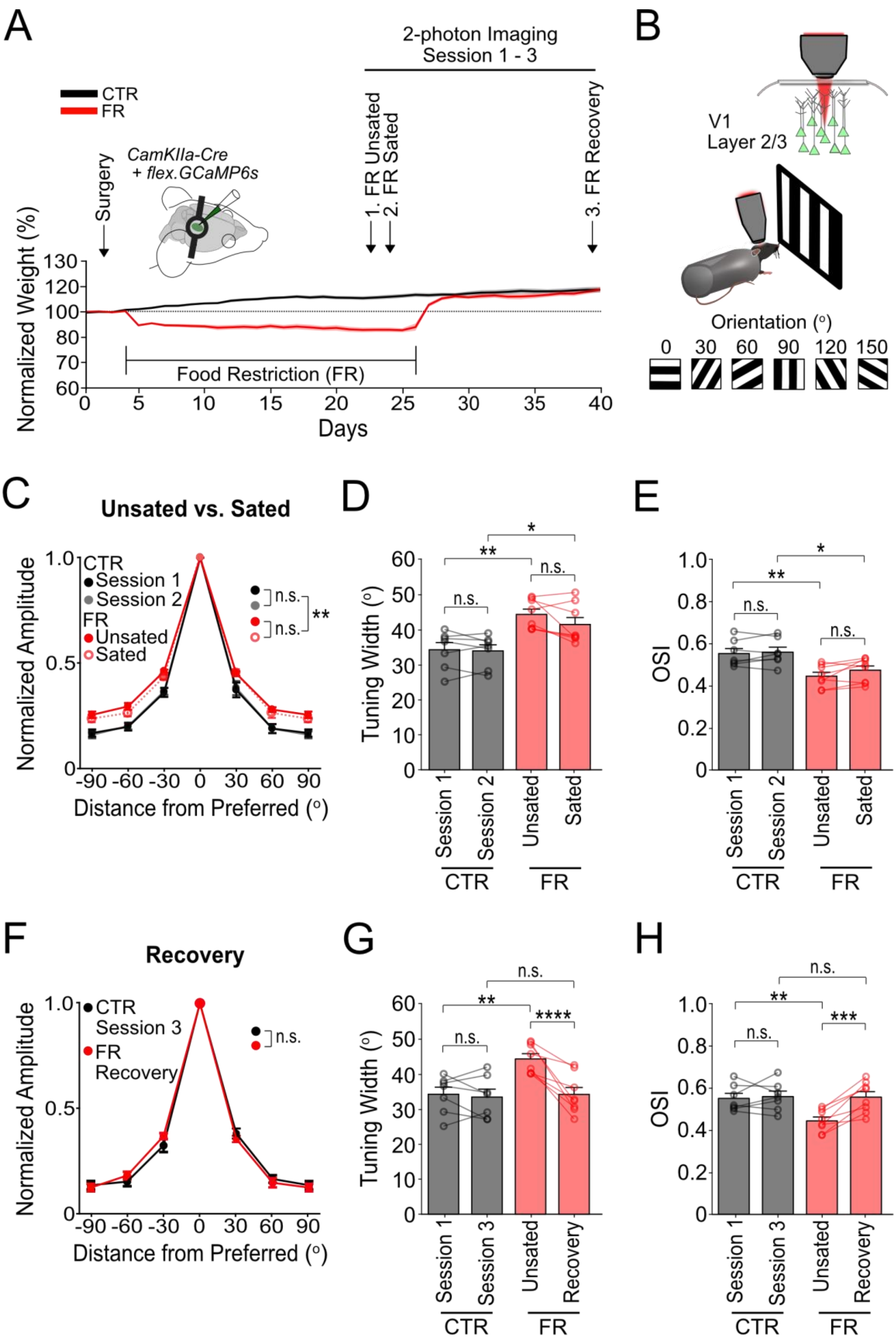

Figure S6: Broadening of orientation tuning under food restriction was not impacted by short-term satiety, and reversed to control values after recovery of bodyweight. Related to Figure 7. (A) Experimental timeline along with animal weight. (**B)** Schema of two-photon imaging during presentation of drifting gratings. (**C)** Mean orientation tuning curves, normalized to the response of

the preferred stimulus. Note that -90° and +90° conditions correspond to the same visual stimulus. (Three-way ANOVA with data from (C) and (F), which includes data from Main Figure 6B; CTR group vs. FR group: p<0.0001; CTR Session 1 vs. CTR Session 2: p>0.99; FR Unsated vs. FR Sated: p=0.32; CTR Session 1 vs. FR Unsated: p<0.001; CTR Session 2 vs. FR Sated: p<0.001; n = 7 CTR and 8 FR mice). (**D)** Mean orientation tuning width for CTR and FR before and after satiety (Two-way Repeated Measures ANOVA run with data in (D) and (G), which includes data from Main Figure 6C, with post hoc Sidak's tests; CTR Session 1 vs. CTR Session 2: p = 0.99; FR Unsated vs. FR Sated: p= 0.17; n = 7 CTR and 8 FR mice). **(E)** Mean orientation selectivity index (OSI, measured as 1-circular variance) for CTR and FR before and after satiety (Two-way Repeated Measures ANOVA run with data in (E) and (H); *post hoc* Sidak's tests; CTR Session 1 vs. CTR Session 2: p = 0.98; FR Unsated vs. FR Sated: p= 0.30; n = 7 CTR and 8 FR mice). (**F)** Mean orientation tuning curves, normalized to the response of the preferred stimulus. (Three-way ANOVA with data from (C) and (F); CTR group vs. FR group: p<0.0001; CTR Session 3 vs. FR Recovery: p>0.99; n = 7 CTR and 8 FR mice). (**G)** Mean orientation tuning widths for CTR and FR after recovery of bodyweight following 10-14 days of *ad libitum* access to food. (Two-way Repeated Measures ANOVA; post hoc Sidak's tests; CTR Session 1 vs. CTR Session 3: p = 0.94; CTR Session 3 vs. FR Recovery: p= 0.98; n = 7 CTR and 8 FR mice). (**H)** Mean orientation selectivity index (OSI, measured as 1-circular variance) for CTR and FR before and after recovery of free feeding bodyweight (Two-way Repeated Measures ANOVA; post hoc Sidak's tests; CTR Session 1 vs. CTR Session 3: p = 0.99; CTR Session 3 vs. FR Recovery: p= 0.99; n = 7 CTR and 8 FR mice). * p< 0.05; ** p<0.01; *** p<0.001; *** p<0.0001. Error bars are S.E.M.

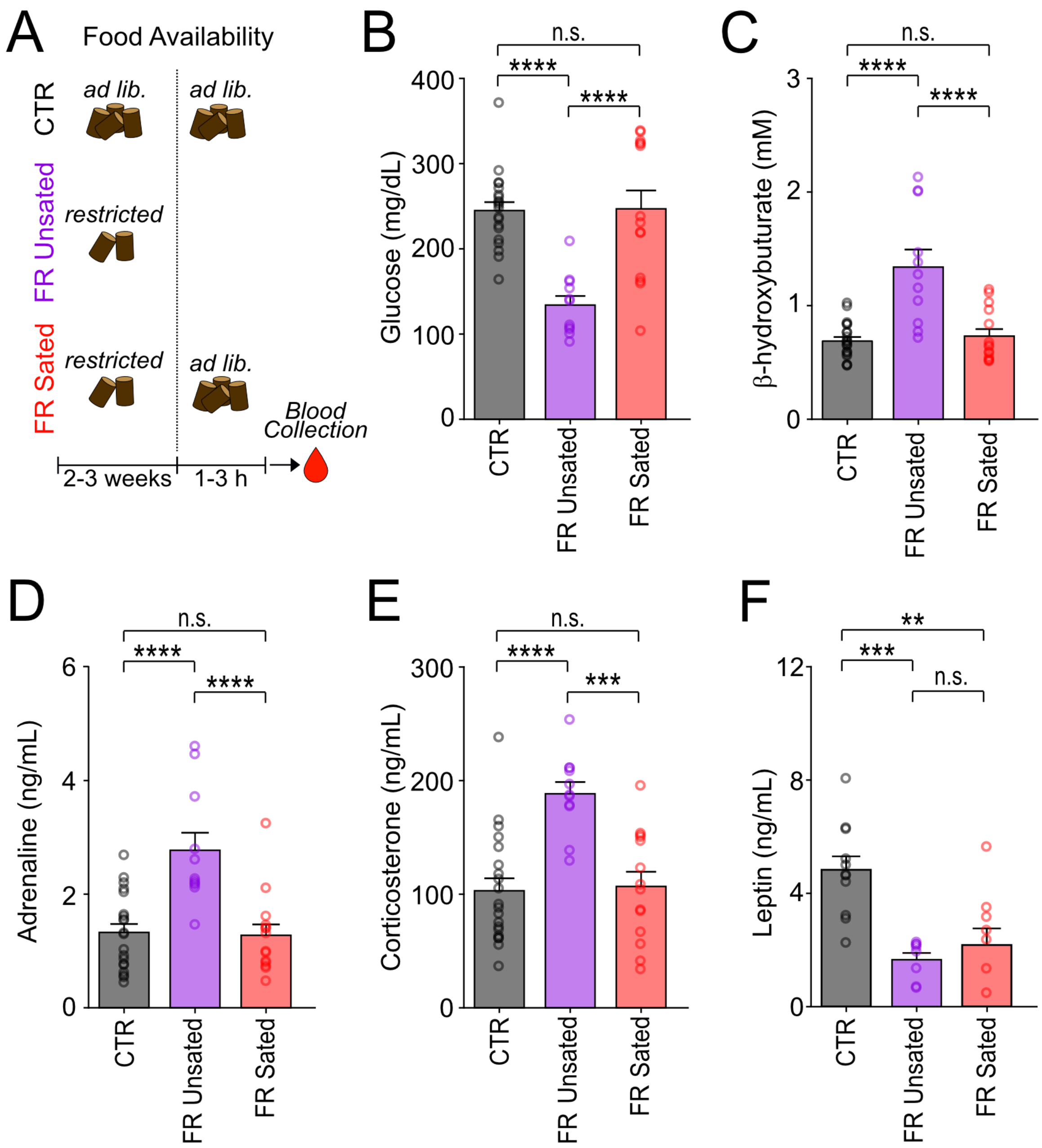

Figure S7. Impact of food restriction and satiety on metabolic state. Related to Figure 7. (A) Experimental schema. Control (CTR) and food-restricted (FR) animals were maintained on *ad libitum* and food-restricted diets (resulting in 15% loss of body weight), respectively, for 2-3 weeks prior to trunk blood collection. Animals in the "FR sated" group were sated with *ad libitum* access to food for 1-3 hours prior to blood collection, "FR unsated" animals were not. **(B)** Mean levels of serum glucose (One-way ANOVA: $p<0.0001$; CTR vs. FR Sated: $p>0.99$; n = 20 CTR, 11 FR Unsated, and 14 FR Sated animals). **(C)** Mean levels of serum β-hydroxybuturate (ketone body) (One-way ANOVA: $p<0.0001$; CTR vs. FR Sated: $p=0.97$; n = 20 CTR, 11 FR Unsated, and 14 FR Sated animals). **(D)** Mean levels of serum adrenaline (One-way ANOVA: $p<0.0001$; CTR vs. FR Sated: $p>0.99$; n = 20 CTR, 11 FR Unsated, and 14 FR Sated animals). **(E)** Mean levels of serum corticosterone (One-way ANOVA: $p<0.0001$; CTR vs. FR Sated: $p>0.99$; n = 20 CTR, 11 FR Unsated, and 14 FR Sated animals). **(F)** Mean levels of serum leptin (One-way ANOVA: $p<0.0001$; CTR vs. FR Sated: $p=0.87$; n = 11 CTR, 8 FR Unsated, and 9 FR Sated animals). Error bars are S.E.M.

Supplemental Table S1 – Model parameters for all groups relative to control. Related to Figure 5.

Group	Depolarization of the V_{Rest} from control	Normalized leak: $g_L^{group}/g_L^{control}$	Normalized synaptic conductance: $g_0^{group}/g_0^{control}$
Control (CTR)	+0 mV	1	1
Increased R_m	+0 mV	0.79	0.79
V_{Rest} depolarized	+5 mV	1	0.81
Food-restricted (FR)	+5 mV	0.79	0.64

Resting membrane potential (V_{Rest}); Resting leak conductance (g_L); Synaptic conductance at rheobase (g_o)

Supplemental Table S2 – Hodgkin-Huxley-type neuron model parameters (control). Related to Figure 5.

Parameters:		
C_m	Membrane capacitance	1 μF/cm^2
g_L	Leak conductance (inverse input resistance)	0.1 mS/cm^2
E_L	Leak reversal potential	-75 mV
E_{rest}	Resting membrane potential	-75 mV
g_{Na}	Na_v maximal conductance	35 mS/cm^2
E_{Na}	Na_v reversal potential	55 mV
g_K	K_v maximal conductance	4 mS/cm^2
E_K	K_v reversal potential	-90 mV
g_X	Subthreshold channel maximal conductance	0.18 mS/cm^2
γ	Single-channel conductance (only for g_X channel)	20 pS
